# Supplementary material for: Nudging towards COVID-19 and influenza vaccination uptake in medically at-risk children: EPIC study protocol of randomised controlled trials in Australian paediatric outpatient clinics
Source: BMJ Open. 2024 Feb 17;14(2):e076194. doi: 10.1136/bmjopen-2023-076194 (PMC10875525; doi:10.1136/bmjopen-2023-076194)
Supplement: Author's manuscript [file bmjopen-2023-076194.draft_revisions.pdf]

BMJ Open

BMJ Open is committed to open peer review. As part of this commitment we make the peer review history of every article we publish publicly available.

When an article is published we post the peer reviewers' comments and the authors' responses online. We also post the versions of the paper that were used during peer review. These are the versions that the peer review comments apply to.

The versions of the paper that follow are the versions that were submitted during the peer review process. They are not the versions of record or the final published versions. They should not be cited or distributed as the published version of this manuscript.

BMJ Open is an open access journal and the full, final, typeset and author-corrected version of record of the manuscript is available on our site with no access controls, subscription charges or pay-per-view fees (http://bmjopen.bmj.com).

If you have any questions on BMJ Open's open peer review process please email info.bmjopen@bmj.com

BMJ Open

Nudging toward COVID-19 and influenza vaccination uptake in medically at risk children – EPIC study protocol of randomised controlled trials in children

Journal:	*BMJ Open*
Manuscript ID	bmjopen-2023-076194
Article Type:	Protocol
Date Submitted by the Author:	31-May-2023
Complete List of Authors:	Wang, Bing; The University of Adelaide, Robinson Research Institute and Adelaide Medical School<br>Andraweera, Prabha ; University of Adelaide School of Medicine, Obstetrics and Gynaecology<br>Danchin, Margaret; Murdoch Childrens Research Institute, Paediatrics<br>Blyth, Christopher C.; The University of Western Australia<br>Vlaev, Ivo; Warwick Business School<br>Ong, Jason ; Monash University, Melbourne Sexual Health Clinic & LSHTM<br>Dodd, Jodie; The University of Adelaide, Obstetrics and Gynaecology<br>Couper, Jennifer; The University of Adelaide<br>Sullivan, Thomas; South Australian Health and Medical Research Institute<br>Karnon, Jonathan; Flinders University, CMPH<br>Spurrier, Nicola; SA Health Department for Health and Wellbeing<br>Cusack, Michael; SA Health Department for Health and Wellbeing<br>Mordaunt, Dylan; Flinders University<br>Simatos, Dimi; Discipline of Paediatrics Lyell McEwin Hospital<br>Dekker, Gustaaf; The University of Adelaide<br>Carlson, Samantha; Telethon Kids Institute, Wesfarmers Centre of Vaccines and Infectious Diseases; The University of Western Australia, School of Social Sciences<br>Tuckerman, Jane; Murdoch Childrens Research Institute, Infection and Immunity<br>Wood, Nicholas; University of New South Wales<br>Whop, Lisa; Australian National University, National Centre for Epidemiology and Population Health<br>Marshall, Helen; The University of Adelaide; The University of Adelaide
Keywords:	COVID-19, INFECTIOUS DISEASES, Public health < INFECTIOUS DISEASES, Primary Prevention

SCHOLARONE™
Manuscripts

TITLE PAGE

Nudging toward COVID-19 and influenza vaccination uptake in medically at risk children – EPIC study protocol of randomised controlled trials in children

Bing Wang[1,2,3], Prabha H Andraweera[1,2,3], Margie Danchin[4,5], Christopher C Blyth[6,7,8], Ivo Vlaev[9], Jason J. Ong[10], Jodie Dodd[2,3,11], Jennifer Couper[2,3,12], Thomas R Sullivan[13,14], Jonathan Karnon[15], Nicola Spurrier[16,17], Michael Cusack[17], Dylan Mordaunt[16], Dimi Simatos[18], Gus Dekker[2,3,19], Samantha Carlson[6], Jane Tuckerman[4,5,], Nicholas Wood[20,21], Lisa Whop[22], Helen S Marshall[1,2,3]

[1] Vaccinology and Immunology Research Trials Unit, Women's and Children's Hospital, SA Health, Adelaide, South Australia

[2] Robinson Research Institute, The University of Adelaide, Adelaide, South Australia, Australia

[3] Adelaide Medical School, The University of Adelaide, Adelaide, South Australia, Australia

[4] Murdoch Childrens Research Institute, Melbourne, Victoria, Australia; The Royal Children's Hospital, Melbourne, Victoria, Australia

[5] Department of Paediatrics, The University of Melbourne, Melbourne, Australia

[6] Wesfarmers Centre of Vaccines and Infectious Diseases, Telethon Kids Institute and School of Medicine, University of Western Australia, Western Australia, Australia

[7] Department of Infectious Diseases, Perth Children's Hospital, Perth, Western Australia, Australia

[8] Department of Microbiology, PathWest Laboratory Medicine, Queen Elizabeth II Medical Centre (QEIIMC), Western Australia, Australia

[9] School of Business, Warwick University, Warwick, United Kingdom

For peer review only - http://bmjopen.bmj.com/site/about/guidelines.xhtml

[10] Central Clinical School, Monash University, Melbourne, Victoria, Australia

[11] Women's and Babies Division, Women's and Children's Hospital, Adelaide, South Australia, Australia

[12] Division of Paediatrics, Women's and Children's Hospital, Adelaide, South Australia, Australia

[13] South Australian Health and Medical Research Institute, South Australia, Australia

[14] School of Public Health, The University of Adelaide, Adelaide, South Australia, Australia

[15] Discipline of Public Health, Flinders University, South Australia, Australia

[16] Discipline of Paediatrics, Flinders University, South Australia, Australia

[17] SA Health, South Australian Government, Australia

[18] Discipline of Paediatrics, Lyell McEwin Hospital, South Australia, Australia

[19] Discipline of Women's Health, Lyell McEwin Hospital, South Australia, Australia

[20] Discipline of Paediatrics, University of Sydney, Sydney, New South Wales, Australia

[21] Children's Hospital Westmead, Sydney, New South Wales, Australia

[22] Discipline of Public Health, Australian National University, Canberra,

Key words: COVID-19, Influenza, vaccine, medically at risk children

Correspondence: Prof. Helen Marshall, Vaccinology and Immunology Research Trials Unit, Women's and Children's Hospital, Adelaide, South Australia, Australia; phone +61 (08) 8313 4086, fax +61 (08) 8313 4086, email helen.marshall@adelaide.edu.au

For peer review only - http://bmjopen.bmj.com/site/about/guidelines.xhtml

ABSTRACT

Introduction: Children with chronic medical diseases are at an unacceptable risk of hospitalisation and death from influenza and coronavirus 2 (SARS-CoV-2) infections. Over the past two decades, behavioural scientists have learnt how to design non-coercive "nudge" interventions to encourage positive health behaviours. Our study aims to evaluate the impact of multi-component nudge interventions on the uptake of coronavirus disease (COVID-19) and influenza vaccines in medically at risk children.

Methods and analyses: Children with chronic medical conditions attending tertiary hospitals in South Australia, Western Australia and Victoria will be enrolled in two separate randomised controlled trials (RCTs). Children who have received less than two doses of any COVID-19 vaccine will be eligible for the COVID-19 RCT. Those who have not received any seasonal influenza vaccine during the influenza season in 2023 will be eligible for the influenza RCT. Participants will be randomly assigned (1:1) to the standard care or intervention group. The nudge intervention in each RCT will consist of three text message reminders with four behavioural nudges including (i) social norm messages, (ii) different messengers through links to short educational videos from a paediatrician, medically at risk child and parent, and nurse, (iii) a pledge to have their child or themselves vaccinated and (iv) information salience through links to the current guidelines and vaccine safety information. The primary outcome is the proportion of medically at risk children who receive at least one dose of vaccine within 3 months of randomisation. Logistic regression analysis will be performed to determine the effect of the intervention on the probability of vaccination uptake.

Ethics and dissemination: The protocol and study documents have been reviewed and approved by the Women's and Children's Health Network Human Research Ethics Committee (HREC/22/WCHN/2022/00082). Results will be published via peer-reviewed journals and presented at scientific meetings and public forums.

For peer review only - http://bmjopen.bmj.com/site/about/guidelines.xhtml

ClinicalTrials.gov Identifier: NCT05613751

Article Summary

Strengths and limitations of this study

- To our knowledge, no studies to date have yet examined the effectiveness of nudge interventions aiming to improve Influenza and COVID-19 vaccine uptake in medically at risk children.
- Nudgeathon, has not been carried out to develop nudge intervention for improving the Influenza and COVID-19 vaccine uptake in medically at risk children.
- Randomised controlled trials are essential to evaluate if the nudge interventions are effective in increasing Influenza and COVID-19 vaccination coverage in hospital settings.
- Waive of consent in our study reduces selection bias.

For peer review only - http://bmjopen.bmj.com/site/about/guidelines.xhtml

INTRODUCTION (word count: 3892)

Children with chronic medical conditions represent a clinically vulnerable group in Australia with around 15% of children having either a respiratory, cardiac or neurological condition that increases their risk of hospitalisation and death from respiratory infections.[1] A systematic review and meta-analysis conducted by our group in 2017 demonstrates that compared with healthy peers, medically at risk children with influenza are at higher risks for intensive care unit (ICU) admission, mechanical ventilation and death.[2] They are also more likely to develop bacterial pneumonia or experience prolonged hospital length of stay.[2] Australian data on children ≤ 16 years admitted with acute respiratory illnesses and tested for influenza at sentinel hospitals between 2010 and 2019 demonstrate that comorbidities are an independent predictor of severe outcomes. Specifically, the odds of ICU admission are higher in those with any comorbidity (adjusted odds ratio (aOR): 1.36, 95% Confidence Interval [95% CI]: 1.05-1.77) compared with cases without any comorbidity. Children with diabetes (aOR: 3.22, 95% CI: 1.25; 8.23), cardiac (aOR: 1.93, 95% CI: 1.23-3.03), respiratory (aOR: 1.54, 95% CI: 1.08-2.21), or neurological comorbidities (aOR: 1.57, 95% CI: 1.25-1.98) are at greatest odds of ICU admission. Respiratory, neurological, cardiac, genetic, and hepatic comorbidities and diabetes were shown to be associated with prolonged hospitalisation, whereas respiratory, renal, cardiac, and other comorbidities increased the length of ICU stay.[3] The cost per episode of influenza-associated hospitalisation was estimated to be AUD $19,704 (95% CI: 11,715-27,693) for children with chronic lung conditions compared to AUD $4,557 (95% CI: 4,129-4,984) for children without.[4] Influenza vaccines are provided free to this group (≥ 6 months).[5] Our research has previously shown that parents of children with chronic medical conditions are less likely to have their children vaccinated unless recommended by their specialist.[6] The uptake of influenza vaccine at least once in the last two years among medically at risk children is 50% with annual vaccination only 32.8%.[7]

For peer review only - http://bmjopen.bmj.com/site/about/guidelines.xhtml

The coronavirus 2 (SARS-CoV-2) infection causing Coronavirus disease (COVID-19) may overall be milder in children compared to adults but a small proportion develop severe disease and are at risk of a debilitating multisystem-inflammatory syndrome (MIS-C) and/or long COVID (long lasting complications of COVID-19).[8] Children with chronic diseases are also at a higher risk of COVID-19 complications including pneumonia and respiratory failure and long-term health consequences.[4,9] Long COVID is a multi-system illness characterised by ongoing persistent symptoms that can last for weeks or months following SARS-CoV-2 infection.[10] Chronic fatigue, short term memory problems, loss of taste and smell and long school absences were reported in long COVID cases.[11] COVID-19 vaccines are provided free to medically at risk children (≥ 6 months).[5] Of concern, two-dose COVID-19 vaccine uptake among children aged 5-11 years is at present approximately 40% nationally.[12] At a time when Australia is still experiencing a large number of SARS-CoV-2 infections, with greater risks to the individual and the health system of co-circulation of influenza and SARS-CoV-2 infections and emergence of new viral variants, it is critical that all efforts are made to improve vaccine uptake among these high-risk groups. Equally important is the opportunity to continue to evaluate the safety of COVID-19 vaccines in these groups including children with chronic conditions that are often excluded from clinical trials.

Lack of access and practical barriers to vaccines remain the leading cause of low vaccine uptake, which is especially relevant to patients whose care is primarily in a hospital setting. For children with chronic conditions the hospital often takes on the role of primary as well as tertiary care due to parental anxiety, life pressures, timeliness and access issues. Parents have identified barriers such as concern and lack of confidence in family physicians providing a vaccine recommendation for medically at risk children and access to vaccines in hospital

For peer review only - http://bmjopen.bmj.com/site/about/guidelines.xhtml

settings being sub-optimal. Mobility, transport, language, financial barriers, and remoteness are also reported barriers for children with chronic and complex conditions in accessing services.[13] For these groups, vaccination in the hospital setting, when patients are already onsite and engaged with a healthcare setting, would improve immunisation opportunities and protection against the threat of both influenza and SARS-CoV-2 infection.

Improving uptake of recommended vaccines in medically at risk children primarily attending hospitals requires an innovative rather than a population approach. Over the past two decades, behavioural scientists have learnt how to design non-coercive "nudge" strategies to promote positive behaviours in a range of contexts. In psychology and neuroscience, a dual process theory describes brain functioning as two types of cognitive processes - 'System 1' processes described as automatic, uncontrolled, effortless, associative, fast, unconscious and affective, and 'System 2' processes described as reflective, controlled, effortful, rule-based, slow, conscious and rational.[14] Although this 'dual process' model is considered as a theoretical basis for nudge theory, the nudge approach suggests that automatic decisions can be systematically triggered to improve health outcomes.[15] In other words, nudges are simple cues in our environment that influence people to behave in a certain way to achieve better personal or social goals without making a conscious decision to do so.[16] Using behavioural economics (a discipline that studies how individuals make choice) the environment in which a choice is being made can actively be designed to encourage better health-related choices.[17] A previous study showed nudges and reminders resulted in a decrease in energy-dense nutrient-poor foods in men and sugar-sweetened beverages in women, together with a reduction in body weight.[18] Nudge-based interventions to address vaccine hesitancy include using reminders and recall, changing the way information is framed and delivered to an intended audience, changing the messenger delivering information, invoking social norms and emotional affect (e.g., through

For peer review only

For peer review only - http://bmjopen.bmj.com/site/about/guidelines.xhtml

storytelling, dramatic narratives and graphical presentations), and offering incentives or changing defaults. Nudge-based interventions show potential to increase vaccine confidence and uptake. The most promising evidence exists for nudges that offer incentives to parents and healthcare workers, that make information more salient, that frame vaccination as the default, or that use trusted messengers to deliver information.[19,20] Normalizing vaccination and peer influence can activate social tendencies to join others.[21] Interestingly, strategies with educational approaches are less effective (or not effective at all) in improving vaccine uptake.[22] A "nudge" intervention needs to be simple and low cost and if proven successful can be simply incorporated into standard health care. For example, redesigning cardiac rehabilitation referral decision pathway from opt-in to opt-out referral, which automatically identified eligible patients from the electronic health record and notified staff on the wards by using secure text messaging, increased cardiac rehabilitation referral rates from 15% to 85%.[23]

However, a challenge is how behavioural economic concepts and principles can be effectively applied by community stakeholders to identify barriers and create an innovative intervention to complement the current immunisation programs. To bridge this gap, a Nudgeathon,[24] a crowdsourcing interaction in which decision groups draw on insights and methods from nudge theory, augmented by design thinking and drama theory to devise implementable solutions to major behavioural policy problems, was launched.[24] These events are typically conducted over 1-2 days, bringing together a diverse range of stakeholders (many who are new to behavioural science) to generate new approaches to complex problems in a time-pressured setting. Three dozen Nudgeathons[24] around the world have been conducted to address multiple issues (e.g. handwashing among health providers, increasing regular HIV/sexually transmitted infection testing in men[17,25]) but to our knowledge, there has not been a Nudgeathon for the immunisation programs. The MINDSPACE framework, which provides a list of behaviour change techniques

For peer review only

For peer review only - http://bmjopen.bmj.com/site/about/guidelines.xhtml

including Messenger, Incentives, Norms, Defaults, Salience, Priming, Affect, Commitments, Ego, that target the automatic decision processes,[26] was used to identify and generate potential nudges at the Nudgeathon.

Therefore, this study aims to design nudge interventions and evaluate the impact of multi-component nudge interventions on the uptake of influenza and COVID-19 vaccines among medically at risk children.

Methods and analysis

Recommendations for Intervention Trials (SPIRIT) checklist was used when developing this protocol.[27] The first participant was randomised on 29 November 2023 2022 and the study is expected to be complete by 31 December 2023. The master protocol was devised to evaluate the impact of multi-component nudge interventions on the uptake of COVID-19 and influenza vaccines in two distinct sub-populations: medically at-risk children and pregnant women, employing a unified and overarching design. The information pertaining to medically at-risk children is presented here.

Nudge intervention

In August 2022, a nudgeathon was held to design appropriate nudges for inclusion in the trial. Fourteen participants including medically at risk children, their parents, paediatricians, nurses, hospital administrative personnel, behavioural scientists, psychologists and graphic designers with diverse skills from South Australia (SA), Western Australia and Victoria participated in the nudgeathon. The participants learnt about behavioural science and successful application of nudges in different disciplines. They were assigned into small groups ensuring that each group comprised participants with different skills. The individual groups were requested to

For peer review only - http://bmjopen.bmj.com/site/about/guidelines.xhtml

create a nudge for improving either COVID-19 or influenza vaccine uptake in medically at risk children. At the end of the nudgeathon each group presented their nudges and one nudge for each condition was chosen by the team to develop the nudge interventions in the following randomised control trials. Some of the most robust effects that have been found to have strong impacts on behaviour[26] such as Messenger, Norms, Salience and Commitments were used to generate nudge interventions in our study. The carefully chosen nudge for both COVID-19 and influenza vaccines consists of three SMS text messages that are sent to parents of medically at risk children from the participating hospitals four weeks apart. The first text message is sent one week prior to a scheduled paediatric clinic appointment and provides a link to current national guidelines on COVID-19 or influenza vaccination in children with chronic illness and a link to a video of a paediatrician explaining the benefits of COVID-19 or influenza vaccination in medically at risk children. The second text message provides a link to a video of a child with chronic illness and their parent giving their opinion on the protection of medically as risk children against SARS-CoV-2 or influenza infections. The third text message provides a link to a video of a nurse discussing the potential adverse outcomes of not being vaccinated against SARS-CoV-2 or influenza infections. The first and second text messages also provide the option for a parent to agree to vaccinate their children or opt out from receiving further reminders.

Primary objectives

- To determine the proportion of medically as risk children in the intervention versus standard care (non-intervention) group receiving at least one dose of the seasonal influenza vaccine within 3 months after randomisation, as assessed using the Australian Immunisation Register (AIR)

For peer review only - http://bmjopen.bmj.com/site/about/guidelines.xhtml

- To determine the proportion of medically as risk children in the intervention versus standard care (non-intervention) group receiving at least one dose of a COVID-19 vaccine within 3 months after randomisation, as assessed using the AIR.

The AIR is a national register that records vaccines given to all people in Australia including vaccines given under the National Immunisation Program, including school programs, and privately, such as for influenza. A recognised vaccination provider including doctors, such as a family physician or community health centre, are required to update the AIR once an influenza, COVID-19 or any vaccine listed in the National Immunisation Program Schedule is administered.

Secondary objectives

- To identify socio-demographic characteristics and medical risk factors associated with COVID-19 or influenza vaccination in medically at risk children
- To assess timeliness of influenza and COVID-19 vaccine uptake among medically at risk children during the study period by determining the proportion of medically at risk children who receive the COVID-19 or influenza vaccine by month throughout the study period.
- To estimate the cost-effectiveness of proven interventions compared to standard care in hospital settings

Study design and study population

These are parallel-group randomised controlled trials (RCTs, ClinicalTrials.gov Identifier: NCT05613751) designed to measure the impact of a nudge intervention on receipt of one dose of the COVID-19 or influenza vaccine in medically at risk children who attend a clinic at

For peer review only - http://bmjopen.bmj.com/site/about/guidelines.xhtml

tertiary hospitals in South Australia, Western Australia and Victoria. Medically at risk children will be enrolled at seven hospitals across South Australia (Women's and Children's Hospital, Flinders Medical Centre and the Lyell McEwin Hospital), Victoria (The Royal Children's Hospital) and Western Australia (Perth Children's Hospital). Each of these hospitals covers different socio-demographic areas and patient sources ensuring our research is generalisable. COVID-19 or influenza vaccination status of medically at risk children attending the participating hospitals will be determined by checking the AIR. The inclusion and exclusion criteria are listed in Table 1.

For peer review only

For peer review only - http://bmjopen.bmj.com/site/about/guidelines.xhtml

278 Table 1 Inclusion and exclusion criteria

	Exclusion criteria	Inclusion criteria
COVID vaccine[28]	• Known contraindications to COVID-19 vaccine<br>• Up to date for COVID-19 vaccine (≥ two doses) at the time of enrolment,<br>• Sibling of a child already enrolled in the trial (only the sibling who is eligible and scheduled to attend a paediatric clinic first will be eligible)	Medically at risk children aged 5 years to 18 years with a cardiac, endocrine, respiratory, gastrointestinal, haematological, musculoskeletal, neurological condition
Influenza vaccine[5]	• Known contraindications to influenza vaccine<br>• Already received an influenza vaccine during the flu season in 2023<br>• Sibling of a child already participating in the trial (the sibling who is eligible and scheduled to attend a paediatric clinic first will be eligible)	Children aged ≥6 months and < 18 years with medical conditions specified in this list:<br>Immunocompromising conditions including:<br>• malignancy<br>• chronic steroid use<br>• haematopoietic stem cell transplant<br>Functional or anatomical asplenia, including:

For peer review only - http://bmjopen.bmj.com/site/about/guidelines.xhtml

	Exclusion criteria	Inclusion criteria
	• Previous participation in the COVID-19 nudge RCT	• sickle cell disease or other haemoglobinopathies<br>• congenital or acquired asplenia (for example, splenectomy) or hyposplenia<br>Cardiac disease, including:<br>• cyanotic congenital heart disease<br>• congestive heart failure<br>• coronary artery disease<br>Chronic respiratory conditions, including:<br>• suppurative lung disease<br>• bronchiectasis<br>• cystic fibrosis<br>• chronic obstructive pulmonary disease<br>• Severe asthma (requiring frequent medical consultations or the use of multiple medicines)

For peer review only - http://bmjopen.bmj.com/site/about/guidelines.xhtml

	Exclusion criteria	Inclusion criteria
		Chronic neurological conditions, including:<br>• hereditary and degenerative CNS diseases<br>• seizure disorders<br>• spinal cord injuries<br>• neuromuscular disorders<br>Chronic metabolic disorders, including:<br>• Type 1 or 2 diabetes<br>• amino acid disorders<br>• carbohydrate disorders<br>• cholesterol biosynthesis disorders<br>• fatty acid oxidation defects<br>• lactic acidosis<br>• mitochondrial disorders<br>• organic acid disorders

For peer review only - http://bmjopen.bmj.com/site/about/guidelines.xhtml

	Exclusion criteria	Inclusion criteria
		• urea cycle disorders<br>• vitamin/cofactor disorders<br>• porphyria<br><br>Chronic renal failure<br><br>Children aged 5 to 10 years receiving long term aspiring therapy<br><br>Down syndrome<br><br>Obesity (body mass index ≥30 kg per m^2)<br><br>Children born less than 37 weeks gestation

For peer review only

For peer review only - http://bmjopen.bmj.com/site/about/guidelines.xhtml

Randomisation

Medically at risk children will be randomised to the intervention and standard care groups in a 1:1 ratio. The randomisation list will be generated using R version 4.02 by an independent statistician who will not be involved with the conduct or analysis of the RCT. Allocations will be performed using randomly permuted blocks, stratified by site. Randomisation will be done on a password protected REDCap database securely held on the University of Adelaide server.

Endpoint measurements will involve low level contact of study staff, however when contact is required (such as AIR/ confirmation of influenza/COVID-19 vaccine receipt etc), this will be carried out by trial staff shielded from information that might reveal trial group assignment. The study statistician undertaking the analysis and study investigators will remain blinded to trial intervention assignment.

Study processes

Medically at risk children attending clinics at the hospitals will be identified from Outpatients' Department's appointment lists. Research nurses will assess COVID-19 or influenza vaccination status of children on the AIR to screen for eligibility. Medically at risk children will be randomised to the intervention arm or the standard care arm on REDCap. Demographic data will be obtained from hospital medical records. Using the Norms, Messenger, Salience and Commitment nudge strategies, parents of medically at risk children randomised to the intervention arm will receive a maximum of three SMS reminders from the hospital using 'Message Media' software from the hospitals. The SMS messages will comprise a brief message that many children with chronic illness obtain the COVID-19 or influenza vaccine (Norms) and a reminder for the child to obtain the vaccine. Parents of medically at risk children have the option of responding to the message by 1) agreeing to obtain the vaccine

For peer review only - http://bmjopen.bmj.com/site/about/guidelines.xhtml

(Commitment), 2) stating that the participant has already received the vaccine or 3) requesting to opt out from receiving further reminders (first and second SMS messages). Each S-MS message also provides a link to a video of (Messager): 1) a paediatrician discussing the potential serious health consequences of SARS-CoV-2 or influenza infections (first SMS), 2) a medically at risk child and their parent stating that the child received the vaccine and that the child is protected from serious adverse effects of SARS-CoV-2 or influenza infections (second SMS), 3) a nurse stating the benefits of COVID-19 or influenza vaccination for medically at risk children (third SMS). In addition, the first SMS also states children with special medical conditions are at increased risk of severe COVID-19 or Influenza disease and provides a link to the current guidelines and safety information about COVID-19 or influenza vaccination (Salience). The first SMS is sent approximately one week prior to a scheduled clinic visit. The second SMS message is sent four weeks after the first message to those who have not received a dose of COVID-19 or influenza vaccine after the first SMS (confirmed on AIR) and not requested to opt out. The third SMS message is sent four weeks after the second message to those who have not received a dose of COVID-19 or influenza vaccine after the second SMS (confirmed on AIR) and not requested to opt out. The AIR will be checked at 3 months after randomisation to assess whether medically at risk children have received a dose of COVID-19 or influenza vaccine. All data will be stored securely on a password protected REDCap database held by the University of Adelaide. All data will be de-identified prior to presentation and publication.

Study monitoring and surveillance

The nudge is a behavioural change intervention and hence there are no risks related to invasive procedures or investigational medications. Any risks of mental distress associated with receiving the text message is expected to be very low and the parents have the option of opting

For peer review only - http://bmjopen.bmj.com/site/about/guidelines.xhtml

out from receiving the further text messages after the first one. However, a risk assessment and management plan has been developed for this study. Moreover, the study management committee comprising the chief investigator, site investigators, study coordinators and statistician will carefully monitor the progress of the study.

Public involvement, ethics and dissemination

Stakeholders including medically at risk children and their parents participated in the nudgeathon to develop the nudge intervention. The current protocol version 2.0 (05-01-2023) and all study material have been reviewed and approved by the Women's and Children's Health Network Human Research Ethics Committee (HREC/2022/00082) and research governance approval has been obtained from the Women's and Children's Hospital, Flinders Medical Centre, and Lyell McEwin Hospital in SA, The Royal Children's Hospital in VIC, and Perth Children's Hospital in Western Australia. The written consent was obtained from all stakeholders including health service leaders and clinicians and health consumers to participate in the Nudgeathons. A waiver of consent was approved for medically at risk children to participate in this trial and receive SMS messages in accordance with the National Statement on Ethical Conduct in Human Research. Three criteria guide this. Firstly, there are no estimated risks or harms associated with study procedures. Any risk of psychosocial distress associated with receiving the nudge interventions is very unlikely. Secondly, it would not be possible to obtain informed consent without threatening the validity of the trials. It would be impossible to assess the effectiveness of the nudge by recruiting parents of medically at risk children and informing them about the study and then randomising them not to receive the nudge. Thirdly, as influenza and COVID-19 vaccines are recommended for all medically at risk children aged ≥ 6 months, all data for the influenza and COVID-19 RCTs will be collected as part of routine care, including patient demographics and specialties seen. In addition this methodology was

For peer review only - http://bmjopen.bmj.com/site/about/guidelines.xhtml

approved and successfully applied in the Flutext-4U study.[29] We identified a significant difference in uptake of influenza vaccine in medically at risk children in the Flutext-4U study, likely due to this being a population inclusive study unaffected by healthy selection bias.[30] The study is being conducted in accordance with the Declaration of Helsinki and the International Conference on Harmonization Guidelines on Good Clinical Practice. The trial is being conducted in compliance with the current version of the protocol. Any change to the protocol that affects scientific content, study design, and participating will be considered an amendment and will be submitted to HREC for review and approval prior to implementation.

Participant confidentiality is strictly held in trust by the investigators. Therefore, study documentation, data, and all other information generated will be held in strict confidence. No information concerning the study, or the data will be released to any unauthorised third party. Study participant research data, which is for purposes of statistical analysis and scientific reporting, will be transmitted to and stored at the Women's and Children's Hospital. At the end of the study, all study databases will be de-identified and archived at the University of Adelaide. After the completion of the study, the de-identified and aggregated results will be presented at scientific forums and submitted for publication in peer-reviewed journals. The results will be disseminated regardless of the direction of effect. The findings of the study will be communicated to key stakeholders.

Sample size and statistical analysis plan

To detect an increase in the vaccination rate from ~50% in the control group to 60% in the intervention group, which is considered a clinically relevant impact, with 90% power (two-sided test with alpha = 0.05), a sample size of n = 519 per group (1038 total) is required. Should the vaccination rate in the control arm be higher than 50% or lower than 40%, this sample size

For peer review only - http://bmjopen.bmj.com/site/about/guidelines.xhtml

will still provide at least 90% power to detect a 10% absolute increase in vaccination with the nudge intervention. We aim to enrol at least 1038 medically at risk children to each of the two RCTs across the participating hospitals.

Statistical analyses will be conducted on an Intention To Treat basis according to a pre-specified statistical analysis plan. Baseline characteristics including age, socio-economic status based on postcode, and ethnicity will be reported for each group using percentages, means with standard deviations or medians with ranges as appropriate. The vaccination rate will be compared between intervention and control groups using logistic regression, with adjustment made for participating hospitals. Intervention effects will be reported as odds ratios with 95% confidence intervals. Patient characteristics associated with vaccination will be investigated using multivariable logistic regression models. Cost-effectiveness analysis of the nudge intervention will be compared to standard care from the healthcare payer perspective. The primary cost-effectiveness analysis will estimate the incremental cost per additional person vaccinated, in each of the two RCTs. Secondary analyses for the trial focused on influenza vaccination will estimate the cost per quality-adjusted life year (QALY) gained. The study results will be used to estimate the impact of the nudge intervention on health-related quality of life by calculating the QALYs gained as a result of the increased vaccine uptake.. Implementation costs will be obtained from the study budget and costs related to research activities will be excluded. Estimated cost offsets to the health system associated with influenza related disease(e.g. hospitalisations and emergency visits) will be derived from the literature and calculated using cost weights for Australian Refined Diagnosis Related Groups (AR-DRGs). The cost-effectiveness evaluation will follow standard reporting guidelines in the CHEERS statement.[31]

For peer review only - http://bmjopen.bmj.com/site/about/guidelines.xhtml

DISCUSSION

To our knowledge, this will be the first study assessing the effectiveness and cost-effectiveness of a co-designed multi-component nudge intervention on improving influenza and COVID-19 vaccine uptake in medically at-risk children. Vaccine hesitancy and access barriers are a major barrier that reduces the progress of management of vaccine preventable diseases in children. Identifying solutions to improving COVID-19 and influenza vaccine uptake in medically at risk children is critical, as these children are more vulnerable to severe COVID-19 and influenza than healthy children.

Recent studies were conducted in older adult populations and found behavioural nudges, electronically delivered letters or centralized written reminders, significantly increased influenza vaccination uptake across Denmark and Finland.[32,33] An RCT of a nudge intervention that included text message reminders demonstrated that the first reminder increased appointment and COVID-19 vaccination rates by 6.07% and 3.57% and that the second reminder increased those by 1.65% and 1.06% respectively in the early stages of the COVID-19 vaccine rollout.[34] Another RCT was conducted a few months later in a younger population (mean age 39 years) and no SMS message did substantially better or worse than the control whether vaccination rates were measured one week after the messages were sent or at the end of the study period.[35] The difference between two studies may suggest that nudges help early in vaccination campaigns, but the efficacy decays. However, nudging-based interventions have shown potential to increase vaccine confidence and uptake in many studies, but further evidence is needed for the development and evaluation of clear recommendations.[20]

Our team recently conducted an RCT from April 15 to September 30, 2021 and found a 47% relative increase in uptake of influenza vaccine in medically at risk children using a simple

For peer review only - http://bmjopen.bmj.com/site/about/guidelines.xhtml

430 SMS nudge co-designed with paediatricians.[30] If our text based nudges with links to short
431 educational videos prove to be successful in improving influenza and COVID-19 vaccine
432 uptake in medically at risk children, they can easily be implemented as a promising, valuable,
433 low-cost and long-term tool at a national level.
434

For peer review only

For peer review only - http://bmjopen.bmj.com/site/about/guidelines.xhtml

AUTHORS CONTRIBUTIONS

All authors contributed to protocol development and manuscript revisions. BW wrote the first draft of the manuscript. All authors reviewed and approved the final manuscript for publication.

ACKNOWLEDGEMENTS

We acknowledge the following study coordinators for their contribution to study set up and recruitment: Joanne Koch (Lyell McEwin Hospital), Lauren Thompson (Flinders Medical Centre), Ashleigh Rak (Royal Children's Hospital), Deborah Pidd (Mercy Hospital for Women), Louisa Paparo, Rebecca Pavlos and Erin Van Der Helder (Perth Children's Hospital).

FUNDING STATEMENT

This work is supported by the National Health and medical Research Council of Australia [NHMRC Partnership grant APP2014684]. Additional in-kind funding is provided by the Department of Health, South Australia Government; Department of Health, Victorian Government; Department of Health, Western Australian Government; Women's and Children's Health Network; Southern Adelaide Local Health Network; Northern Adelaide Local Health Network; Women's and Children's Hospital Foundation; Department of Trade and Investment, South Australian Government.

COMPETING INTERESTS STATEMENT

HM acknowledges support from the National Health and Medical Research Council of Australia: Practitioner Fellowship (APP1155066). CCB acknowledges support from the National Health and Medical Research Council of Australia: Investigator Grant (APP1173163). HM is an independent investigator on clinical trials of investigational vaccines manufactured by pharmaceutical companies including Pfizer, ILiAD Biotechnologies, and

For peer review only

For peer review only - http://bmjopen.bmj.com/site/about/guidelines.xhtml

460 Merck. The institution at which HM, BW and PA are employed has received funding for investigator-led research from GlaxoSmithKline, Sanofi-Pasteur, and Pfizer Vaccines. All authors receive no personal payments from industry. There are no other conflicts of interest to declare.

For peer review only

For peer review only - http://bmjopen.bmj.com/site/about/guidelines.xhtml

REFERENCES

1. https://www.aihw.gov.au/reports/children-youth/australias-children/contents/health/chronic-conditions-and-burden-of-disease. Australia's Children: in brief. DOI: 10.25816/5ebca4d0fa7dd
2. Tuckerman J, Misan S, Crawford NW, Marshall HS. Influenza in Children With Special Risk Medical Conditions: A Systematic Review and Meta-analysis. The Pediatric infectious disease journal. 2019 Sep;38(9):912-9. DOI: 10.1097/inf.0000000000002405
3. Norman DA, Cheng AC, Macartney KK, Moore HC, Danchin M, Seale H, et al. Influenza hospitalizations in Australian children 2010-2019: The impact of medical comorbidities on outcomes, vaccine coverage, and effectiveness. Influenza and other respiratory viruses. 2022 Mar;16(2):316-27. DOI: 10.1111/irv.12939
4. Homaira N, Briggs N, Oei JL, Hilder L, Bajuk B, Snelling T, et al. Impact of influenza on hospitalization rates in children with a range of chronic lung diseases. Influenza and other respiratory viruses. 2019 May;13(3):233-9. DOI: 10.1111/irv.12633
5. Immunisation ATAGo. The Australian immunisation handbook. Australian Immunisation Handbook. 2022.
6. Tuckerman J, Crawford NW, Marshall HS. Disparities in parental awareness of children's seasonal influenza vaccination recommendations and influencers of vaccination. PloS one. 2020;15(4):e0230425. DOI: 10.1371/journal.pone.0230425
7. Tuckerman J, Misan S, Salih S, Joseph Xavier B, Crawford NW, Lynch J, et al. Influenza vaccination: Uptake and associations in a cross-sectional study of children with special risk medical conditions. Vaccine. 2018 Dec 18;36(52):8138-47. DOI: 10.1016/j.vaccine.2018.09.039
8. Sudre CH, Murray B, Varsavsky T, Graham MS, Penfold RS, Bowyer RC, et al. Attributes and predictors of long COVID. Nature medicine. 2021 Apr;27(4):626-31. DOI: 10.1038/s41591-021-01292-y
9. Moeller A, Thanikkel L, Duijts L, Gaillard EA, Garcia-Marcos L, Kantar A, et al. COVID-19 in children with underlying chronic respiratory diseases: survey results from 174 centres. ERJ open research. 2020 Oct;6(4). DOI: 10.1183/23120541.00409-2020
10. Health AIo, Welfare. Long COVID in Australia – a review of the literature. Canberra: AIHW, 2022.
11. Ludvigsson JF. Case report and systematic review suggest that children may experience similar long-term effects to adults after clinical COVID-19. Acta paediatrica (Oslo, Norway : 1992). 2021 Mar;110(3):914-21. DOI: 10.1111/apa.15673
12. https://www.health.gov.au/sites/default/files/2023-03/covid-19-vaccine-rollout-update-3-march-2023.pdf. COVID-19 Vaccine Roll-out. DOI: 10.25816/5ebca4d0fa7dd
13. Pearce A, Marshall H, Bedford H, Lynch J. Barriers to childhood immunisation: Findings from the Longitudinal Study of Australian Children. Vaccine. 2015 Jun 26;33(29):3377-83. DOI: 10.1016/j.vaccine.2015.04.089
14. Vlaev I, King D, Dolan P, Darzi A. The Theory and Practice of "Nudging": Changing Health Behaviors. Public Administration Review. 2016;76(4):550-61. DOI: https://doi.org/10.1111/puar.12564
15. Marteau TM, Ogilvie D, Roland M, Suhrcke M, Kelly MP. Judging nudging: can nudging improve population health? BMJ (Clinical research ed). 2011;342:d228. DOI: 10.1136/bmj.d228
16. Simon C, Tagliabue M. Feeding the behavioral revolution: Contributions of behavior analysis to nudging and vice versa. Journal of Behavioral Economics for Policy. 2018;2(1):91-7.

For peer review only - http://bmjopen.bmj.com/site/about/guidelines.xhtml

17. Ong JJ, Chow EPF, Read D, Taj U, Lee D, Vlaev I. Nudgeathons to control HIV: designing strategies using behavioural economics. AIDS. 2020 Dec 1;34(15):2337-40. DOI: 10.1097/QAD.0000000000002693

18. Kerr DA, Harray AJ, Pollard CM, Dhaliwal SS, Delp EJ, Howat PA, et al. The connecting health and technology study: a 6-month randomized controlled trial to improve nutrition behaviours using a mobile food record and text messaging support in young adults. Int J Behav Nutr Phys Act. 2016 Apr 21;13:52. DOI: 10.1186/s12966-016-0376-8

19. Tentori K, Pighin S, Giovanazzi G, Grignolio A, Timberlake B, Ferro A. Nudging COVID-19 Vaccine Uptake by Changing the Default: A Randomized Controlled Trial. Med Decis Making. 2022 Aug;42(6):837-41. DOI: 10.1177/0272989X221101536

20. Renosa MDC, Landicho J, Wachinger J, Dalglish SL, Barnighausen K, Barnighausen T, et al. Nudging toward vaccination: a systematic review. BMJ Glob Health. 2021 Sep;6(9). DOI: 10.1136/bmjgh-2021-006237

21. Sasaki S, Saito T, Ohtake F. Nudges for COVID-19 voluntary vaccination: How to explain peer information? Soc Sci Med. 2022 Jan;292:114561. DOI: 10.1016/j.socscimed.2021.114561

22. Dubé E, Gagnon D, MacDonald NE. Strategies intended to address vaccine hesitancy: Review of published reviews. Vaccine. 2015 Aug 14;33(34):4191-203. DOI: 10.1016/j.vaccine.2015.04.041

23. Adusumalli S, Jolly E, Chokshi NP, Gitelman Y, Rareshide CAL, Kolansky DM, et al. Referral Rates for Cardiac Rehabilitation Among Eligible Inpatients After Implementation of a Default Opt-Out Decision Pathway in the Electronic Medical Record. JAMA network open. 2021 Jan 4;4(1):e2033472. DOI: 10.1001/jamanetworkopen.2020.33472

24. Nudgeathon. [cited 27 March 2023]. Available from: https://www.nudgeathon.co.uk.

25. Tran J, Vlaev I, Read D, Schmidtke KA, Chow EP, Lee D, et al. A qualitative evaluation of a Nudgeathon event for the co-design of sexual health campaign images targeting overseas-born men who have sex with men. Sexual Health. 2023.

26. Dolan P, Hallsworth M, Halpern D, King D, Vlaev I. MINDSPACE: influencing behaviour for public policy. 2010.

27. Chan AW, Tetzlaff JM, Gotzsche PC, Altman DG, Mann H, Berlin JA, et al. SPIRIT 2013 explanation and elaboration: guidance for protocols of clinical trials. BMJ (Clinical research ed). 2013 Jan 8;346:e7586. DOI: 10.1136/bmj.e7586

28. https://www.health.gov.au/initiatives-and-programs/covid-19-vaccines/advice-for-providers/clinical-guidance/clinical-features.

29. Tuckerman J, Harper K, Sullivan TR, Fereday J, Couper J, Smith N, et al. Using provider-parent strategies to improve influenza vaccination in children and adolescents with special risk medical conditions: a randomised controlled trial protocol. BMJ open. 2022 Feb 10;12(2):e053838. DOI: 10.1136/bmjopen-2021-053838

30. Tuckerman J, Harper K, Sullivan TR, Cuthbert AR, Fereday J, Couper J, et al. Short Message Service Reminder Nudge for Parents and Influenza Vaccination Uptake in Children and Adolescents With Special Risk Medical Conditions: The Flutext-4U Randomized Clinical Trial. JAMA pediatrics. 2023 Feb 20. DOI: 10.1001/jamapediatrics.2022.6145

31. Husereau D, Drummond M, Augustovski F, de Bekker-Grob E, Briggs AH, Carswell C, et al. Consolidated Health Economic Evaluation Reporting Standards 2022 (CHEERS 2022) Statement: Updated Reporting Guidance for Health Economic Evaluations. Value Health. 2022 Jan;25(1):3-9. DOI: 10.1016/j.jval.2021.11.1351

32. Johansen ND, Vaduganathan M, Bhatt AS, Lee SG, Modin D, Claggett BL, et al. Nationwide Utilization of Danish Government Electronic Letter System for Increasing InFLUenza Vaccine Uptake (NUDGE-FLU): Study Protocol for a Nationwide Randomized Implementation Trial. Am Heart J. 2023 Feb 16. DOI: 10.1016/j.ahj.2023.02.009

For peer review only

For peer review only - http://bmjopen.bmj.com/site/about/guidelines.xhtml

33. Saaksvuori L, Betsch C, Nohynek H, Salo H, Sivela J, Bohm R. Information nudges for influenza vaccination: Evidence from a large-scale cluster-randomized controlled trial in Finland. PLoS Med. 2022 Feb;19(2):e1003919. DOI: 10.1371/journal.pmed.1003919
34. Dai H, Saccardo S, Han MA, Roh L, Raja N, Vangala S, et al. Behavioural nudges increase COVID-19 vaccinations. Nature. 2021 Sep;597(7876):404-9. DOI: 10.1038/s41586-021-03843-2
35. Rabb N, Swindal M, Glick D, Bowers J, Tomasulo A, Oyelami Z, et al. Evidence from a statewide vaccination RCT shows the limits of nudges. Nature. 2022 2022/04/01;604(7904):E1-E7. DOI: 10.1038/s41586-022-04526-2

For peer review only

For peer review only - http://bmjopen.bmj.com/site/about/guidelines.xhtml

STANDARD PROTOCOL ITEMS: RECOMMENDATIONS FOR INTERVENTIONAL TRIALS

SPIRIT 2013 Checklist: Recommended items to address in a clinical trial protocol and related documents*

Section/item	Item No	Description	Page and Line Number	Reason if not applicable
**Administrative information**				
Title	1	Descriptive title identifying the study design, population, interventions, and, if applicable, trial acronym	P1, L3	
Trial registration	2a	Trial identifier and registry name. If not yet registered, name of intended registry	P4, L82	
	2b	All items from the World Health Organization Trial Registration Data Set	NA	Registered at clinicaltrials.gov.
Protocol version	3	Date and version identifier	P19, L337	
Funding	4	Sources and types of financial, material, and other support	P24, L445-452	
Roles and responsibilities	5a	Names, affiliations, and roles of protocol contributors	P24, L435-437	
	5b	Name and contact information for the trial sponsor	NA	Investigator led study
	5c	Role of study sponsor and funders, if any, in study design; collection, management, analysis, and interpretation of data; writing of the report; and the decision to submit the report for publication, including whether they will have ultimate authority over any of these activities	NA	Investigator led study
	5d	Composition, roles, and responsibilities of the coordinating centre, steering committee, endpoint adjudication committee, data management team, and other individuals or groups overseeing the trial, if applicable (see Item 21a for data monitoring committee)	P18-19, L327-333	
**Introduction**				

For peer review only - http://bmjopen.bmj.com/site/about/guidelines.xhtml

Background and rationale	6a	Description of research question and justification for undertaking the trial, including summary of relevant studies (published and unpublished) examining benefits and harms for each intervention	P5-9, L97-202	
	6b	Explanation for choice of comparators	P7-8, L172-182	
Objectives	7	Specific objectives or hypotheses	P10-11, L241-264	
Trial design	8	Description of trial design including type of trial (eg, parallel group, crossover, factorial, single group), allocation ratio, and framework (eg, superiority, equivalence, noninferiority, exploratory)	P11-12, L267-277	
**Methods: Participants, interventions, and outcomes**				
Study setting	9	Description of study settings (eg, community clinic, academic hospital) and list of countries where data will be collected. Reference to where list of study sites can be obtained	P11-12, L267-273	
Eligibility criteria	10	Inclusion and exclusion criteria for participants. If applicable, eligibility criteria for study centres and individuals who will perform the interventions (eg, surgeons, psychotherapists)	P13-16, Table 1	
Interventions	11a	Interventions for each group with sufficient detail to allow replication, including how and when they will be administered	P17-18, L294-324	
	11b	Criteria for discontinuing or modifying allocated interventions for a given trial participant (eg, drug dose change in response to harms, participant request, or improving/worsening disease)	P18-19, L327-333	
	11c	Strategies to improve adherence to intervention protocols, and any procedures for monitoring adherence (eg, drug tablet return, laboratory tests)	P19, L342-352	
	11d	Relevant concomitant care and interventions that are permitted or prohibited during the trial	NA	Normal care as the intervention is a text message

For peer review only - http://bmjopen.bmj.com/site/about/guidelines.xhtml

Outcomes	12	Primary, secondary, and other outcomes, including the specific measurement variable (eg, systolic blood pressure), analysis metric (eg, change from baseline, final value, time to event), method of aggregation (eg, median, proportion), and time point for each outcome. Explanation of the clinical relevance of chosen efficacy and harm outcomes is strongly recommended	P21, L384-403
Participant timeline	13	Time schedule of enrolment, interventions (including any run-ins and washouts), assessments, and visits for participants. A schematic diagram is highly recommended (see Figure)	P9, L206-207; P17-18, L304-324
Sample size	14	Estimated number of participants needed to achieve study objectives and how it was determined, including clinical and statistical assumptions supporting any sample size calculations	P20-21, L376-382
Recruitment	15	Strategies for achieving adequate participant enrolment to reach target sample size	P19, L342-353

Methods: Assignment of interventions (for controlled trials)

Allocation:

Sequence generation	16a	Method of generating the allocation sequence (eg, computer-generated random numbers), and list of any factors for stratification. To reduce predictability of a random sequence, details of any planned restriction (eg, blocking) should be provided in a separate document that is unavailable to those who enrol participants or assign interventions	P17, L281-285
Allocation concealment mechanism	16b	Mechanism of implementing the allocation sequence (eg, central telephone; sequentially numbered, opaque, sealed envelopes), describing any steps to conceal the sequence until interventions are assigned	P17, L281-285
Implementation	16c	Who will generate the allocation sequence, who will enrol participants, and who will assign participants to interventions	P17, L281-285

For peer review only - http://bmjopen.bmj.com/site/about/guidelines.xhtml

Blinding (masking)	17a	Who will be blinded after assignment to interventions (eg, trial participants, care providers, outcome assessors, data analysts), and how	P17, L287-291
	17b	If blinded, circumstances under which unblinding is permissible, and procedure for revealing a participant's allocated intervention during the trial	P17, L287-291
**Methods: Data collection, management, and analysis**			
Data collection methods	18a	Plans for assessment and collection of outcome, baseline, and other trial data, including any related processes to promote data quality (eg, duplicate measurements, training of assessors) and a description of study instruments (eg, questionnaires, laboratory tests) along with their reliability and validity, if known. Reference to where data collection forms can be found, if not in the protocol	P18, L321-324
	18b	Plans to promote participant retention and complete follow-up, including list of any outcome data to be collected for participants who discontinue or deviate from intervention protocols	P19, L342-352
Data management	19	Plans for data entry, coding, security, and storage, including any related processes to promote data quality (eg, double data entry; range checks for data values). Reference to where details of data management procedures can be found, if not in the protocol	P18, L322-324
Statistical methods	20a	Statistical methods for analysing primary and secondary outcomes. Reference to where other details of the statistical analysis plan can be found, if not in the protocol	P21, L380-399
	20b	Methods for any additional analyses (eg, subgroup and adjusted analyses)	P21, L384-403
	20c	Definition of analysis population relating to protocol non-adherence (eg, as randomised analysis), and any statistical methods to handle missing data (eg, multiple imputation)	P21, L384-385
**Methods: Monitoring**			

For peer review only - http://bmjopen.bmj.com/site/about/guidelines.xhtml

Data monitoring	21a	Composition of data monitoring committee (DMC); summary of its role and reporting structure; statement of whether it is independent from the sponsor and competing interests; and reference to where further details about its charter can be found, if not in the protocol. Alternatively, an explanation of why a DMC is not needed	P19, 332-333
	21b	Description of any interim analyses and stopping guidelines, including who will have access to these interim results and make the final decision to terminate the trial	P19, 330
Harms	22	Plans for collecting, assessing, reporting, and managing solicited and spontaneously reported adverse events and other unintended effects of trial interventions or trial conduct	P18, L327-329
Auditing	23	Frequency and procedures for auditing trial conduct, if any, and whether the process will be independent from investigators and the sponsor	P19, 332-333
**Ethics and dissemination**			
Research ethics approval	24	Plans for seeking research ethics committee/institutional review board (REC/IRB) approval	P19, L337-342
Protocol amendments	25	Plans for communicating important protocol modifications (eg, changes to eligibility criteria, outcomes, analyses) to relevant parties (eg, investigators, REC/IRBs, trial participants, trial registries, journals, regulators)	P20, L358-360
Consent or assent	26a	Who will obtain informed consent or assent from potential trial participants or authorised surrogates, and how (see Item 32)	P19, L342-352
	26b	Additional consent provisions for collection and use of participant data and biological specimens in ancillary studies, if applicable	NA

view only

For peer review only - http://bmjopen.bmj.com/site/about/guidelines.xhtml

Confidentiality	27	How personal information about potential and enrolled participants will be collected, shared, and maintained in order to protect confidentiality before, during, and after the trial	P18, L322-324; P20, L364-370	
Declaration of interests	28	Financial and other competing interests for principal investigators for the overall trial and each study site	P24-25, L454-463	
Access to data	29	Statement of who will have access to the final trial dataset, and disclosure of contractual agreements that limit such access for investigators	P20, L364-370	
Ancillary and post-trial care	30	Provisions, if any, for ancillary and post-trial care, and for compensation to those who suffer harm from trial participation	NA	
Dissemination policy	31a	Plans for investigators and sponsor to communicate trial results to participants, healthcare professionals, the public, and other relevant groups (eg, via publication, reporting in results databases, or other data sharing arrangements), including any publication restrictions	P20, 364-373	
	31b	Authorship eligibility guidelines and any intended use of professional writers	P24, L436-437	
	31c	Plans, if any, for granting public access to the full protocol, participant-level dataset, and statistical code	P20, L372-373	
**Appendices**				
Informed consent materials	32	Model consent form and other related documentation given to participants and authorised surrogates	NA	A consent waiver was approved by ethics committee
Biological specimens	33	Plans for collection, laboratory evaluation, and storage of biological specimens for genetic or molecular analysis in the current trial and for future use in ancillary studies, if applicable	NA	No biological samples are collected.

*It is strongly recommended that this checklist be read in conjunction with the SPIRIT 2013 Explanation & Elaboration for important clarification on the items. Amendments to the protocol should be tracked and dated. The SPIRIT checklist is copyrighted by the SPIRIT Group under the Creative Commons "Attribution-NonCommercial-NoDerivs 3.0 Unported" license.

For peer review only - http://bmjopen.bmj.com/site/about/guidelines.xhtml

BMJ Open

Nudging toward COVID-19 and influenza vaccination uptake in medically at risk children – EPIC study protocol of randomised controlled trials in Australian paediatric outpatient clinics

Journal:	*BMJ Open*
Manuscript ID	bmjopen-2023-076194.R1
Article Type:	Protocol
Date Submitted by the Author:	01-Nov-2023
Complete List of Authors:	Wang, Bing; The University of Adelaide, Robinson Research Institute and Adelaide Medical School<br>Andraweera, Prabha ; University of Adelaide School of Medicine, Obstetrics and Gynaecology<br>Danchin, Margaret; Murdoch Childrens Research Institute, Paediatrics<br>Blyth, Christopher C.; The University of Western Australia<br>Vlaev, Ivo; Warwick Business School<br>Ong, Jason ; Monash University, Melbourne Sexual Health Clinic & LSHTM<br>Dodd, Jodie; The University of Adelaide, Obstetrics and Gynaecology<br>Couper, Jennifer; The University of Adelaide<br>Sullivan, Thomas; South Australian Health and Medical Research Institute<br>Karnon, Jonathan; Flinders University, CMPH<br>Spurrier, Nicola; SA Health Department for Health and Wellbeing<br>Cusack, Michael; SA Health Department for Health and Wellbeing<br>Mordaunt, Dylan; Flinders University<br>Simatos, Dimi; Discipline of Paediatrics Lyell McEwin Hospital<br>Dekker, Gustaaf; The University of Adelaide<br>Carlson, Samantha; Telethon Kids Institute, Wesfarmers Centre of Vaccines and Infectious Diseases; The University of Western Australia, School of Social Sciences<br>Tuckerman, Jane; Murdoch Childrens Research Institute, Infection and Immunity<br>Wood, Nicholas; University of New South Wales<br>Whop, Lisa; Australian National University, National Centre for Epidemiology and Population Health<br>Marshall, Helen; The University of Adelaide; The University of Adelaide
**Primary Subject Heading**:	Infectious diseases
Secondary Subject Heading:	Public health, Infectious diseases
Keywords:	INFECTIOUS DISEASES, Public health < INFECTIOUS DISEASES, Primary Prevention, COVID-19

TITLE PAGE

Nudging toward COVID-19 and influenza vaccination uptake in medically at risk children – EPIC study protocol of randomised controlled trials in Australian paediatric outpatient clinics

Bing Wang[1,2,3], Prabha H Andraweera[1,2,3], Margie Danchin[4,5], Christopher C Blyth[6,7,8], Ivo Vlaev[9], Jason J. Ong[10], Jodie Dodd[2,3,11], Jennifer Couper[2,3,12], Thomas R Sullivan[13,14], Jonathan Karnon[15], Nicola Spurrier[16,17], Michael Cusack[17], Dylan Mordaunt[16], Dimi Simatos[18], Gus Dekker[2,3,19], Samantha Carlson[6], Jane Tuckerman[4,5,], Nicholas Wood[20,21], Lisa Whop[22], Helen S Marshall[1,2,3]

[1] Vaccinology and Immunology Research Trials Unit, Women's and Children's Hospital, SA Health, Adelaide, South Australia

[2] Robinson Research Institute, The University of Adelaide, Adelaide, South Australia, Australia

[3] Adelaide Medical School, The University of Adelaide, Adelaide, South Australia, Australia

[4] Murdoch Childrens Research Institute, Melbourne, Victoria, Australia; The Royal Children's Hospital, Melbourne, Victoria, Australia

[5] Department of Paediatrics, The University of Melbourne, Melbourne, Australia

[6] Wesfarmers Centre of Vaccines and Infectious Diseases, Telethon Kids Institute and School of Medicine, University of Western Australia, Western Australia, Australia

[7] Department of Infectious Diseases, Perth Children's Hospital, Perth, Western Australia, Australia

[8] Department of Microbiology, PathWest Laboratory Medicine, Queen Elizabeth II Medical Centre (QEIIMC), Western Australia, Australia

For peer review only - http://bmjopen.bmj.com/site/about/guidelines.xhtml

[9] School of Business, Warwick University, Warwick, United Kingdom

[10] Central Clinical School, Monash University, Melbourne, Victoria, Australia

[11] Women's and Babies Division, Women's and Children's Hospital, Adelaide, South Australia, Australia

[12] Division of Paediatrics, Women's and Children's Hospital, Adelaide, South Australia, Australia

[13] South Australian Health and Medical Research Institute, South Australia, Australia

[14] School of Public Health, The University of Adelaide, Adelaide, South Australia, Australia

[15] Discipline of Public Health, Flinders University, South Australia, Australia

[16] Discipline of Paediatrics, Flinders University, South Australia, Australia

[17] SA Health, South Australian Government, Australia

[18] Discipline of Paediatrics, Lyell McEwin Hospital, South Australia, Australia

[19] Discipline of Women's Health, Lyell McEwin Hospital, South Australia, Australia

[20] Discipline of Paediatrics, University of Sydney, Sydney, New South Wales, Australia

[21] Children's Hospital Westmead, Sydney, New South Wales, Australia

[22] Discipline of Public Health, Australian National University, Canberra,

Key words: COVID-19, Influenza, vaccine, medically at risk children

Correspondence: Prof. Helen Marshall, Vaccinology and Immunology Research Trials Unit, Women's and Children's Hospital, Adelaide, South Australia, Australia; phone +61 (08) 8313 4086, fax +61 (08) 8313 4086, email helen.marshall@adelaide.edu.au

For peer review only - http://bmjopen.bmj.com/site/about/guidelines.xhtml

ABSTRACT

Introduction: Children with chronic medical diseases are at an unacceptable risk of hospitalisation and death from influenza and coronavirus 2 (SARS-CoV-2) infections. Over the past two decades, behavioural scientists have learnt how to design non-coercive "nudge" interventions to encourage positive health behaviours. Our study aims to evaluate the impact of multi-component nudge interventions on the uptake of coronavirus disease (COVID-19) and influenza vaccines in medically at risk children.

Methods and analyses: Two separate randomized controlled trials (RCTs), each with 1038 children, will enrol a total of approximately 2076 children with chronic medical conditions who are attending tertiary hospitals in South Australia, Western Australia, and Victoria. Participants will be randomly assigned (1:1) to the standard care or intervention group. The nudge intervention in each RCT will consist of three text message reminders with four behavioural nudges including (i) social norm messages, (ii) different messengers through links to short educational videos from a paediatrician, medically at risk child and parent, and nurse, (iii) a pledge to have their child or themselves vaccinated and (iv) information salience through links to the current guidelines and vaccine safety information. The primary outcome is the proportion of medically at risk children who receive at least one dose of vaccine within 3 months of randomisation. Logistic regression analysis will be performed to determine the effect of the intervention on the probability of vaccination uptake.

Ethics and dissemination: The protocol and study documents have been reviewed and approved by the Women's and Children's Health Network Human Research Ethics Committee (HREC/22/WCHN/2022/00082). Results will be published via peer-reviewed journals and presented at scientific meetings and public forums.

ClinicalTrials.gov Identifier: NCT05613751

For peer review only

For peer review only - http://bmjopen.bmj.com/site/about/guidelines.xhtml

Article Summary

Strengths and limitations of this study

- The Nudgeathon involved collaboration with various stakeholders who co-designed the nudge interventions.
- Nudge interventions are currently undergoing evaluation in randomized controlled trials, with an additional assessment of cost-effectiveness.
- The waiver of consent in our study helps mitigate selection bias.
- The general public has experienced multiple COVID-19 vaccine campaigns, which may lead to fatigue with any further COVID-19 promotion strategies.

For peer review only

For peer review only - http://bmjopen.bmj.com/site/about/guidelines.xhtml

INTRODUCTION

Children with chronic medical conditions represent a clinically vulnerable group in Australia with around 15% of children having either a respiratory, cardiac or neurological condition that increases their risk of hospitalisation and death from respiratory infections.[1] A systematic review and meta-analysis conducted by our group in 2017 demonstrates that compared with healthy peers, medically at risk children with influenza are at higher risks for intensive care unit (ICU) admission, mechanical ventilation and death.[2] They are also more likely to develop bacterial pneumonia or experience prolonged hospital length of stay.[2] Australian data on children ≤ 16 years admitted with acute respiratory illnesses and tested for influenza at sentinel hospitals between 2010 and 2019 demonstrate that comorbidities are an independent predictor of severe outcomes. Specifically, the odds of ICU admission are higher in those with any comorbidity (adjusted odds ratio (aOR): 1.36, 95% Confidence Interval [95% CI]: 1.05-1.77) compared with cases without any comorbidity. Children with diabetes (aOR: 3.22, 95% CI: 1.25; 8.23), cardiac (aOR: 1.93, 95% CI: 1.23-3.03), respiratory (aOR: 1.54, 95% CI: 1.08-2.21), or neurological comorbidities (aOR: 1.57, 95% CI: 1.25-1.98) are at greatest odds of ICU admission. Respiratory, neurological, cardiac, genetic, and hepatic comorbidities and diabetes were shown to be associated with prolonged hospitalisation, whereas respiratory, renal, cardiac, and other comorbidities increased the length of ICU stay.[3] The cost per episode of influenza-associated hospitalisation was estimated to be AUD $19,704 (95% CI: 11,715-27,693) for children with chronic lung conditions compared to AUD $4,557 (95% CI: 4,129-4,984) for children without.[4] Influenza vaccines are provided free to this group (≥ 6 months).[5] Our research has previously shown that parents of children with chronic medical conditions are less likely to have their children vaccinated unless recommended by their specialist.[6] The uptake of influenza vaccine at least once in the last two years among medically at risk children is 50% with annual vaccination only 32.8%.[7]

For peer review only

For peer review only - http://bmjopen.bmj.com/site/about/guidelines.xhtml

The coronavirus 2 (SARS-CoV-2) infection causing Coronavirus disease (COVID-19) may overall be milder in children compared to adults but a small proportion develop severe disease and are at risk of a debilitating multisystem-inflammatory syndrome (MIS-C) and/or long COVID (long lasting complications of COVID-19).[8] Children with chronic diseases are also at a higher risk of COVID-19 complications including pneumonia and respiratory failure and long-term health consequences.[4, 9] Long COVID is a multi-system illness characterised by ongoing persistent symptoms that can last for weeks or months following SARS-CoV-2 infection.[10] Chronic fatigue, short term memory problems, loss of taste and smell and long school absences were reported in long COVID cases.[11] COVID-19 vaccines are provided free to medically at risk children (≥ 6 months).[5] Of concern, two-dose COVID-19 vaccine uptake among children aged 5-11 years is at present approximately 40% nationally.[12] At a time when Australia is still experiencing a large number of SARS-CoV-2 infections, with greater risks to the individual and the health system of co-circulation of influenza and SARS-CoV-2 infections and emergence of new viral variants, it is critical that all efforts are made to improve vaccine uptake among these high-risk groups. Equally important is the opportunity to continue to evaluate the safety of COVID-19 vaccines in these groups including children with chronic conditions that are often excluded from clinical trials.

Lack of access and practical barriers to vaccines remain the leading cause of low vaccine uptake, which is especially relevant to patients whose care is primarily in a hospital setting. For children with chronic conditions the hospital often takes on the role of primary as well as tertiary care due to parental anxiety, life pressures, timeliness and access issues. Parents have identified barriers such as concern and lack of confidence in family physicians providing a vaccine recommendation for medically at risk children and access to vaccines in hospital

For peer review only

For peer review only - http://bmjopen.bmj.com/site/about/guidelines.xhtml

settings being sub-optimal. Mobility, transport, language, financial barriers, and remoteness are also reported barriers for children with chronic and complex conditions in accessing services.[13] For these groups, vaccination in the hospital setting, when patients are already onsite and engaged with a healthcare setting, would improve immunisation opportunities and protection against the threat of both influenza and SARS-CoV-2 infection.

Improving uptake of recommended vaccines in medically at risk children primarily attending hospitals requires an innovative rather than a population approach. Over the past two decades, behavioural scientists have learnt how to design non-coercive "nudge" strategies to promote positive behaviours in a range of contexts. In psychology and neuroscience, a dual process theory describes brain functioning as two types of cognitive processes - 'System 1' processes described as automatic, uncontrolled, effortless, associative, fast, unconscious and affective, and 'System 2' processes described as reflective, controlled, effortful, rule-based, slow, conscious and rational.[14] Although this 'dual process' model is considered as a theoretical basis for nudge theory, the nudge approach suggests that automatic decisions can be systematically triggered to improve health outcomes.[15] In other words, nudges are simple cues in our environment that influence people to behave in a certain way to achieve better personal or social goals without making a conscious decision to do so.[16] Using behavioural economics (a discipline that studies how individuals make choice) the environment in which a choice is being made can actively be designed to encourage better health-related choices.[17] A previous study showed nudges and reminders resulted in a decrease in energy-dense nutrient-poor foods in men and sugar-sweetened beverages in women, together with a reduction in body weight.[18] A "nudge" intervention needs to be simple and low cost and if proven successful can be simply incorporated into standard health care. For example, redesigning cardiac rehabilitation referral decision pathway from opt-in to opt-out referral, which automatically

For peer review only

For peer review only - http://bmjopen.bmj.com/site/about/guidelines.xhtml

identified eligible patients from the electronic health record and notified staff on the wards by using secure text messaging, increased cardiac rehabilitation referral rates from 15% to 85%.[19] Nudge-based interventions to address vaccine hesitancy include using reminders and recall, changing the way information is framed and delivered to an intended audience, changing the messenger delivering information, invoking social norms and emotional affect (e.g., through storytelling, dramatic narratives and graphical presentations), and offering incentives or changing defaults. Nudge-based interventions show potential to increase vaccine confidence and uptake. Interestingly, strategies with educational approaches are less effective (or not effective at all) in improving vaccine uptake.[20] The most promising evidence exists for nudges that offer incentives to parents and healthcare workers, that make information more salient, that frame vaccination as the default, or that use trusted messengers to deliver information.[21, 22] Normalizing vaccination and peer influence can activate social tendencies to join others.[23] Several studies have explored the impact of different interventions on vaccination rates and intentions. A large study with 57,893 participants was conducted in a Northern California health system, and found that personal reminder messages increased booster vaccination rates.[24] Among 964,870 participants in 691,820 households in Denmark, two strategies increased influenza vaccination rates.[25, 26] In the US, a study found that short video messages addressing specific COVID-19 vaccine concerns increased vaccination intentions.[27] In a March 2021 study with 1,595 participants in Japan, different messages were tested to encourage COVID-19 vaccination. The 'influence-gain' message was effective for older adults. 'Comparison' and 'influence-loss' messages reinforced existing intentions among older adults.[23] In our previous study conducted at a tertiary paediatric hospital in Adelaide (n=600), a significantly greater proportion receiving the SMS intervention were vaccinated with 38.6% in the SMS intervention group compared with 26.2% in the control group.[28] A recent study was conducted in older adult populations (n=48,125) and found behavioural

For peer review only - http://bmjopen.bmj.com/site/about/guidelines.xhtml

nudges, electronically delivered letters or centralized written reminders, significantly increased influenza vaccination uptake in Finland.[29] A randomized controlled trial (RCT) of a nudge intervention that included text message reminders demonstrated that the first reminder (n=93,354) increased appointment and COVID-19 vaccination rates by 6.07% and 3.57% and that the second reminder (n=67,092) increased those by 1.65% and 1.06% respectively in the early stages of the COVID-19 vaccine rollout.[30] Another RCT was conducted a few months later in a younger population (mean age 39 years; n = 142,428) and no SMS message did substantially better or worse than the control whether vaccination rates were measured one week after the messages were sent or at the end of the study period.[31] The difference between two studies may suggest that nudges help early in vaccination campaigns, but the efficacy decays. However, nudging-based interventions have shown potential to increase vaccine confidence and uptake in many studies, but further evidence is needed for the development and evaluation of clear recommendations.[22]

Our team recently conducted an RCT from April 15 to September 30, 2021 and found a 47% relative increase in uptake of influenza vaccine in medically at risk children using a simple SMS nudge co-designed with paediatricians.[28] If our text based nudges with links to short educational videos prove to be successful in improving influenza and COVID-19 vaccine uptake in medically at risk children, they can easily be implemented as a promising, valuable, low-cost and long-term tool at a national level.

However, a challenge is how behavioural economic concepts and principles can be effectively applied by local community stakeholders to identify barriers and create an innovative intervention to complement the current immunisation programs. To bridge this gap, a Nudgeathon,[32] a crowdsourcing interaction in which decision groups draw on insights and

For peer review only - http://bmjopen.bmj.com/site/about/guidelines.xhtml

methods from nudge theory, augmented by design thinking and drama theory to devise implementable solutions to major behavioural policy problems, was launched.[32] These events are typically conducted over 1-2 days, bringing together a diverse range of stakeholders (many who are new to behavioural science) to generate new approaches to complex problems in a time-pressured setting. Three dozen Nudgeathons [32] around the world have been conducted to address multiple issues (e.g. handwashing among health providers, increasing regular HIV/sexually transmitted infection testing in men [17, 33]) but to our knowledge, there has not been a Nudgeathon for the immunisation programs. The MINDSPACE framework, which provides a list of behaviour change techniques including Messenger, Incentives, Norms, Defaults, Salience, Priming, Affect, Commitments, Ego, that target the automatic decision processes,[34] was used to identify and generate potential nudges at the Nudgeathon. Our goal through the Nudgeathons is to gain valuable "Customer Insight" within the Australian context, fostering a profound understanding of parents' experiences, beliefs, needs, and desires, while also identifying the practical and structural challenges they encounter. It's important to recognise that attempts to promote behaviour change without considering these contextual factors often result in frustration. Behaviour change initiatives can be contentious, involving intricate trade-offs and often addressing areas where government decisions are controversial such as COVID-19 vaccination policies. Consequently, innovative approaches like Nudgeathons may be essential to engage the public effectively in exploring acceptable courses of action.

To our knowledge, this will be the first study assessing the effectiveness and cost-effectiveness of a co-designed multi-component nudge intervention on improving influenza and COVID-19 vaccine uptake in medically at-risk children. Vaccine hesitancy and access barriers are a major barrier that reduces the progress of management of vaccine preventable diseases in children.

For peer review only

For peer review only - http://bmjopen.bmj.com/site/about/guidelines.xhtml

Identifying solutions to improving COVID-19 and influenza vaccine uptake in medically at risk children is critical, as these children are more vulnerable to severe COVID-19 and influenza than healthy children.

Therefore, this study aims to design nudge interventions and evaluate the impact of multi-component nudge interventions on the uptake of influenza and COVID-19 vaccines among medically at risk children.

METHODS AND ANALYSIS

Recommendations for Intervention Trials (SPIRIT) checklist was used when developing this protocol.[35] The first participant was randomised on 29 November 2022 and the study is expected to be complete by 31 December 2023. The master protocol was devised to evaluate the impact of multi-component nudge interventions on the uptake of COVID-19 and influenza vaccines in two distinct sub-populations: medically at-risk children and pregnant women, employing a unified and overarching design. The information pertaining to medically at-risk children is presented here. The protocol information regarding the sub-study involving pregnant women was published elsewhere.[36]

Nudge intervention

In August 2022, a Nudgeathon was held to design appropriate nudges for inclusion in the trial. Fourteen participants including medically at risk children, their parents, paediatricians, nurses, hospital administrative personnel, behavioural scientists, psychologists and graphic designers with diverse skills from South Australia (SA), Western Australia and Victoria participated in the Nudgeathon. The participants learnt about behavioural science and successful application of nudges in different disciplines. They were assigned into small groups ensuring that each

For peer review only - http://bmjopen.bmj.com/site/about/guidelines.xhtml
For peer review only

group comprised participants with different skills. The individual groups were requested to create a nudge for improving either COVID-19 or influenza vaccine uptake in medically at risk children. At the end of the Nudgeathon each group presented their nudges and one nudge for each condition was chosen by the team to develop the nudge interventions in the following randomised control trials. Some of the most robust effects that have been found to have strong impacts on behaviour[34] such as Messenger, Norms, Salience and Commitments were used to generate nudge interventions in our study. The carefully chosen nudge for both COVID-19 and influenza vaccines consists of three SMS text messages that are sent to parents of medically at risk children from the participating hospitals four weeks apart. The first text message is sent one week prior to a scheduled paediatric clinic appointment and provides a link to current national guidelines on COVID-19 or influenza vaccination in children with chronic illness and a link to a video of a paediatrician explaining the benefits of COVID-19 or influenza vaccination in medically at risk children. The second text message provides a link to a video of a child with chronic illness and their parent giving their opinion on the protection of medically as risk children against SARS-CoV-2 or influenza infections. The third text message provides a link to a video of a nurse discussing the potential adverse outcomes of not being vaccinated against SARS-CoV-2 or influenza infections. The first and second text messages also provide the option for a parent to agree to vaccinate their children or opt out from receiving further reminders.

The study is aligned with the MINDSPACE Framework, a behavioural change model developed by the UK government's Behavioural Insights Team in 2010. The decision to incorporate a blend of behavioural nudges in the intervention stems from the notion that a multifaceted approach can potentially yield a more substantial impact on behaviour. Combining various nudges is often considered a strategy to enhance the likelihood of success,

For peer review only - http://bmjopen.bmj.com/site/about/guidelines.xhtml

as different individuals may respond diversely to different nudge effects.[37, 38] This approach seeks to cast a broader net to appeal to a more extensive spectrum of people and behaviours, leveraging the effects encapsulated in the acronym MINDSPACE, which includes Messenger, Incentives, Norms, Defaults, Salience, Priming, Affect, Commitment, and Ego. Significantly, there is a considerable overlap among these effects, and the most effective interventions will invariably integrate various elements.

Primary objectives

- To determine the proportion of medically as risk children in the intervention versus standard care (non-intervention) group receiving at least one dose of the seasonal influenza vaccine within 3 months after randomisation, as assessed using the Australian Immunisation Register (AIR)
- To determine the proportion of medically as risk children in the intervention versus standard care (non-intervention) group receiving at least one dose of a COVID-19 vaccine within 3 months after randomisation, as assessed using the AIR.

The AIR is a national register that records vaccines given to all people in Australia including vaccines given under the National Immunisation Program, including school programs, and privately, such as for influenza. A recognised vaccination provider including doctors, such as a family physician or community health centre, are required to update the AIR once an influenza, COVID-19 or any vaccine listed in the National Immunisation Program Schedule is administered.

Secondary objectives

For peer review only - http://bmjopen.bmj.com/site/about/guidelines.xhtml

- To identify socio-demographic characteristics and medical risk factors associated with COVID-19 or influenza vaccination in medically at risk children
- To assess timeliness of influenza and COVID-19 vaccine uptake among medically at risk children during the study period by determining the proportion of medically at risk children who receive the COVID-19 or influenza vaccine by month throughout the study period.
- To estimate the cost-effectiveness of proven interventions compared to standard care in hospital settings

Study design and study population

These are parallel-group randomised controlled trials (RCTs, ClinicalTrials.gov Identifier: NCT05613751) designed to measure the impact of a nudge intervention on receipt of one dose of the COVID-19 or influenza vaccine in medically at risk children who attend a clinic at tertiary hospitals in South Australia, Western Australia and Victoria. Medically at risk children will be enrolled at seven hospitals across South Australia (Women's and Children's Hospital, Flinders Medical Centre and the Lyell McEwin Hospital), Victoria (The Royal Children's Hospital) and Western Australia (Perth Children's Hospital). Each of these hospitals covers different socio-demographic areas and patient sources ensuring our research is generalisable. COVID-19 or influenza vaccination status of medically at risk children attending the participating hospitals will be determined by checking the AIR. The inclusion and exclusion criteria are listed in Table 1.

For peer review only - http://bmjopen.bmj.com/site/about/guidelines.xhtml

330 Table 1 Inclusion and exclusion criteria

	Exclusion criteria	Inclusion criteria
COVID vaccine [39]	• Known contraindications to COVID-19 vaccine<br>• Up to date for COVID-19 vaccine (≥ two doses) at the time of enrolment,<br>• Sibling of a child already enrolled in the trial (only the sibling who is eligible and scheduled to attend a paediatric clinic first will be eligible)<br>• Previous participation in the influenza nudge RCT	Medically at risk children aged 5 years to 18 years with a cardiac, endocrine, respiratory, gastrointestinal, haematological, musculoskeletal, neurological condition
Influenza vaccine [5]	• Known contraindications to influenza vaccine<br>• Already received an influenza vaccine during the flu season in 2023<br>• Sibling of a child already participating in the trial (the sibling who is eligible and scheduled to attend a paediatric clinic first will be eligible)	Children aged ≥6 months and < 18 years with medical conditions specified in this list: immunocompromising conditions including malignancy, chronic steroid use, haematopoietic stem cell transplant; functional or anatomical asplenia including sickle cell disease or other haemoglobinopathies, congenital or acquired asplenia (for example, splenectomy) or hyposplenia; cardiac disease including cyanotic congenital heart disease, congestive heart failure, coronary

For peer review only - http://bmjopen.bmj.com/site/about/guidelines.xhtml

	Exclusion criteria	Inclusion criteria
	• Previous participation in the COVID-19 nudge RCT	artery disease; chronic respiratory conditions including suppurative lung disease, bronchiectasis, cystic fibrosis, chronic obstructive pulmonary disease, severe asthma (requiring frequent medical consultations or the use of multiple medicines); chronic neurological conditions including hereditary and degenerative CNS diseases, seizure disorders, spinal cord injuries, neuromuscular disorders; chronic metabolic disorders including Type 1 or 2 diabetes, amino acid disorders, carbohydrate disorders, cholesterol biosynthesis disorders, fatty acid oxidation defects, lactic acidosis, mitochondrial disorders, organic acid disorders, urea cycle disorders, vitamin/cofactor disorders, porphyria; chronic renal failure; children aged 5 to 10 years receiving long term aspiring therapy; Down syndrome; obesity (body mass index ≥30 kg/m2); children born less than 37 weeks gestation

331

For peer review only - http://bmjopen.bmj.com/site/about/guidelines.xhtml

Randomisation

Medically at risk children will be randomised to the intervention and standard care groups in a 1:1 ratio. The randomisation list will be generated using R version 4.02 by an independent statistician who will not be involved with the conduct or analysis of the RCT. Allocations will be performed using randomly permuted blocks, stratified by hospital (i.e., separate randomisation sequence used in each hospital). Randomisation will be done on a password protected REDCap database securely held on the University of Adelaide server.

Endpoint measurements will involve low level contact of study staff, however when contact is required (such as AIR/ confirmation of influenza/COVID-19 vaccine receipt etc), this will be carried out by trial staff shielded from information that might reveal trial group assignment. The study statistician undertaking the analysis and study investigators will remain blinded to trial intervention assignment.

Study processes

Medically at risk children attending clinics at the hospitals will be identified from Outpatients' Department's appointment lists. Research nurses will assess COVID-19 or influenza vaccination status of children on the AIR to screen for eligibility. Medically at risk children will be randomised to the intervention arm or the standard care arm on REDCap. Demographic data will be obtained from hospital medical records. Using the Norms, Messenger, Salience and Commitment nudge strategies, parents of medically at risk children randomised to the intervention arm will receive a maximum of three SMS reminders from the hospital using 'Message Media' software from the hospitals. The SMS messages will comprise a brief message that many children with chronic illness obtain the COVID-19 or influenza vaccine (Norms) and a reminder for the child to obtain the vaccine. Parents of medically at risk children

For peer review only - http://bmjopen.bmj.com/site/about/guidelines.xhtml
For peer review only

have the option of responding to the message by 1) agreeing to obtain the vaccine (Commitment), 2) stating that the participant has already received the vaccine or 3) requesting to opt out from receiving further reminders (first and second SMS messages). Each SMS message also provides a link to a video of (Messager): 1) a paediatrician discussing the potential serious health consequences of SARS-CoV-2 or influenza infections (first SMS), 2) a medically at risk child and their parent stating that the child received the vaccine and that the child is protected from serious adverse effects of SARS-CoV-2 or influenza infections (second SMS), 3) a nurse stating the benefits of COVID-19 or influenza vaccination for medically at risk children (third SMS). In addition, the first SMS also states children with special medical conditions are at increased risk of severe COVID-19 or Influenza disease and provides a link to the current guidelines and safety information about COVID-19 or influenza vaccination (Salience). The paediatrician and nurse assumed the roles of healthcare experts, while the child with medical risks and their parent acted as relatable peers with a similar background. They served as influential messengers in persuading parents to vaccinate their children. The message highlighting the fact that many children with chronic illnesses receive the COVID-19 or influenza vaccine utilised a social norms approach, conveying what other parents in similar situations typically do. Meanwhile, the message emphasising the increased risk of severe COVID-19 or influenza for children with special medical conditions was designed to capture parents' attention, as it is information more likely to be comprehensible and directly relevant to their own children. The response options were strategically crafted to foster reciprocity and serve as commitments. The first SMS is sent approximately one week prior to a scheduled clinic visit. The second SMS message is sent four weeks after the first message to those who have not received a dose of COVID-19 or influenza vaccine after the first SMS (confirmed on AIR) and not requested to opt out. The third SMS message is sent four weeks after the second message to those who have not received a dose of COVID-19 or influenza vaccine after the

For peer review only - http://bmjopen.bmj.com/site/about/guidelines.xhtml

second SMS (confirmed on AIR) and not requested to opt out. The AIR will be checked at 3 months after randomisation to assess whether medically at risk children have received a dose of COVID-19 or influenza vaccine. All data will be stored securely on a password protected REDCap database held by the University of Adelaide. All data will be de-identified prior to presentation and publication.

Study monitoring and surveillance

The nudge is a behavioural change intervention and hence there are no risks related to invasive procedures or investigational medications. Any risks of mental distress associated with receiving the text message is expected to be very low and the parents have the option of opting out from receiving the further text messages after the first one. However, a risk assessment and management plan has been developed for this study. Moreover, the study management committee comprising the chief investigator, site investigators, study coordinators and statistician will carefully monitor the progress of the study.

This trial has certain limitations. The nudge interventions incorporated a blend of various nudge techniques, and we did not assess the individual effects of these techniques in our study. We included links within the text messages, and it was the parents' responsibility to click on these links. If the links were not clicked, the nudge interventions may not have been as effectively implemented as originally intended.

Sample size and statistical analysis plan

To detect an increase in the vaccination rate from ~50% in the control group to 60% in the intervention group, which is considered a clinically relevant impact, with 90% power (two-sided test with alpha = 0.05), a sample size of n = 519 per group (1038 total) is required. Should

For peer review only - http://bmjopen.bmj.com/site/about/guidelines.xhtml

the vaccination rate in the control arm be higher than 50% or lower than 40%, this sample size will still provide at least 90% power to detect a 10% absolute increase in vaccination with the nudge intervention. We aim to enrol at least 1038 medically at risk children to each of the two RCTs across the participating hospitals.

Statistical analyses will be conducted on an Intention To Treat basis according to a pre-specified statistical analysis plan. Baseline characteristics including age, socio-economic status based on postcode, and ethnicity will be reported for each group using percentages, means with standard deviations or medians with ranges as appropriate. The vaccination rate will be compared between intervention and control groups using logistic regression, with adjustment only made for participating hospitals (treated as a categorical fixed effect). Intervention effects will be reported as odds ratios with 95% confidence intervals. Patient characteristics associated with vaccination will be investigated using multivariable logistic regression models. Cost-effectiveness analysis of the nudge intervention will be compared to standard care from the healthcare payer perspective. The primary cost-effectiveness analysis will estimate the incremental cost per additional person vaccinated, in each of the two RCTs. Secondary analyses for the trial focused on influenza vaccination will estimate the cost per quality-adjusted life year (QALY) gained. The study results will be used to estimate the impact of the nudge intervention on health-related quality of life by calculating the QALYs gained as a result of the increased vaccine uptake. Implementation costs will be obtained from the study budget and costs related to research activities will be excluded. Estimated cost offsets to the health system associated with influenza related disease (e.g. hospitalisations and emergency visits) will be derived from the literature and calculated using cost weights for Australian Refined Diagnosis Related Groups (AR-DRGs). The cost-effectiveness evaluation will follow standard reporting guidelines in the CHEERS statement.[40]

For peer review only

For peer review only - http://bmjopen.bmj.com/site/about/guidelines.xhtml

ETHICS AND DISSEMINATION

Stakeholders including medically at risk children and their parents participated in the Nudgeathon to develop the nudge intervention. The current protocol version 2.0 (05-01-2023) and all study material have been reviewed and approved by the Women's and Children's Health Network Human Research Ethics Committee (HREC/2022/00082) and research governance approval has been obtained from the Women's and Children's Hospital, Flinders Medical Centre, and Lyell McEwin Hospital in SA, The Royal Children's Hospital in VIC, and Perth Children's Hospital in Western Australia. The written consent was obtained from all stakeholders including health service leaders and clinicians and health consumers to participate in the Nudgeathons. A waiver of consent was approved for medically at risk children to participate in this trial and receive SMS messages in accordance with the National Statement on Ethical Conduct in Human Research. Three criteria guide this. Firstly, there are no estimated risks or harms associated with study procedures. Any risk of psychosocial distress associated with receiving the nudge interventions is very unlikely. Secondly, it would not be possible to obtain informed consent without threatening the validity of the trials. It would be impossible to assess the effectiveness of the nudge by recruiting parents of medically at risk children and informing them about the study and then randomising them not to receive the nudge. Thirdly, as influenza and COVID-19 vaccines are recommended for all medically at risk children aged ≥ 6 months, all data for the influenza and COVID-19 RCTs will be collected as part of routine care, including patient demographics and specialties seen. In addition this methodology was approved and successfully applied in the Flutext-4U study.[41] We identified a significant difference in uptake of influenza vaccine in medically at risk children in the Flutext-4U study, likely due to this being a population inclusive study unaffected by healthy selection bias.[28] The study is being conducted in accordance with the Declaration of Helsinki and the

For peer review only - http://bmjopen.bmj.com/site/about/guidelines.xhtml

International Conference on Harmonization Guidelines on Good Clinical Practice. The trial is being conducted in compliance with the current version of the protocol. Any change to the protocol that affects scientific content, study design, and participating will be considered an amendment and will be submitted to HREC for review and approval prior to implementation.

Participant confidentiality is strictly held in trust by the investigators. Therefore, study documentation, data, and all other information generated will be held in strict confidence. No information concerning the study, or the data will be released to any unauthorised third party. Study participant research data, which is for purposes of statistical analysis and scientific reporting, will be transmitted to and stored at the Women's and Children's Hospital. At the end of the study, all study databases will be de-identified and archived at the University of Adelaide. After the completion of the study, the de-identified and aggregated results will be presented at scientific forums and submitted for publication in peer-reviewed journals. The results will be disseminated regardless of the direction of effect. The findings of the study will be communicated to key stakeholders.

Medically at risk children and their parents actively contributed to the study's design during the Nudgeathon. Their insights played a crucial role in designing the nudge interventions, making it more relevant and patient-centred. The study results will be disseminated to the trial participants, which includes medically at risk children and their parents, through various accessible means, such as academic publication and lay summaries on our research unit's webpage, to ensure that the findings are easily understood and meaningful to this group. The burden of the intervention is planned to be assessed with input from medically at risk children's parents using a short evaluation questionnaire. Special recognition and appreciation were

For peer review only

481 extended to the medical at risk children and their parents who participated in the Nudgeathon to highlight their valuable contributions to the study's design and success.

For peer review only

For peer review only - http://bmjopen.bmj.com/site/about/guidelines.xhtml

AUTHORS CONTRIBUTIONS

HM, IV, JO, MD, CB, BW, PA, JD, JC, TS, JK, NS, MC, DM, DS, GD, SC, JT, NW and LW conceptualised the interventions, contributed to the design of the study protocol, and acquired funding. TS provided the statistical consult. BW drafted the manuscript. MD, CB, JD, JC, JK, NS, MC, DM, DS, GD, SC, NW and LW critically reviewed. HM, BW, IV, JO, TS, JT and PA critically reviewed and edited the manuscript. HM, BW, IV, JO, MD, CB, PA, JD, JC, TS, JK, NS, MC, DM, DS, GD, SC, JT, NW and LW approved its final version.

ACKNOWLEDGEMENTS

We acknowledge the following study coordinators for their contribution to study set up and recruitment: Kirsty Herewane (Women's and Children's Hospital), Joanne Koch (Lyell McEwin Hospital), Lauren Thompson (Flinders Medical Centre), Ashleigh Rak (Royal Children's Hospital), Deborah Pidd (Mercy Hospital for Women), Louisa Paparo, Rebecca Pavlos and Erin Van Der Helder (Perth Children's Hospital).

FUNDING STATEMENT

This work is supported by the National Health and medical Research Council of Australia [NHMRC Partnership grant APP2014684]. Additional in-kind funding is provided by funding partners including the Department of Health, South Australia Government; Department of Health, Victorian Government; Department of Health, Western Australian Government; Women's and Children's Health Network; Southern Adelaide Local Health Network; Northern Adelaide Local Health Network; Women's and Children's Hospital Foundation; Department of Trade and Investment, South Australian Government, in support of the NHMRC Partnership grant.

For peer review only

For peer review only - http://bmjopen.bmj.com/site/about/guidelines.xhtml

COMPETING INTERESTS STATEMENT

HM acknowledges support from the National Health and Medical Research Council of Australia: Practitioner Fellowship (APP1155066). CCB acknowledges support from the National Health and Medical Research Council of Australia: Investigator Grant (APP1173163). HM is an independent investigator on clinical trials of investigational vaccines manufactured by pharmaceutical companies including Pfizer, ILiAD Biotechnologies, and Merck. The institution at which HM, BW and PA are employed has received funding for investigator-led research from GlaxoSmithKline, Sanofi-Pasteur, and Pfizer Vaccines. All authors receive no personal payments from industry. There are no other conflicts of interest to declare.

For peer review only

For peer review only - http://bmjopen.bmj.com/site/about/guidelines.xhtml

REFERENCES

[1] Australian Institute of Health and Welfare 2020. Australia's children. Cat. no. CWS 69. Canberra: AIHW. https://www.aihw.gov.au/reports/children-youth/australias-children/contents/executive-summary. Australia's Children. DOI: 10.25816/5ebca4d0fa7dd

[2] Tuckerman J, Misan S, Crawford NW, Marshall HS. Influenza in Children With Special Risk Medical Conditions: A Systematic Review and Meta-analysis. The Pediatric infectious disease journal. 2019;38:912-9.

[3] Norman DA, Cheng AC, Macartney KK, Moore HC, Danchin M, Seale H, et al. Influenza hospitalizations in Australian children 2010-2019: The impact of medical comorbidities on outcomes, vaccine coverage, and effectiveness. Influenza and other respiratory viruses. 2022;16:316-27.

[4] Homaira N, Briggs N, Oei JL, Hilder L, Bajuk B, Snelling T, et al. Impact of influenza on hospitalization rates in children with a range of chronic lung diseases. Influenza and other respiratory viruses. 2019;13:233-9.

[5] Australian Technical Advisory Group on Immunisation (ATAGI). Australian Immunisation Handbook, Australian Government Department of Health and Aged Care, Canberra, 2022, immunisationhandbook.health.gov.au

[6] Tuckerman J, Crawford NW, Marshall HS. Disparities in parental awareness of children's seasonal influenza vaccination recommendations and influencers of vaccination. PloS one. 2020;15:e0230425.

[7] Tuckerman J, Misan S, Salih S, Joseph Xavier B, Crawford NW, Lynch J, et al. Influenza vaccination: Uptake and associations in a cross-sectional study of children with special risk medical conditions. Vaccine. 2018;36:8138-47.

[8] Sudre CH, Murray B, Varsavsky T, Graham MS, Penfold RS, Bowyer RC, et al. Attributes and predictors of long COVID. Nature medicine. 2021;27:626-31.

For peer review only - http://bmjopen.bmj.com/site/about/guidelines.xhtml

[9] Moeller A, Thanikkel L, Duijts L, Gaillard EA, Garcia-Marcos L, Kantar A, et al. COVID-19 in children with underlying chronic respiratory diseases: survey results from 174 centres. ERJ open research. 2020;6.

[10] Australian Institute of Health and Welfare (2022) Long COVID in Australia – a review of the literature, catalogue number PHE 318, AIHW, Australian Government.

[11] Ludvigsson JF. Case report and systematic review suggest that children may experience similar long-term effects to adults after clinical COVID-19. Acta paediatrica (Oslo, Norway : 1992). 2021;110:914-21.

[12] https://www.health.gov.au/sites/default/files/2023-03/covid-19-vaccine-rollout-update-3-march-2023.pdf. COVID-19 Vaccine Roll-out (02 March 2023).

[13] Pearce A, Marshall H, Bedford H, Lynch J. Barriers to childhood immunisation: Findings from the Longitudinal Study of Australian Children. Vaccine. 2015;33:3377-83.

[14] Vlaev I, King D, Dolan P, Darzi A. The Theory and Practice of "Nudging": Changing Health Behaviors. Public Administration Review. 2016;76:550-61.

[15] Marteau TM, Ogilvie D, Roland M, Suhrcke M, Kelly MP. Judging nudging: can nudging improve population health? BMJ (Clinical research ed). 2011;342:d228.

[16] Simon C, Tagliabue M. Feeding the behavioral revolution: Contributions of behavior analysis to nudging and vice versa. Journal of Behavioral Economics for Policy. 2018;2:91-7.

[17] Ong JJ, Chow EPF, Read D, Taj U, Lee D, Vlaev I. Nudgeathons to control HIV: designing strategies using behavioural economics. AIDS. 2020;34:2337-40.

[18] Kerr DA, Harray AJ, Pollard CM, Dhaliwal SS, Delp EJ, Howat PA, et al. The connecting health and technology study: a 6-month randomized controlled trial to improve nutrition behaviours using a mobile food record and text messaging support in young adults. Int J Behav Nutr Phys Act. 2016;13:52.

For peer review only

For peer review only - http://bmjopen.bmj.com/site/about/guidelines.xhtml

[19] Adusumalli S, Jolly E, Chokshi NP, Gitelman Y, Rareshide CAL, Kolansky DM, et al. Referral Rates for Cardiac Rehabilitation Among Eligible Inpatients After Implementation of a Default Opt-Out Decision Pathway in the Electronic Medical Record. JAMA network open. 2021;4:e2033472.

[20] Dubé E, Gagnon D, MacDonald NE. Strategies intended to address vaccine hesitancy: Review of published reviews. Vaccine. 2015;33:4191-203.

[21] Tentori K, Pighin S, Giovanazzi G, Grignolio A, Timberlake B, Ferro A. Nudging COVID-19 Vaccine Uptake by Changing the Default: A Randomized Controlled Trial. Med Decis Making. 2022;42:837-41.

[22] Renosa MDC, Landicho J, Wachinger J, Dalglish SL, Barnighausen K, Barnighausen T, et al. Nudging toward vaccination: a systematic review. BMJ Glob Health. 2021;6.

[23] Sasaki S, Saito T, Ohtake F. Nudges for COVID-19 voluntary vaccination: How to explain peer information? Soc Sci Med. 2022;292:114561.

[24] Chang TY, Jacobson M, Shah M, Kopetsky M, Pramanik R, Shah SB. Reminders, but not monetary incentives, increase COVID-19 booster uptake. Proc Natl Acad Sci U S A. 2023;120:e2302725120.

[25] Johansen ND, Vaduganathan M, Bhatt AS, Lee SG, Modin D, Claggett BL, et al. Electronic nudges to increase influenza vaccination uptake in Denmark: a nationwide, pragmatic, registry-based, randomised implementation trial. Lancet. 2023;401:1103-14.

[26] Johansen ND, Vaduganathan M, Bhatt AS, Lee SG, Modin D, Claggett BL, et al. Nationwide Utilization of Danish Government Electronic Letter System for Increasing InFLUenza Vaccine Uptake (NUDGE-FLU): Study Protocol for a Nationwide Randomized Implementation Trial. Am Heart J. 2023.

[27] Jensen UT, Ayers S, Koskan AM. Video-based messages to reduce COVID-19 vaccine hesitancy and nudge vaccination intentions. PloS one. 2022;17:e0265736.

For peer review only

For peer review only - http://bmjopen.bmj.com/site/about/guidelines.xhtml

[28] Tuckerman J, Harper K, Sullivan TR, Cuthbert AR, Fereday J, Couper J, et al. Short Message Service Reminder Nudge for Parents and Influenza Vaccination Uptake in Children and Adolescents With Special Risk Medical Conditions: The Flutext-4U Randomized Clinical Trial. JAMA pediatrics. 2023.

[29] Saaksvuori L, Betsch C, Nohynek H, Salo H, Sivela J, Bohm R. Information nudges for influenza vaccination: Evidence from a large-scale cluster-randomized controlled trial in Finland. PLoS Med. 2022;19:e1003919.

[30] Dai H, Saccardo S, Han MA, Roh L, Raja N, Vangala S, et al. Behavioural nudges increase COVID-19 vaccinations. Nature. 2021;597:404-9.

[31] Rabb N, Swindal M, Glick D, Bowers J, Tomasulo A, Oyelami Z, et al. Evidence from a statewide vaccination RCT shows the limits of nudges. Nature. 2022;604:E1-E7.

[32] Nudgeathon. [cited 27 March 2023]. Available from: https://www.nudgeathon.co.uk..

[33] Tran J, Vlaev I, Read D, Schmidtke KA, Chow EP, Lee D, et al. A qualitative evaluation of a Nudgeathon event for the co-design of sexual health campaign images targeting overseas-born men who have sex with men. Sexual Health. 2023.

[34] Dolan P, Hallsworth M, Halpern D, King D, Vlaev I. MINDSPACE: influencing behaviour for public policy. 2010.

[35] Chan AW, Tetzlaff JM, Gotzsche PC, Altman DG, Mann H, Berlin JA, et al. SPIRIT 2013 explanation and elaboration: guidance for protocols of clinical trials. BMJ (Clinical research ed). 2013;346:e7586.

[36] Andraweera PH, Wang B, Danchin M, Blyth C, Vlaev I, Ong J, et al. Randomised controlled trials of behavioural nudges delivered through text messages to increase influenza and COVID-19 vaccines among pregnant women (the EPIC study): study protocol. Trials. 2023;24:454.

For peer review only

For peer review only - http://bmjopen.bmj.com/site/about/guidelines.xhtml

[37] Howley P, Ocean N. Can nudging only get you so far? Testing for nudge combination effects. European Review of Agricultural Economics. 2022;49:1086-112.

[38] Cosic A, Cosic H, Ille S. Can nudges affect students' green behaviour? A field experiment. Journal of Behavioral Economics for Policy. 2018;2:107-11.

[39] Australian Technical Advisory Group on Immunisation (ATAGI). Australian Immunisation Handbook, Australian Government Department of Health and Aged Care, Canberra, 2022, https://www.health.gov.au/initiatives-and-programs/covid-19-vaccines/advice-for-providers/clinical-guidance/clinical-features.

[40] Husereau D, Drummond M, Augustovski F, de Bekker-Grob E, Briggs AH, Carswell C, et al. Consolidated Health Economic Evaluation Reporting Standards 2022 (CHEERS 2022) Statement: Updated Reporting Guidance for Health Economic Evaluations. Value Health. 2022;25:3-9.

[41] Tuckerman J, Harper K, Sullivan TR, Fereday J, Couper J, Smith N, et al. Using provider-parent strategies to improve influenza vaccination in children and adolescents with special risk medical conditions: a randomised controlled trial protocol. BMJ open. 2022;12:e053838.

For peer review only

For peer review only - http://bmjopen.bmj.com/site/about/guidelines.xhtml

STANDARD PROTOCOL ITEMS: RECOMMENDATIONS FOR INTERVENTIONAL TRIALS

SPIRIT 2013 Checklist: Recommended items to address in a clinical trial protocol and related documents*

Section/item	Item No	Description	Page and Line Number	Reason if not applicable
**Administrative information**				
Title	1	Descriptive title identifying the study design, population, interventions, and, if applicable, trial acronym	P1, L3	
Trial registration	2a	Trial identifier and registry name. If not yet registered, name of intended registry	P3, L73	
	2b	All items from the World Health Organization Trial Registration Data Set	NA	Registered at clinicaltrials.gov.
Protocol version	3	Date and version identifier	P21, L435	
Funding	4	Sources and types of financial, material, and other support	P23, L497-505	
Roles and responsibilities	5a	Names, affiliations, and roles of protocol contributors	P23, L482-488	
	5b	Name and contact information for the trial sponsor	NA	Investigator led study
	5c	Role of study sponsor and funders, if any, in study design; collection, management, analysis, and interpretation of data; writing of the report; and the decision to submit the report for publication, including whether they will have ultimate authority over any of these activities	NA	Investigator led study
	5d	Composition, roles, and responsibilities of the coordinating centre, steering committee, endpoint adjudication committee, data management team, and other individuals or groups overseeing the trial, if applicable (see Item 21a for data monitoring committee)	P19, L388-395	
**Introduction**				

For peer review only - http://bmjopen.bmj.com/site/about/guidelines.xhtml

Background and rationale	6a	Description of research question and justification for undertaking the trial, including summary of relevant studies (published and unpublished) examining benefits and harms for each intervention	P5-11, L85-241	
	6b	Explanation for choice of comparators	P7-8, L172-182	
Objectives	7	Specific objectives or hypotheses	P10-11, L241-264	
Trial design	8	Description of trial design including type of trial (eg, parallel group, crossover, factorial, single group), allocation ratio, and framework (eg, superiority, equivalence, noninferiority, exploratory)	P13-14, L293-329	
**Methods: Participants, interventions, and outcomes**				
Study setting	9	Description of study settings (eg, community clinic, academic hospital) and list of countries where data will be collected. Reference to where list of study sites can be obtained	P14, L319-329	
Eligibility criteria	10	Inclusion and exclusion criteria for participants. If applicable, eligibility criteria for study centres and individuals who will perform the interventions (eg, surgeons, psychotherapists)	P15-16, Table 1	
Interventions	11a	Interventions for each group with sufficient detail to allow replication, including how and when they will be administered	P17-18, L347-386	
	11b	Criteria for discontinuing or modifying allocated interventions for a given trial participant (eg, drug dose change in response to harms, participant request, or improving/worsening disease)	P19, L389-401	
	11c	Strategies to improve adherence to intervention protocols, and any procedures for monitoring adherence (eg, drug tablet return, laboratory tests)	P19, L389-401	
	11d	Relevant concomitant care and interventions that are permitted or prohibited during the trial	NA	Normal care as the intervention is a text message

For peer review only - http://bmjopen.bmj.com/site/about/guidelines.xhtml

Outcomes	12	Primary, secondary, and other outcomes, including the specific measurement variable (eg, systolic blood pressure), analysis metric (eg, change from baseline, final value, time to event), method of aggregation (eg, median, proportion), and time point for each outcome. Explanation of the clinical relevance of chosen efficacy and harm outcomes is strongly recommended	P20, L412-431
Participant timeline	13	Time schedule of enrolment, interventions (including any run-ins and washouts), assessments, and visits for participants. A schematic diagram is highly recommended (see Figure)	P11 L245-246; L254-258
Sample size	14	Estimated number of participants needed to achieve study objectives and how it was determined, including clinical and statistical assumptions supporting any sample size calculations	P19-22, L404-410
Recruitment	15	Strategies for achieving adequate participant enrolment to reach target sample size	P21-22, L442-460

Methods: Assignment of interventions (for controlled trials)

Allocation:

Sequence generation	16a	Method of generating the allocation sequence (eg, computer-generated random numbers), and list of any factors for stratification. To reduce predictability of a random sequence, details of any planned restriction (eg, blocking) should be provided in a separate document that is unavailable to those who enrol participants or assign interventions	P17, L333-338
Allocation concealmen t mechanism	16b	Mechanism of implementing the allocation sequence (eg, central telephone; sequentially numbered, opaque, sealed envelopes), describing any steps to conceal the sequence until interventions are assigned	P17, L333-338
Implementat ion	16c	Who will generate the allocation sequence, who will enrol participants, and who will assign participants to interventions	P17, L333-338

For peer review only - http://bmjopen.bmj.com/site/about/guidelines.xhtml

Blinding (masking)	17a	Who will be blinded after assignment to interventions (eg, trial participants, care providers, outcome assessors, data analysts), and how	P17, L333-338
	17b	If blinded, circumstances under which unblinding is permissible, and procedure for revealing a participant's allocated intervention during the trial	P17, L333-338
**Methods: Data collection, management, and analysis**			
Data collection methods	18a	Plans for assessment and collection of outcome, baseline, and other trial data, including any related processes to promote data quality (eg, duplicate measurements, training of assessors) and a description of study instruments (eg, questionnaires, laboratory tests) along with their reliability and validity, if known. Reference to where data collection forms can be found, if not in the protocol	P17, L347-351<br>P19, L382-386
	18b	Plans to promote participant retention and complete follow-up, including list of any outcome data to be collected for participants who discontinue or deviate from intervention protocols	P21-22, L442-460
Data management	19	Plans for data entry, coding, security, and storage, including any related processes to promote data quality (eg, double data entry; range checks for data values). Reference to where details of data management procedures can be found, if not in the protocol	P19, L382-386<br>P22, L462-471
Statistical methods	20a	Statistical methods for analysing primary and secondary outcomes. Reference to where other details of the statistical analysis plan can be found, if not in the protocol	P20, L412-431
	20b	Methods for any additional analyses (eg, subgroup and adjusted analyses)	P20, L416-417
	20c	Definition of analysis population relating to protocol non-adherence (eg, as randomised analysis), and any statistical methods to handle missing data (eg, multiple imputation)	P20, L412
**Methods: Monitoring**			

For peer review only - http://bmjopen.bmj.com/site/about/guidelines.xhtml

Data monitoring	21a	Composition of data monitoring committee (DMC); summary of its role and reporting structure; statement of whether it is independent from the sponsor and competing interests; and reference to where further details about its charter can be found, if not in the protocol. Alternatively, an explanation of why a DMC is not needed	P19, 393-395
	21b	Description of any interim analyses and stopping guidelines, including who will have access to these interim results and make the final decision to terminate the trial	NA
Harms	22	Plans for collecting, assessing, reporting, and managing solicited and spontaneously reported adverse events and other unintended effects of trial interventions or trial conduct	P19, L390-393
Auditing	23	Frequency and procedures for auditing trial conduct, if any, and whether the process will be independent from investigators and the sponsor	P19, 393-395
**Ethics and dissemination**			
Research ethics approval	24	Plans for seeking research ethics committee/institutional review board (REC/IRB) approval	P21, L435-440
Protocol amendments	25	Plans for communicating important protocol modifications (eg, changes to eligibility criteria, outcomes, analyses) to relevant parties (eg, investigators, REC/IRBs, trial participants, trial registries, journals, regulators)	P22, L458-460
Consent or assent	26a	Who will obtain informed consent or assent from potential trial participants or authorised surrogates, and how (see Item 32)	P21, L440-455
	26b	Additional consent provisions for collection and use of participant data and biological specimens in ancillary studies, if applicable	NA

view only

For peer review only - http://bmjopen.bmj.com/site/about/guidelines.xhtml

Confidentiality	27	How personal information about potential and enrolled participants will be collected, shared, and maintained in order to protect confidentiality before, during, and after the trial	P22, L462-471	
Declaration of interests	28	Financial and other competing interests for principal investigators for the overall trial and each study site	P25, L509-517	
Access to data	29	Statement of who will have access to the final trial dataset, and disclosure of contractual agreements that limit such access for investigators	P22, L462-471	
Ancillary and post-trial care	30	Provisions, if any, for ancillary and post-trial care, and for compensation to those who suffer harm from trial participation	NA	
Dissemination policy	31a	Plans for investigators and sponsor to communicate trial results to participants, healthcare professionals, the public, and other relevant groups (eg, via publication, reporting in results databases, or other data sharing arrangements), including any publication restrictions	P22-23, 473-482	
	31b	Authorship eligibility guidelines and any intended use of professional writers	P24, L484-489	
	31c	Plans, if any, for granting public access to the full protocol, participant-level dataset, and statistical code	P22, L467-471	
**Appendices**				
Informed consent materials	32	Model consent form and other related documentation given to participants and authorised surrogates	NA	A consent waiver was approved by ethics committee
Biological specimens	33	Plans for collection, laboratory evaluation, and storage of biological specimens for genetic or molecular analysis in the current trial and for future use in ancillary studies, if applicable	NA	No biological samples are collected.

*It is strongly recommended that this checklist be read in conjunction with the SPIRIT 2013 Explanation & Elaboration for important clarification on the items. Amendments to the protocol should be tracked and dated. The SPIRIT checklist is copyrighted by the SPIRIT Group under the Creative Commons "Attribution-NonCommercial-NoDerivs 3.0 Unported" license.

For peer review only - http://bmjopen.bmj.com/site/about/guidelines.xhtml

Nudging toward COVID-19 and influenza vaccination uptake in medically at risk children – EPIC study protocol of randomised controlled trials in Australian paediatric outpatient clinics

Journal:	*BMJ Open*
Manuscript ID	bmjopen-2023-076194.R2
Article Type:	Protocol
Date Submitted by the Author:	29-Nov-2023

Complete List of Authors:	Wang, Bing; The University of Adelaide, Robinson Research Institute and Adelaide Medical School<br>Andraweera, Prabha ; University of Adelaide School of Medicine, Obstetrics and Gynaecology<br>Danchin, Margaret; Murdoch Childrens Research Institute, Paediatrics<br>Blyth, Christopher C.; The University of Western Australia<br>Vlaev, Ivo; Warwick Business School<br>Ong, Jason ; Monash University, Melbourne Sexual Health Clinic & LSHTM<br>Dodd, Jodie; The University of Adelaide, Obstetrics and Gynaecology<br>Couper, Jennifer; The University of Adelaide<br>Sullivan, Thomas; South Australian Health and Medical Research Institute<br>Karnon, Jonathan; Flinders University, CMPH<br>Spurrier, Nicola; SA Health Department for Health and Wellbeing<br>Cusack, Michael; SA Health Department for Health and Wellbeing<br>Mordaunt, Dylan; Flinders University<br>Simatos, Dimi; Discipline of Paediatrics Lyell McEwin Hospital<br>Dekker, Gustaaf; The University of Adelaide<br>Carlson, Samantha; Telethon Kids Institute, Wesfarmers Centre of Vaccines and Infectious Diseases; The University of Western Australia, School of Social Sciences<br>Tuckerman, Jane; Murdoch Childrens Research Institute, Infection and Immunity<br>Wood, Nicholas; University of New South Wales<br>Whop, Lisa; Australian National University, National Centre for Epidemiology and Population Health<br>Marshall, Helen; The University of Adelaide; The University of Adelaide

**Primary Subject Heading**:	Infectious diseases
Secondary Subject Heading:	Public health, Infectious diseases
Keywords:	INFECTIOUS DISEASES, Public health < INFECTIOUS DISEASES, Primary Prevention, COVID-19

SCHOLARONE™
Manuscripts

TITLE PAGE

Nudging toward COVID-19 and influenza vaccination uptake in medically at risk children – EPIC study protocol of randomised controlled trials in Australian paediatric outpatient clinics

Bing Wang[1,2,3], Prabha H Andraweera[1,2,3], Margie Danchin[4,5], Christopher C Blyth[6,7,8], Ivo Vlaev[9], Jason J. Ong[10], Jodie Dodd[2,3,11], Jennifer Couper[2,3,12], Thomas R Sullivan[13,14], Jonathan Karnon[15], Nicola Spurrier[16,17], Michael Cusack[17], Dylan Mordaunt[16], Dimi Simatos[18], Gus Dekker[2,3,19], Samantha Carlson[6], Jane Tuckerman[4,5,], Nicholas Wood[20,21], Lisa Whop[22], Helen S Marshall[1,2,3]

[1] Vaccinology and Immunology Research Trials Unit, Women's and Children's Hospital, SA Health, Adelaide, South Australia

[2] Robinson Research Institute, The University of Adelaide, Adelaide, South Australia, Australia

[3] Adelaide Medical School, The University of Adelaide, Adelaide, South Australia, Australia

[4] Murdoch Childrens Research Institute, Melbourne, Victoria, Australia; The Royal Children's Hospital, Melbourne, Victoria, Australia

[5] Department of Paediatrics, The University of Melbourne, Melbourne, Australia

[6] Wesfarmers Centre of Vaccines and Infectious Diseases, Telethon Kids Institute and School of Medicine, University of Western Australia, Western Australia, Australia

[7] Department of Infectious Diseases, Perth Children's Hospital, Perth, Western Australia, Australia

[8] Department of Microbiology, PathWest Laboratory Medicine, Queen Elizabeth II Medical Centre (QEIIMC), Western Australia, Australia

For peer review only - http://bmjopen.bmj.com/site/about/guidelines.xhtml

[9] School of Business, Warwick University, Warwick, United Kingdom

[10] Central Clinical School, Monash University, Melbourne, Victoria, Australia

[11] Women's and Babies Division, Women's and Children's Hospital, Adelaide, South Australia, Australia

[12] Division of Paediatrics, Women's and Children's Hospital, Adelaide, South Australia, Australia

[13] South Australian Health and Medical Research Institute, South Australia, Australia

[14] School of Public Health, The University of Adelaide, Adelaide, South Australia, Australia

[15] Discipline of Public Health, Flinders University, South Australia, Australia

[16] Discipline of Paediatrics, Flinders University, South Australia, Australia

[17] SA Health, South Australian Government, Australia

[18] Discipline of Paediatrics, Lyell McEwin Hospital, South Australia, Australia

[19] Discipline of Women's Health, Lyell McEwin Hospital, South Australia, Australia

[20] Discipline of Paediatrics, University of Sydney, Sydney, New South Wales, Australia

[21] Children's Hospital Westmead, Sydney, New South Wales, Australia

[22] Discipline of Public Health, Australian National University, Canberra,

Key words: COVID-19, Influenza, vaccine, medically at risk children

Correspondence: Prof. Helen Marshall, Vaccinology and Immunology Research Trials Unit, Women's and Children's Hospital, Adelaide, South Australia, Australia; phone +61 (08) 8313 4086, fax +61 (08) 8313 4086, email helen.marshall@adelaide.edu.au

For peer review only

For peer review only - http://bmjopen.bmj.com/site/about/guidelines.xhtml

ABSTRACT

Introduction: Children with chronic medical diseases are at an unacceptable risk of hospitalisation and death from influenza and coronavirus 2 (SARS-CoV-2) infections. Over the past two decades, behavioural scientists have learnt how to design non-coercive "nudge" interventions to encourage positive health behaviours. Our study aims to evaluate the impact of multi-component nudge interventions on the uptake of coronavirus disease (COVID-19) and influenza vaccines in medically at risk children.

Methods and analyses: Two separate randomized controlled trials (RCTs), each with 1038 children, will enrol a total of approximately 2076 children with chronic medical conditions who are attending tertiary hospitals in South Australia, Western Australia, and Victoria. Participants will be randomly assigned (1:1) to the standard care or intervention group. The nudge intervention in each RCT will consist of three text message reminders with four behavioural nudges including (i) social norm messages, (ii) different messengers through links to short educational videos from a paediatrician, medically at risk child and parent, and nurse, (iii) a pledge to have their child or themselves vaccinated and (iv) information salience through links to the current guidelines and vaccine safety information. The primary outcome is the proportion of medically at risk children who receive at least one dose of vaccine within 3 months of randomisation. Logistic regression analysis will be performed to determine the effect of the intervention on the probability of vaccination uptake.

Ethics and dissemination: The protocol and study documents have been reviewed and approved by the Women's and Children's Health Network Human Research Ethics Committee (HREC/22/WCHN/2022/00082). Results will be published via peer-reviewed journals and presented at scientific meetings and public forums.

ClinicalTrials.gov Identifier: NCT05613751

For peer review only - http://bmjopen.bmj.com/site/about/guidelines.xhtml

Article Summary

Strengths and limitations of this study

- The Nudgeathon involved collaboration with various stakeholders who co-designed the nudge interventions.
- Nudge interventions are currently undergoing evaluation in randomized controlled trials, with an additional assessment of cost-effectiveness.
- The waiver of consent in our study helps mitigate selection bias.
- The general public has experienced multiple COVID-19 vaccine campaigns, which may lead to fatigue with any further COVID-19 promotion strategies.

For peer review only

For peer review only - http://bmjopen.bmj.com/site/about/guidelines.xhtml

INTRODUCTION

Children with chronic medical conditions represent a clinically vulnerable group in Australia with around 15% of children having either a respiratory, cardiac or neurological condition that increases their risk of hospitalisation and death from respiratory infections.[1] A systematic review and meta-analysis conducted by our group in 2017 demonstrates that compared with healthy peers, medically at risk children with influenza are at higher risks for intensive care unit (ICU) admission, mechanical ventilation and death.[2] They are also more likely to develop bacterial pneumonia or experience prolonged hospital length of stay.[2] Australian data on children ≤ 16 years admitted with acute respiratory illnesses and tested for influenza at sentinel hospitals between 2010 and 2019 demonstrate that comorbidities are an independent predictor of severe outcomes. Specifically, the odds of ICU admission are higher in those with any comorbidity (adjusted odds ratio (aOR): 1.36, 95% Confidence Interval [95% CI]: 1.05-1.77) compared with cases without any comorbidity. Children with diabetes (aOR: 3.22, 95% CI: 1.25; 8.23), cardiac (aOR: 1.93, 95% CI: 1.23-3.03), respiratory (aOR: 1.54, 95% CI: 1.08-2.21), or neurological comorbidities (aOR: 1.57, 95% CI: 1.25-1.98) are at greatest odds of ICU admission. Respiratory, neurological, cardiac, genetic, and hepatic comorbidities and diabetes were shown to be associated with prolonged hospitalisation, whereas respiratory, renal, cardiac, and other comorbidities increased the length of ICU stay.[3] The cost per episode of influenza-associated hospitalisation was estimated to be AUD $19,704 (95% CI: 11,715-27,693) for children with chronic lung conditions compared to AUD $4,557 (95% CI: 4,129-4,984) for children without.[4] Influenza vaccines are provided free to this group (≥ 6 months).[5] Our research has previously shown that parents of children with chronic medical conditions are less likely to have their children vaccinated unless recommended by their specialist.[6] The uptake of influenza vaccine at least once in the last two years among medically at risk children is 50% with annual vaccination only 32.8%.[7]

For peer review only - http://bmjopen.bmj.com/site/about/guidelines.xhtml

The coronavirus 2 (SARS-CoV-2) infection causing Coronavirus disease (COVID-19) may overall be milder in children compared to adults but a small proportion develop severe disease and are at risk of a debilitating multisystem-inflammatory syndrome (MIS-C) and/or long COVID (long lasting complications of COVID-19).[8] Children with chronic diseases are also at a higher risk of COVID-19 complications including pneumonia and respiratory failure and long-term health consequences.[4, 9] Long COVID is a multi-system illness characterised by ongoing persistent symptoms that can last for weeks or months following SARS-CoV-2 infection.[10] Chronic fatigue, short term memory problems, loss of taste and smell and long school absences were reported in long COVID cases.[11] COVID-19 vaccines are provided free to medically at risk children (≥ 6 months).[5] Of concern, two-dose COVID-19 vaccine uptake among children aged 5-11 years is at present approximately 40% nationally.[12] At a time when Australia is still experiencing a large number of SARS-CoV-2 infections, with greater risks to the individual and the health system of co-circulation of influenza and SARS-CoV-2 infections and emergence of new viral variants, it is critical that all efforts are made to improve vaccine uptake among these high-risk groups. Equally important is the opportunity to continue to evaluate the safety of COVID-19 vaccines in these groups including children with chronic conditions that are often excluded from clinical trials.

Lack of access and practical barriers to vaccines remain the leading cause of low vaccine uptake, which is especially relevant to patients whose care is primarily in a hospital setting. For children with chronic conditions the hospital often takes on the role of primary as well as tertiary care due to parental anxiety, life pressures, timeliness and access issues. Parents have identified barriers such as concern and lack of confidence in family physicians providing a vaccine recommendation for medically at risk children and access to vaccines in hospital

For peer review only - http://bmjopen.bmj.com/site/about/guidelines.xhtml

settings being sub-optimal. Mobility, transport, language, financial barriers, and remoteness are also reported barriers for children with chronic and complex conditions in accessing services.[13] For these groups, vaccination in the hospital setting, when patients are already onsite and engaged with a healthcare setting, would improve immunisation opportunities and protection against the threat of both influenza and SARS-CoV-2 infection.

Improving uptake of recommended vaccines in medically at risk children primarily attending hospitals requires an innovative rather than a population approach. Over the past two decades, behavioural scientists have learnt how to design non-coercive "nudge" strategies to promote positive behaviours in a range of contexts. In psychology and neuroscience, a dual process theory describes brain functioning as two types of cognitive processes - 'System 1' processes described as automatic, uncontrolled, effortless, associative, fast, unconscious and affective, and 'System 2' processes described as reflective, controlled, effortful, rule-based, slow, conscious and rational.[14] Although this 'dual process' model is considered as a theoretical basis for nudge theory, the nudge approach suggests that automatic decisions can be systematically triggered to improve health outcomes.[15] In other words, nudges are simple cues in our environment that influence people to behave in a certain way to achieve better personal or social goals without making a conscious decision to do so.[16] Using behavioural economics (a discipline that studies how individuals make choice) the environment in which a choice is being made can actively be designed to encourage better health-related choices.[17] A previous study showed nudges and reminders resulted in a decrease in energy-dense nutrient-poor foods in men and sugar-sweetened beverages in women, together with a reduction in body weight.[18] A "nudge" intervention needs to be simple and low cost and if proven successful can be simply incorporated into standard health care. For example, redesigning cardiac rehabilitation referral decision pathway from opt-in to opt-out referral, which automatically

For peer review only - http://bmjopen.bmj.com/site/about/guidelines.xhtml

identified eligible patients from the electronic health record and notified staff on the wards by using secure text messaging, increased cardiac rehabilitation referral rates from 15% to 85%.[19] Nudge-based interventions to address vaccine hesitancy include using reminders and recall, changing the way information is framed and delivered to an intended audience, changing the messenger delivering information, invoking social norms and emotional affect (e.g., through storytelling, dramatic narratives and graphical presentations), and offering incentives or changing defaults. Nudge-based interventions show potential to increase vaccine confidence and uptake. Interestingly, strategies with educational approaches are less effective (or not effective at all) in improving vaccine uptake.[20] The most promising evidence exists for nudges that offer incentives to parents and healthcare workers, that make information more salient, that frame vaccination as the default, or that use trusted messengers to deliver information.[21, 22] Normalizing vaccination and peer influence can activate social tendencies to join others.[23] Several studies have explored the impact of different interventions on vaccination rates and intentions. A large study with 57,893 participants was conducted in a Northern California health system, and found that personal reminder messages increased booster vaccination rates.[24] Among 964,870 participants in 691,820 households in Denmark, two strategies increased influenza vaccination rates.[25, 26] In the US, a study found that short video messages addressing specific COVID-19 vaccine concerns increased vaccination intentions.[27] In a March 2021 study with 1,595 participants in Japan, different messages were tested to encourage COVID-19 vaccination. The 'influence-gain' message was effective for older adults. 'Comparison' and 'influence-loss' messages reinforced existing intentions among older adults.[23] In our previous study conducted at a tertiary paediatric hospital in Adelaide (n=600), a significantly greater proportion receiving the SMS intervention were vaccinated with 38.6% in the SMS intervention group compared with 26.2% in the control group.[28] A recent study was conducted in older adult populations (n=48,125) and found behavioural

For peer review only

For peer review only - http://bmjopen.bmj.com/site/about/guidelines.xhtml

nudges, electronically delivered letters or centralized written reminders, significantly increased influenza vaccination uptake in Finland.[29] A randomized controlled trial (RCT) of a nudge intervention that included text message reminders demonstrated that the first reminder (n=93,354) increased appointment and COVID-19 vaccination rates by 6.07% and 3.57% and that the second reminder (n=67,092) increased those by 1.65% and 1.06% respectively in the early stages of the COVID-19 vaccine rollout.[30] Another RCT was conducted a few months later in a younger population (mean age 39 years; n = 142,428) and no SMS message did substantially better or worse than the control whether vaccination rates were measured one week after the messages were sent or at the end of the study period.[31] The difference between two studies may suggest that nudges help early in vaccination campaigns, but the efficacy decays. However, nudging-based interventions have shown potential to increase vaccine confidence and uptake in many studies, but further evidence is needed for the development and evaluation of clear recommendations.[22]

Our team recently conducted an RCT from April 15 to September 30, 2021 and found a 47% relative increase in uptake of influenza vaccine in medically at risk children using a simple SMS nudge co-designed with paediatricians.[28] If our text based nudges with links to short educational videos prove to be successful in improving influenza and COVID-19 vaccine uptake in medically at risk children, they can easily be implemented as a promising, valuable, low-cost and long-term tool at a national level.

However, a challenge is how behavioural economic concepts and principles can be effectively applied by local community stakeholders to identify barriers and create an innovative intervention to complement the current immunisation programs. To bridge this gap, a Nudgeathon,[32] a crowdsourcing interaction in which decision groups draw on insights and

For peer review only - http://bmjopen.bmj.com/site/about/guidelines.xhtml

methods from nudge theory, augmented by design thinking and drama theory to devise implementable solutions to major behavioural policy problems, was launched.[32] These events are typically conducted over 1-2 days, bringing together a diverse range of stakeholders (many who are new to behavioural science) to generate new approaches to complex problems in a time-pressured setting. Three dozen Nudgeathons [32] around the world have been conducted to address multiple issues (e.g. handwashing among health providers, increasing regular HIV/sexually transmitted infection testing in men [17, 33]) but to our knowledge, there has not been a Nudgeathon for the immunisation programs. The MINDSPACE framework, which provides a list of behaviour change techniques including Messenger, Incentives, Norms, Defaults, Salience, Priming, Affect, Commitments, Ego, that target the automatic decision processes,[34] was used to identify and generate potential nudges at the Nudgeathon. Our goal through the Nudgeathons is to gain valuable "Customer Insight" within the Australian context, fostering a profound understanding of parents' experiences, beliefs, needs, and desires, while also identifying the practical and structural challenges they encounter. It's important to recognise that attempts to promote behaviour change without considering these contextual factors often result in frustration. Behaviour change initiatives can be contentious, involving intricate trade-offs and often addressing areas where government decisions are controversial such as COVID-19 vaccination policies. Consequently, innovative approaches like Nudgeathons may be essential to engage the public effectively in exploring acceptable courses of action.

To our knowledge, this will be the first study assessing the effectiveness and cost-effectiveness of a co-designed multi-component nudge intervention on improving influenza and COVID-19 vaccine uptake in medically at-risk children. Vaccine hesitancy and access barriers are a major barrier that reduces the progress of management of vaccine preventable diseases in children.

For peer review only - http://bmjopen.bmj.com/site/about/guidelines.xhtml

Identifying solutions to improving COVID-19 and influenza vaccine uptake in medically at risk children is critical, as these children are more vulnerable to severe COVID-19 and influenza than healthy children.

Therefore, this study aims to design nudge interventions and evaluate the impact of multi-component nudge interventions on the uptake of influenza and COVID-19 vaccines among medically at risk children.

METHODS AND ANALYSIS

Recommendations for Intervention Trials (SPIRIT) checklist was used when developing this protocol.[35] The first participant was randomised on 29 November 2022 and the study is expected to be complete by 31 December 2023. The master protocol was devised to evaluate the impact of multi-component nudge interventions on the uptake of COVID-19 and influenza vaccines in two distinct sub-populations: medically at-risk children and pregnant women, employing a unified and overarching design. The information pertaining to medically at-risk children is presented here. The protocol information regarding the sub-study involving pregnant women was published elsewhere.[36]

Nudge intervention

In August 2022, a Nudgeathon was held to design appropriate nudges for inclusion in the trial. Fourteen participants including medically at risk children, their parents, paediatricians, nurses, hospital administrative personnel, behavioural scientists, psychologists and graphic designers with diverse skills from South Australia (SA), Western Australia and Victoria participated in the Nudgeathon. The participants learnt about behavioural science and successful application of nudges in different disciplines. They were assigned into small groups ensuring that each

For peer review only - http://bmjopen.bmj.com/site/about/guidelines.xhtml

group comprised participants with different skills. The individual groups were requested to create a nudge for improving either COVID-19 or influenza vaccine uptake in medically at risk children. At the end of the Nudgeathon each group presented their nudges and one nudge for each condition was chosen by the team to develop the nudge interventions in the following randomised control trials. Some of the most robust effects that have been found to have strong impacts on behaviour[34] such as Messenger, Norms, Salience and Commitments were used to generate nudge interventions in our study. The carefully chosen nudge for both COVID-19 and influenza vaccines consists of three SMS text messages that are sent to parents of medically at risk children from the participating hospitals four weeks apart. The first text message is sent one week prior to a scheduled paediatric clinic appointment and provides a link to current national guidelines on COVID-19 or influenza vaccination in children with chronic illness and a link to a video of a paediatrician explaining the benefits of COVID-19 or influenza vaccination in medically at risk children. The second text message provides a link to a video of a child with chronic illness and their parent giving their opinion on the protection of medically as risk children against SARS-CoV-2 or influenza infections. The third text message provides a link to a video of a nurse discussing the potential adverse outcomes of not being vaccinated against SARS-CoV-2 or influenza infections. The first and second text messages also provide the option for a parent to agree to vaccinate their children or opt out from receiving further reminders.

The study is aligned with the MINDSPACE Framework, a behavioural change model developed by the UK government's Behavioural Insights Team in 2010. The decision to incorporate a blend of behavioural nudges in the intervention stems from the notion that a multifaceted approach can potentially yield a more substantial impact on behaviour. Combining various nudges is often considered a strategy to enhance the likelihood of success,

For peer review only - http://bmjopen.bmj.com/site/about/guidelines.xhtml

as different individuals may respond diversely to different nudge effects.[37, 38] This approach seeks to cast a broader net to appeal to a more extensive spectrum of people and behaviours, leveraging the effects encapsulated in the acronym MINDSPACE, which includes Messenger, Incentives, Norms, Defaults, Salience, Priming, Affect, Commitment, and Ego. Significantly, there is a considerable overlap among these effects, and the most effective interventions will invariably integrate various elements.

Primary objectives

- To determine the proportion of medically as risk children in the intervention versus standard care (non-intervention) group receiving at least one dose of the seasonal influenza vaccine within 3 months after randomisation, as assessed using the Australian Immunisation Register (AIR)
- To determine the proportion of medically as risk children in the intervention versus standard care (non-intervention) group receiving at least one dose of a COVID-19 vaccine within 3 months after randomisation, as assessed using the AIR.

The AIR is a national register that records vaccines given to all people in Australia including vaccines given under the National Immunisation Program, including school programs, and privately, such as for influenza. A recognised vaccination provider including doctors, such as a family physician or community health centre, are required to update the AIR once an influenza, COVID-19 or any vaccine listed in the National Immunisation Program Schedule is administered.

Secondary objectives

For peer review only - http://bmjopen.bmj.com/site/about/guidelines.xhtml

- To identify socio-demographic characteristics and medical risk factors associated with COVID-19 or influenza vaccination in medically at risk children
- To assess timeliness of influenza and COVID-19 vaccine uptake among medically at risk children during the study period by determining the proportion of medically at risk children who receive the COVID-19 or influenza vaccine by month throughout the study period.
- To estimate the cost-effectiveness of proven interventions compared to standard care in hospital settings

Study design and study population

These are parallel-group randomised controlled trials (RCTs, ClinicalTrials.gov Identifier: NCT05613751) designed to measure the impact of a nudge intervention on receipt of one dose of the COVID-19 or influenza vaccine in medically at risk children who attend a clinic at tertiary hospitals in South Australia, Western Australia and Victoria. Medically at risk children will be enrolled at seven hospitals across South Australia (Women's and Children's Hospital, Flinders Medical Centre and the Lyell McEwin Hospital), Victoria (The Royal Children's Hospital) and Western Australia (Perth Children's Hospital). Each of these hospitals covers different socio-demographic areas and patient sources ensuring our research is generalisable. COVID-19 or influenza vaccination status of medically at risk children attending the participating hospitals will be determined by checking the AIR. The inclusion and exclusion criteria are listed in Table 1.

For peer review only - http://bmjopen.bmj.com/site/about/guidelines.xhtml

Table 1 Inclusion and exclusion criteria

	Exclusion criteria	Inclusion criteria
COVID vaccine [39]	• Known contraindications to COVID-19 vaccine<br>• Up to date for COVID-19 vaccine (≥ two doses) at the time of enrolment,<br>• Sibling of a child already enrolled in the trial (only the sibling who is eligible and scheduled to attend a paediatric clinic first will be eligible)<br>• Previous participation in the influenza nudge RCT	Medically at risk children aged 5 years to 18 years with a cardiac, endocrine, respiratory, gastrointestinal, haematological, musculoskeletal, neurological condition
Influenza vaccine [5]	• Known contraindications to influenza vaccine<br>• Already received an influenza vaccine during the flu season in 2023<br>• Sibling of a child already participating in the trial (the sibling who is eligible and scheduled to attend a paediatric clinic first will be eligible)	Children aged ≥6 months and < 18 years with medical conditions specified in this list: immunocompromising conditions including malignancy, chronic steroid use, haematopoietic stem cell transplant; functional or anatomical asplenia including sickle cell disease or other haemoglobinopathies, congenital or acquired asplenia (for example, splenectomy) or hyposplenia; cardiac disease including cyanotic congenital heart disease, congestive heart failure, coronary

For peer review only

For peer review only - http://bmjopen.bmj.com/site/about/guidelines.xhtml

	Exclusion criteria	Inclusion criteria
	• Previous participation in the COVID-19 nudge RCT	artery disease; chronic respiratory conditions including suppurative lung disease, bronchiectasis, cystic fibrosis, chronic obstructive pulmonary disease, severe asthma (requiring frequent medical consultations or the use of multiple medicines); chronic neurological conditions including hereditary and degenerative CNS diseases, seizure disorders, spinal cord injuries, neuromuscular disorders; chronic metabolic disorders including Type 1 or 2 diabetes, amino acid disorders, carbohydrate disorders, cholesterol biosynthesis disorders, fatty acid oxidation defects, lactic acidosis, mitochondrial disorders, organic acid disorders, urea cycle disorders, vitamin/cofactor disorders, porphyria; chronic renal failure; children aged 5 to 10 years receiving long term aspiring therapy; Down syndrome; obesity (body mass index ≥30 kg/m2); children born less than 37 weeks gestation

331

For peer review only - http://bmjopen.bmj.com/site/about/guidelines.xhtml

Randomisation

Medically at risk children will be randomised to the intervention and standard care groups in a 1:1 ratio. The randomisation list will be generated using R version 4.02 by an independent statistician who will not be involved with the conduct or analysis of the RCT. Allocations will be performed using randomly permuted blocks, stratified by hospital (i.e., separate randomisation sequence used in each hospital). Randomisation will be done on a password protected REDCap database securely held on the University of Adelaide server.

Endpoint measurements will involve low level contact of study staff, however when contact is required (such as AIR/ confirmation of influenza/COVID-19 vaccine receipt etc), this will be carried out by trial staff shielded from information that might reveal trial group assignment. The study statistician undertaking the analysis and study investigators will remain blinded to trial intervention assignment.

Study processes

Medically at risk children attending clinics at the hospitals will be identified from Outpatients' Department's appointment lists. Research nurses will assess COVID-19 or influenza vaccination status of children on the AIR to screen for eligibility. Medically at risk children will be randomised to the intervention arm or the standard care arm on REDCap. Demographic data will be obtained from hospital medical records. Using the Norms, Messenger, Salience and Commitment nudge strategies, parents of medically at risk children randomised to the intervention arm will receive a maximum of three SMS reminders from the hospital using 'Message Media' software from the hospitals. The SMS messages will comprise a brief message that many children with chronic illness obtain the COVID-19 or influenza vaccine (Norms) and a reminder for the child to obtain the vaccine. Parents of medically at risk children

For peer review only - http://bmjopen.bmj.com/site/about/guidelines.xhtml

have the option of responding to the message by 1) agreeing to obtain the vaccine (Commitment), 2) stating that the participant has already received the vaccine or 3) requesting to opt out from receiving further reminders (first and second SMS messages). Each SMS message also provides a link to a video of (Messager): 1) a paediatrician discussing the potential serious health consequences of SARS-CoV-2 or influenza infections (first SMS), 2) a medically at risk child and their parent stating that the child received the vaccine and that the child is protected from serious adverse effects of SARS-CoV-2 or influenza infections (second SMS), 3) a nurse stating the benefits of COVID-19 or influenza vaccination for medically at risk children (third SMS). In addition, the first SMS also states children with special medical conditions are at increased risk of severe COVID-19 or Influenza disease and provides a link to the current guidelines and safety information about COVID-19 or influenza vaccination (Salience). The paediatrician and nurse assumed the roles of healthcare experts, while the child with medical risks and their parent acted as relatable peers with a similar background. They served as influential messengers in persuading parents to vaccinate their children. The message highlighting the fact that many children with chronic illnesses receive the COVID-19 or influenza vaccine utilised a social norms approach, conveying what other parents in similar situations typically do. Meanwhile, the message emphasising the increased risk of severe COVID-19 or influenza for children with special medical conditions was designed to capture parents' attention, as it is information more likely to be comprehensible and directly relevant to their own children. The response options were strategically crafted to foster reciprocity and serve as commitments. The first SMS is sent approximately one week prior to a scheduled clinic visit. The second SMS message is sent four weeks after the first message to those who have not received a dose of COVID-19 or influenza vaccine after the first SMS (confirmed on AIR) and not requested to opt out. The third SMS message is sent four weeks after the second message to those who have not received a dose of COVID-19 or influenza vaccine after the

For peer review only - http://bmjopen.bmj.com/site/about/guidelines.xhtml

second SMS (confirmed on AIR) and not requested to opt out. The AIR will be checked at 3 months after randomisation to assess whether medically at risk children have received a dose of COVID-19 or influenza vaccine. All data will be stored securely on a password protected REDCap database held by the University of Adelaide. All data will be de-identified prior to presentation and publication.

Study monitoring and surveillance

The nudge is a behavioural change intervention and hence there are no risks related to invasive procedures or investigational medications. Any risks of mental distress associated with receiving the text message is expected to be very low and the parents have the option of opting out from receiving the further text messages after the first one. However, a risk assessment and management plan has been developed for this study. Moreover, the study management committee comprising the chief investigator, site investigators, study coordinators and statistician will carefully monitor the progress of the study.

This trial has certain limitations. The nudge interventions incorporated a blend of various nudge techniques, and we did not assess the individual effects of these techniques in our study. We included links within the text messages, and it was the parents' responsibility to click on these links. If the links were not clicked, the nudge interventions may not have been as effectively implemented as originally intended.

Sample size and statistical analysis plan

To detect an increase in the vaccination rate from ~50% in the control group to 60% in the intervention group, which is considered a clinically relevant impact, with 90% power (two-sided test with alpha = 0.05), a sample size of n = 519 per group (1038 total) is required. Should

For peer review only - http://bmjopen.bmj.com/site/about/guidelines.xhtml

407 the vaccination rate in the control arm be higher than 50% or lower than 40%, this sample size will still provide at least 90% power to detect a 10% absolute increase in vaccination with the nudge intervention. We aim to enrol at least 1038 medically at risk children to each of the two RCTs across the participating hospitals.

412 Statistical analyses will be conducted on an Intention To Treat basis according to a pre-specified statistical analysis plan. Baseline characteristics including age, socio-economic status based on postcode, and ethnicity will be reported for each group using percentages, means with standard deviations or medians with ranges as appropriate. The vaccination rate will be compared between intervention and control groups using logistic regression, with adjustment only made for participating hospitals (treated as a categorical fixed effect). Intervention effects will be reported as odds ratios with 95% confidence intervals. Patient characteristics associated with vaccination will be investigated using multivariable logistic regression models. Cost-effectiveness analysis of the nudge intervention will be compared to standard care from the healthcare payer perspective. The primary cost-effectiveness analysis will estimate the incremental cost per additional person vaccinated, in each of the two RCTs. Secondary analyses for the trial focused on influenza vaccination will estimate the cost per quality-adjusted life year (QALY) gained. The study results will be used to estimate the impact of the nudge intervention on health-related quality of life by calculating the QALYs gained as a result of the increased vaccine uptake. Implementation costs will be obtained from the study budget and costs related to research activities will be excluded. Estimated cost offsets to the health system associated with influenza related disease (e.g. hospitalisations and emergency visits) will be derived from the literature and calculated using cost weights for Australian Refined Diagnosis Related Groups (AR-DRGs). The cost-effectiveness evaluation will follow standard reporting guidelines in the CHEERS statement.[40]

For peer review only - http://bmjopen.bmj.com/site/about/guidelines.xhtml

Patient and Public Involvement

In our previous survey [6], 539 parents were interviewed, revealing that parents of children with chronic medical conditions were less likely to vaccinate their children unless recommended by a specialist. Interventions based on nudging in our previous study [28] showed potential in increasing vaccine uptake. Consequently, the study aims, design, and outcome measures have been informed by the priorities, experiences, and preferences of parents. Medically at risk children and their parents actively contributed to the study's design during the Nudgeathon. Their insights played a crucial role in designing the nudge interventions, making it more relevant and patient-centred. The study results will be disseminated to the trial participants, which includes medically at risk children and their parents, through various accessible means, such as academic publication and lay summaries on our research unit's webpage, to ensure that the findings are easily understood and meaningful to this group.

ETHICS AND DISSEMINATION

The current protocol version 2.0 (05-01-2023) and all study material have been reviewed and approved by the Women's and Children's Health Network Human Research Ethics Committee (HREC/2022/00082) and research governance approval has been obtained from the Women's and Children's Hospital, Flinders Medical Centre, and Lyell McEwin Hospital in SA, The Royal Children's Hospital in VIC, and Perth Children's Hospital in Western Australia. The written consent was obtained from all stakeholders including health service leaders and clinicians and health consumers to participate in the Nudgeathons. A waiver of consent was approved for medically at risk children to participate in this trial and receive SMS messages in accordance with the National Statement on Ethical Conduct in Human Research. Three criteria

For peer review only - http://bmjopen.bmj.com/site/about/guidelines.xhtml

guide this. Firstly, there are no estimated risks or harms associated with study procedures. Any risk of psychosocial distress associated with receiving the nudge interventions is very unlikely. Secondly, it would not be possible to obtain informed consent without threatening the validity of the trials. It would be impossible to assess the effectiveness of the nudge by recruiting parents of medically at risk children and informing them about the study and then randomising them not to receive the nudge. Thirdly, as influenza and COVID-19 vaccines are recommended for all medically at risk children aged ≥ 6 months, all data for the influenza and COVID-19 RCTs will be collected as part of routine care, including patient demographics and specialties seen. In addition this methodology was approved and successfully applied in the Flutext-4U study.[41] We identified a significant difference in uptake of influenza vaccine in medically at risk children in the Flutext-4U study, likely due to this being a population inclusive study unaffected by healthy selection bias.[28] The study is being conducted in accordance with the Declaration of Helsinki and the International Conference on Harmonization Guidelines on Good Clinical Practice. The trial is being conducted in compliance with the current version of the protocol. Any change to the protocol that affects scientific content, study design, and participating will be considered an amendment and will be submitted to HREC for review and approval prior to implementation.

Participant confidentiality is strictly held in trust by the investigators. Therefore, study documentation, data, and all other information generated will be held in strict confidence. No information concerning the study, or the data will be released to any unauthorised third party. Study participant research data, which is for purposes of statistical analysis and scientific reporting, will be transmitted to and stored at the Women's and Children's Hospital. At the end of the study, all study databases will be de-identified and archived at the University of Adelaide. After the completion of the study, the de-identified and aggregated results will be

For peer review only - http://bmjopen.bmj.com/site/about/guidelines.xhtml

presented at scientific forums and submitted for publication in peer-reviewed journals. The results will be disseminated regardless of the direction of effect. The findings of the study will be communicated to key stakeholders.

The burden of the intervention is planned to be assessed with input from medically at risk children’s parents using a short evaluation questionnaire. Special recognition and appreciation were extended to the medical at risk children and their parents who participated in the Nudgeathon to highlight their valuable contributions to the study's design and success.

For peer review only

For peer review only - http://bmjopen.bmj.com/site/about/guidelines.xhtml

AUTHORS CONTRIBUTIONS

HM, IV, JO, MD, CB, BW, PA, JD, JC, TS, JK, NS, MC, DM, DS, GD, SC, JT, NW and LW conceptualised the interventions, contributed to the design of the study protocol, and acquired funding. TS provided the statistical consult. BW drafted the manuscript. MD, CB, JD, JC, JK, NS, MC, DM, DS, GD, SC, NW and LW critically reviewed. HM, BW, IV, JO, TS, JT and PA critically reviewed and edited the manuscript. HM, BW, IV, JO, MD, CB, PA, JD, JC, TS, JK, NS, MC, DM, DS, GD, SC, JT, NW and LW approved its final version.

ACKNOWLEDGEMENTS

We acknowledge the following study coordinators for their contribution to study set up and recruitment: Kirsty Herewane (Women's and Children's Hospital), Joanne Koch (Lyell McEwin Hospital), Lauren Thompson (Flinders Medical Centre), Ashleigh Rak (Royal Children's Hospital), Deborah Pidd (Mercy Hospital for Women), Louisa Paparo, Rebecca Pavlos and Erin Van Der Helder (Perth Children's Hospital). We would also like to express our gratitude to all participants who were involved in this study.

FUNDING STATEMENT

This work is supported by the National Health and medical Research Council of Australia [NHMRC Partnership grant APP2014684]. Additional in-kind funding is provided by funding partners including the Department of Health, South Australia Government; Department of Health, Victorian Government; Department of Health, Western Australian Government; Women's and Children's Health Network; Southern Adelaide Local Health Network; Northern Adelaide Local Health Network; Women's and Children's Hospital Foundation; Department of Trade and Investment, South Australian Government, in support of the NHMRC Partnership grant.

For peer review only

For peer review only - http://bmjopen.bmj.com/site/about/guidelines.xhtml

COMPETING INTERESTS STATEMENT

HM acknowledges support from the National Health and Medical Research Council of Australia: Practitioner Fellowship (APP1155066). CCB acknowledges support from the National Health and Medical Research Council of Australia: Investigator Grant (APP1173163). HM is an independent investigator on clinical trials of investigational vaccines manufactured by pharmaceutical companies including Pfizer, ILiAD Biotechnologies, and Merck. The institution at which HM, BW and PA are employed has received funding for investigator-led research from GlaxoSmithKline, Sanofi-Pasteur, and Pfizer Vaccines. All authors receive no personal payments from industry. There are no other conflicts of interest to declare.

For peer review only

For peer review only - http://bmjopen.bmj.com/site/about/guidelines.xhtml

REFERENCES

[1] Australian Institute of Health and Welfare 2020. Australia's children. Cat. no. CWS 69. Canberra: AIHW. https://www.aihw.gov.au/reports/children-youth/australias-children/contents/executive-summary. Australia's Children. DOI: 10.25816/5ebca4d0fa7dd

[2] Tuckerman J, Misan S, Crawford NW, Marshall HS. Influenza in Children With Special Risk Medical Conditions: A Systematic Review and Meta-analysis. The Pediatric infectious disease journal. 2019;38:912-9.

[3] Norman DA, Cheng AC, Macartney KK, Moore HC, Danchin M, Seale H, et al. Influenza hospitalizations in Australian children 2010-2019: The impact of medical comorbidities on outcomes, vaccine coverage, and effectiveness. Influenza and other respiratory viruses. 2022;16:316-27.

[4] Homaira N, Briggs N, Oei JL, Hilder L, Bajuk B, Snelling T, et al. Impact of influenza on hospitalization rates in children with a range of chronic lung diseases. Influenza and other respiratory viruses. 2019;13:233-9.

[5] Australian Technical Advisory Group on Immunisation (ATAGI). Australian Immunisation Handbook, Australian Government Department of Health and Aged Care, Canberra, 2022, immunisationhandbook.health.gov.au

[6] Tuckerman J, Crawford NW, Marshall HS. Disparities in parental awareness of children's seasonal influenza vaccination recommendations and influencers of vaccination. PloS one. 2020;15:e0230425.

[7] Tuckerman J, Misan S, Salih S, Joseph Xavier B, Crawford NW, Lynch J, et al. Influenza vaccination: Uptake and associations in a cross-sectional study of children with special risk medical conditions. Vaccine. 2018;36:8138-47.

[8] Sudre CH, Murray B, Varsavsky T, Graham MS, Penfold RS, Bowyer RC, et al. Attributes and predictors of long COVID. Nature medicine. 2021;27:626-31.

For peer review only

For peer review only - http://bmjopen.bmj.com/site/about/guidelines.xhtml

551 [9] Moeller A, Thanikkel L, Duijts L, Gaillard EA, Garcia-Marcos L, Kantar A, et al. COVID-
552 19 in children with underlying chronic respiratory diseases: survey results from 174 centres.
553 ERJ open research. 2020;6.
554 [10] Australian Institute of Health and Welfare (2022) Long COVID in Australia – a review of
555 the literature, catalogue number PHE 318, AIHW, Australian Government.
556 [11] Ludvigsson JF. Case report and systematic review suggest that children may experience
557 similar long-term effects to adults after clinical COVID-19. Acta paediatrica (Oslo, Norway :
558 1992). 2021;110:914-21.
559 [12] https://www.health.gov.au/sites/default/files/2023-03/covid-19-vaccine-rollout-update-3-
560 march-2023.pdf. COVID-19 Vaccine Roll-out (02 March 2023).
561 [13] Pearce A, Marshall H, Bedford H, Lynch J. Barriers to childhood immunisation: Findings
562 from the Longitudinal Study of Australian Children. Vaccine. 2015;33:3377-83.
563 [14] Vlaev I, King D, Dolan P, Darzi A. The Theory and Practice of "Nudging": Changing
564 Health Behaviors. Public Administration Review. 2016;76:550-61.
565 [15] Marteau TM, Ogilvie D, Roland M, Suhrcke M, Kelly MP. Judging nudging: can nudging
566 improve population health? BMJ (Clinical research ed). 2011;342:d228.
567 [16] Simon C, Tagliabue M. Feeding the behavioral revolution: Contributions of behavior
568 analysis to nudging and vice versa. Journal of Behavioral Economics for Policy. 2018;2:91-7.
569 [17] Ong JJ, Chow EPF, Read D, Taj U, Lee D, Vlaev I. Nudgeathons to control HIV: designing
570 strategies using behavioural economics. AIDS. 2020;34:2337-40.
571 [18] Kerr DA, Harray AJ, Pollard CM, Dhaliwal SS, Delp EJ, Howat PA, et al. The connecting
572 health and technology study: a 6-month randomized controlled trial to improve nutrition
573 behaviours using a mobile food record and text messaging support in young adults. Int J Behav
574 Nutr Phys Act. 2016;13:52.

For peer review only

For peer review only - http://bmjopen.bmj.com/site/about/guidelines.xhtml

[19] Adusumalli S, Jolly E, Chokshi NP, Gitelman Y, Rareshide CAL, Kolansky DM, et al. Referral Rates for Cardiac Rehabilitation Among Eligible Inpatients After Implementation of a Default Opt-Out Decision Pathway in the Electronic Medical Record. JAMA network open. 2021;4:e2033472.

[20] Dubé E, Gagnon D, MacDonald NE. Strategies intended to address vaccine hesitancy: Review of published reviews. Vaccine. 2015;33:4191-203.

[21] Tentori K, Pighin S, Giovanazzi G, Grignolio A, Timberlake B, Ferro A. Nudging COVID-19 Vaccine Uptake by Changing the Default: A Randomized Controlled Trial. Med Decis Making. 2022;42:837-41.

[22] Renosa MDC, Landicho J, Wachinger J, Dalglish SL, Barnighausen K, Barnighausen T, et al. Nudging toward vaccination: a systematic review. BMJ Glob Health. 2021;6.

[23] Sasaki S, Saito T, Ohtake F. Nudges for COVID-19 voluntary vaccination: How to explain peer information? Soc Sci Med. 2022;292:114561.

[24] Chang TY, Jacobson M, Shah M, Kopetsky M, Pramanik R, Shah SB. Reminders, but not monetary incentives, increase COVID-19 booster uptake. Proc Natl Acad Sci U S A. 2023;120:e2302725120.

[25] Johansen ND, Vaduganathan M, Bhatt AS, Lee SG, Modin D, Claggett BL, et al. Electronic nudges to increase influenza vaccination uptake in Denmark: a nationwide, pragmatic, registry-based, randomised implementation trial. Lancet. 2023;401:1103-14.

[26] Johansen ND, Vaduganathan M, Bhatt AS, Lee SG, Modin D, Claggett BL, et al. Nationwide Utilization of Danish Government Electronic Letter System for Increasing InFLUenza Vaccine Uptake (NUDGE-FLU): Study Protocol for a Nationwide Randomized Implementation Trial. Am Heart J. 2023.

[27] Jensen UT, Ayers S, Koskan AM. Video-based messages to reduce COVID-19 vaccine hesitancy and nudge vaccination intentions. PloS one. 2022;17:e0265736.

For peer review only

For peer review only - http://bmjopen.bmj.com/site/about/guidelines.xhtml

[28] Tuckerman J, Harper K, Sullivan TR, Cuthbert AR, Fereday J, Couper J, et al. Short Message Service Reminder Nudge for Parents and Influenza Vaccination Uptake in Children and Adolescents With Special Risk Medical Conditions: The Flutext-4U Randomized Clinical Trial. JAMA pediatrics. 2023.

[29] Saaksvuori L, Betsch C, Nohynek H, Salo H, Sivela J, Bohm R. Information nudges for influenza vaccination: Evidence from a large-scale cluster-randomized controlled trial in Finland. PLoS Med. 2022;19:e1003919.

[30] Dai H, Saccardo S, Han MA, Roh L, Raja N, Vangala S, et al. Behavioural nudges increase COVID-19 vaccinations. Nature. 2021;597:404-9.

[31] Rabb N, Swindal M, Glick D, Bowers J, Tomasulo A, Oyelami Z, et al. Evidence from a statewide vaccination RCT shows the limits of nudges. Nature. 2022;604:E1-E7.

[32] Nudgeathon. [cited 27 March 2023]. Available from: https://www.nudgeathon.co.uk..

[33] Tran J, Vlaev I, Read D, Schmidtke KA, Chow EP, Lee D, et al. A qualitative evaluation of a Nudgeathon event for the co-design of sexual health campaign images targeting overseas-born men who have sex with men. Sexual Health. 2023.

[34] Dolan P, Hallsworth M, Halpern D, King D, Vlaev I. MINDSPACE: influencing behaviour for public policy. 2010.

[35] Chan AW, Tetzlaff JM, Gotzsche PC, Altman DG, Mann H, Berlin JA, et al. SPIRIT 2013 explanation and elaboration: guidance for protocols of clinical trials. BMJ (Clinical research ed). 2013;346:e7586.

[36] Andraweera PH, Wang B, Danchin M, Blyth C, Vlaev I, Ong J, et al. Randomised controlled trials of behavioural nudges delivered through text messages to increase influenza and COVID-19 vaccines among pregnant women (the EPIC study): study protocol. Trials. 2023;24:454.

For peer review only - http://bmjopen.bmj.com/site/about/guidelines.xhtml

[37] Howley P, Ocean N. Can nudging only get you so far? Testing for nudge combination effects. European Review of Agricultural Economics. 2022;49:1086-112.

[38] Cosic A, Cosic H, Ille S. Can nudges affect students' green behaviour? A field experiment. Journal of Behavioral Economics for Policy. 2018;2:107-11.

[39] Australian Technical Advisory Group on Immunisation (ATAGI). Australian Immunisation Handbook, Australian Government Department of Health and Aged Care, Canberra, 2022, https://www.health.gov.au/initiatives-and-programs/covid-19-vaccines/advice-for-providers/clinical-guidance/clinical-features.

[40] Husereau D, Drummond M, Augustovski F, de Bekker-Grob E, Briggs AH, Carswell C, et al. Consolidated Health Economic Evaluation Reporting Standards 2022 (CHEERS 2022) Statement: Updated Reporting Guidance for Health Economic Evaluations. Value Health. 2022;25:3-9.

[41] Tuckerman J, Harper K, Sullivan TR, Fereday J, Couper J, Smith N, et al. Using provider-parent strategies to improve influenza vaccination in children and adolescents with special risk medical conditions: a randomised controlled trial protocol. BMJ open. 2022;12:e053838.

For peer review only - http://bmjopen.bmj.com/site/about/guidelines.xhtml

STANDARD PROTOCOL ITEMS: RECOMMENDATIONS FOR INTERVENTIONAL TRIALS

SPIRIT 2013 Checklist: Recommended items to address in a clinical trial protocol and related documents*

Section/item	Item No	Description	Page and Line Number	Reason if not applicable
**Administrative information**				
Title	1	Descriptive title identifying the study design, population, interventions, and, if applicable, trial acronym	P1, L3	
Trial registration	2a	Trial identifier and registry name. If not yet registered, name of intended registry	P3, L73	
	2b	All items from the World Health Organization Trial Registration Data Set	NA	Registered at clinicaltrials.gov.
Protocol version	3	Date and version identifier	P21, L435	
Funding	4	Sources and types of financial, material, and other support	P23, L497-505	
Roles and responsibilities	5a	Names, affiliations, and roles of protocol contributors	P23, L482-488	
	5b	Name and contact information for the trial sponsor	NA	Investigator led study
	5c	Role of study sponsor and funders, if any, in study design; collection, management, analysis, and interpretation of data; writing of the report; and the decision to submit the report for publication, including whether they will have ultimate authority over any of these activities	NA	Investigator led study
	5d	Composition, roles, and responsibilities of the coordinating centre, steering committee, endpoint adjudication committee, data management team, and other individuals or groups overseeing the trial, if applicable (see Item 21a for data monitoring committee)	P19, L388-395	
**Introduction**				

For peer review only - http://bmjopen.bmj.com/site/about/guidelines.xhtml

Background and rationale	6a	Description of research question and justification for undertaking the trial, including summary of relevant studies (published and unpublished) examining benefits and harms for each intervention	P5-11, L85-241	
	6b	Explanation for choice of comparators	P7-8, L172-182	
Objectives	7	Specific objectives or hypotheses	P10-11, L241-264	
Trial design	8	Description of trial design including type of trial (eg, parallel group, crossover, factorial, single group), allocation ratio, and framework (eg, superiority, equivalence, noninferiority, exploratory)	P13-14, L293-329	
**Methods: Participants, interventions, and outcomes**				
Study setting	9	Description of study settings (eg, community clinic, academic hospital) and list of countries where data will be collected. Reference to where list of study sites can be obtained	P14, L319-329	
Eligibility criteria	10	Inclusion and exclusion criteria for participants. If applicable, eligibility criteria for study centres and individuals who will perform the interventions (eg, surgeons, psychotherapists)	P15-16, Table 1	
Interventions	11a	Interventions for each group with sufficient detail to allow replication, including how and when they will be administered	P17-18, L347-386	
	11b	Criteria for discontinuing or modifying allocated interventions for a given trial participant (eg, drug dose change in response to harms, participant request, or improving/worsening disease)	P19, L389-401	
	11c	Strategies to improve adherence to intervention protocols, and any procedures for monitoring adherence (eg, drug tablet return, laboratory tests)	P19, L389-401	
	11d	Relevant concomitant care and interventions that are permitted or prohibited during the trial	NA	Normal care as the intervention is a text message

For peer review only - http://bmjopen.bmj.com/site/about/guidelines.xhtml

Outcomes	12	Primary, secondary, and other outcomes, including the specific measurement variable (eg, systolic blood pressure), analysis metric (eg, change from baseline, final value, time to event), method of aggregation (eg, median, proportion), and time point for each outcome. Explanation of the clinical relevance of chosen efficacy and harm outcomes is strongly recommended	P20, L412-431
Participant timeline	13	Time schedule of enrolment, interventions (including any run-ins and washouts), assessments, and visits for participants. A schematic diagram is highly recommended (see Figure)	P11 L245-246; L254-258
Sample size	14	Estimated number of participants needed to achieve study objectives and how it was determined, including clinical and statistical assumptions supporting any sample size calculations	P19-22, L404-410
Recruitment	15	Strategies for achieving adequate participant enrolment to reach target sample size	P21-22, L442-460

Methods: Assignment of interventions (for controlled trials)

Allocation:

Sequence generation	16a	Method of generating the allocation sequence (eg, computer-generated random numbers), and list of any factors for stratification. To reduce predictability of a random sequence, details of any planned restriction (eg, blocking) should be provided in a separate document that is unavailable to those who enrol participants or assign interventions	P17, L333-338
Allocation concealment mechanism	16b	Mechanism of implementing the allocation sequence (eg, central telephone; sequentially numbered, opaque, sealed envelopes), describing any steps to conceal the sequence until interventions are assigned	P17, L333-338
Implementation	16c	Who will generate the allocation sequence, who will enrol participants, and who will assign participants to interventions	P17, L333-338

For peer review only - http://bmjopen.bmj.com/site/about/guidelines.xhtml

Blinding (masking)	17a	Who will be blinded after assignment to interventions (eg, trial participants, care providers, outcome assessors, data analysts), and how	P17, L333-338
	17b	If blinded, circumstances under which unblinding is permissible, and procedure for revealing a participant's allocated intervention during the trial	P17, L333-338
**Methods: Data collection, management, and analysis**			
Data collection methods	18a	Plans for assessment and collection of outcome, baseline, and other trial data, including any related processes to promote data quality (eg, duplicate measurements, training of assessors) and a description of study instruments (eg, questionnaires, laboratory tests) along with their reliability and validity, if known. Reference to where data collection forms can be found, if not in the protocol	P17, L347-351<br>P19, L382-386
	18b	Plans to promote participant retention and complete follow-up, including list of any outcome data to be collected for participants who discontinue or deviate from intervention protocols	P21-22, L442-460
Data management	19	Plans for data entry, coding, security, and storage, including any related processes to promote data quality (eg, double data entry; range checks for data values). Reference to where details of data management procedures can be found, if not in the protocol	P19, L382-386<br>P22, L462-471
Statistical methods	20a	Statistical methods for analysing primary and secondary outcomes. Reference to where other details of the statistical analysis plan can be found, if not in the protocol	P20, L412-431
	20b	Methods for any additional analyses (eg, subgroup and adjusted analyses)	P20, L416-417
	20c	Definition of analysis population relating to protocol non-adherence (eg, as randomised analysis), and any statistical methods to handle missing data (eg, multiple imputation)	P20, L412
**Methods: Monitoring**			

For peer review only - http://bmjopen.bmj.com/site/about/guidelines.xhtml

Data monitoring	21a	Composition of data monitoring committee (DMC); summary of its role and reporting structure; statement of whether it is independent from the sponsor and competing interests; and reference to where further details about its charter can be found, if not in the protocol. Alternatively, an explanation of why a DMC is not needed	P19, 393-395
	21b	Description of any interim analyses and stopping guidelines, including who will have access to these interim results and make the final decision to terminate the trial	NA
Harms	22	Plans for collecting, assessing, reporting, and managing solicited and spontaneously reported adverse events and other unintended effects of trial interventions or trial conduct	P19, L390-393
Auditing	23	Frequency and procedures for auditing trial conduct, if any, and whether the process will be independent from investigators and the sponsor	P19, 393-395
**Ethics and dissemination**			
Research ethics approval	24	Plans for seeking research ethics committee/institutional review board (REC/IRB) approval	P21, L435-440
Protocol amendments	25	Plans for communicating important protocol modifications (eg, changes to eligibility criteria, outcomes, analyses) to relevant parties (eg, investigators, REC/IRBs, trial participants, trial registries, journals, regulators)	P22, L458-460
Consent or assent	26a	Who will obtain informed consent or assent from potential trial participants or authorised surrogates, and how (see Item 32)	P21, L440-455
	26b	Additional consent provisions for collection and use of participant data and biological specimens in ancillary studies, if applicable	NA

view only

For peer review only - http://bmjopen.bmj.com/site/about/guidelines.xhtml

Confidentiality	27	How personal information about potential and enrolled participants will be collected, shared, and maintained in order to protect confidentiality before, during, and after the trial	P22, L462-471	
Declaration of interests	28	Financial and other competing interests for principal investigators for the overall trial and each study site	P25, L509-517	
Access to data	29	Statement of who will have access to the final trial dataset, and disclosure of contractual agreements that limit such access for investigators	P22, L462-471	
Ancillary and post-trial care	30	Provisions, if any, for ancillary and post-trial care, and for compensation to those who suffer harm from trial participation	NA	
Dissemination policy	31a	Plans for investigators and sponsor to communicate trial results to participants, healthcare professionals, the public, and other relevant groups (eg, via publication, reporting in results databases, or other data sharing arrangements), including any publication restrictions	P22-23, 473-482	
	31b	Authorship eligibility guidelines and any intended use of professional writers	P24, L484-489	
	31c	Plans, if any, for granting public access to the full protocol, participant-level dataset, and statistical code	P22, L467-471	
**Appendices**				
Informed consent materials	32	Model consent form and other related documentation given to participants and authorised surrogates	NA	A consent waiver was approved by ethics committee
Biological specimens	33	Plans for collection, laboratory evaluation, and storage of biological specimens for genetic or molecular analysis in the current trial and for future use in ancillary studies, if applicable	NA	No biological samples are collected.

*It is strongly recommended that this checklist be read in conjunction with the SPIRIT 2013 Explanation & Elaboration for important clarification on the items. Amendments to the protocol should be tracked and dated. The SPIRIT checklist is copyrighted by the SPIRIT Group under the Creative Commons "Attribution-NonCommercial-NoDerivs 3.0 Unported" license.

For peer review only - http://bmjopen.bmj.com/site/about/guidelines.xhtml

BMJ Open

Nudging toward COVID-19 and influenza vaccination uptake in medically at risk children – EPIC study protocol of randomised controlled trials in Australian paediatric outpatient clinics

Journal:	*BMJ Open*
Manuscript ID	bmjopen-2023-076194.R3
Article Type:	Protocol
Date Submitted by the Author:	22-Jan-2024
Complete List of Authors:	Wang, Bing; The University of Adelaide, Robinson Research Institute and Adelaide Medical School<br>Andraweera, Prabha ; University of Adelaide School of Medicine, Obstetrics and Gynaecology<br>Danchin, Margaret; Murdoch Childrens Research Institute, Paediatrics<br>Blyth, Christopher C.; The University of Western Australia<br>Vlaev, Ivo; Warwick Business School<br>Ong, Jason ; Monash University, Melbourne Sexual Health Clinic & LSHTM<br>Dodd, Jodie; The University of Adelaide, Obstetrics and Gynaecology<br>Couper, Jennifer; The University of Adelaide<br>Sullivan, Thomas; South Australian Health and Medical Research Institute<br>Karnon, Jonathan; Flinders University, CMPH<br>Spurrier, Nicola; SA Health Department for Health and Wellbeing<br>Cusack, Michael; SA Health Department for Health and Wellbeing<br>Mordaunt, Dylan; Flinders University<br>Simatos, Dimi; Discipline of Paediatrics Lyell McEwin Hospital<br>Dekker, Gustaaf; The University of Adelaide<br>Carlson, Samantha; Telethon Kids Institute, Wesfarmers Centre of Vaccines and Infectious Diseases; The University of Western Australia, School of Social Sciences<br>Tuckerman, Jane; Murdoch Childrens Research Institute, Infection and Immunity<br>Wood, Nicholas; University of New South Wales<br>Whop, Lisa; Australian National University, National Centre for Epidemiology and Population Health<br>Marshall, Helen; The University of Adelaide; The University of Adelaide
**Primary Subject Heading**:	Infectious diseases
Secondary Subject Heading:	Public health, Infectious diseases
Keywords:	INFECTIOUS DISEASES, Public health < INFECTIOUS DISEASES, Primary Prevention, COVID-19

TITLE PAGE

Nudging toward COVID-19 and influenza vaccination uptake in medically at risk children – EPIC study protocol of randomised controlled trials in Australian paediatric outpatient clinics

Bing Wang[1,2,3], Prabha H Andraweera[1,2,3], Margie Danchin[4,5], Christopher C Blyth[6,7,8], Ivo Vlaev[9], Jason J. Ong[10], Jodie Dodd[2,3,11], Jennifer Couper[2,3,12], Thomas R Sullivan[13,14], Jonathan Karnon[15], Nicola Spurrier[16,17], Michael Cusack[17], Dylan Mordaunt[16], Dimi Simatos[18], Gus Dekker[2,3,19], Samantha Carlson[6], Jane Tuckerman[4,5,], Nicholas Wood[20,21], Lisa Whop[22], Helen S Marshall[1,2,3]

[1] Vaccinology and Immunology Research Trials Unit, Women's and Children's Hospital, SA Health, Adelaide, South Australia

[2] Robinson Research Institute, The University of Adelaide, Adelaide, South Australia, Australia

[3] Adelaide Medical School, The University of Adelaide, Adelaide, South Australia, Australia

[4] Murdoch Childrens Research Institute, Melbourne, Victoria, Australia; The Royal Children's Hospital, Melbourne, Victoria, Australia

[5] Department of Paediatrics, The University of Melbourne, Melbourne, Australia

[6] Wesfarmers Centre of Vaccines and Infectious Diseases, Telethon Kids Institute and School of Medicine, University of Western Australia, Western Australia, Australia

[7] Department of Infectious Diseases, Perth Children's Hospital, Perth, Western Australia, Australia

[8] Department of Microbiology, PathWest Laboratory Medicine, Queen Elizabeth II Medical Centre (QEIIMC), Western Australia, Australia

For peer review only - http://bmjopen.bmj.com/site/about/guidelines.xhtml
For peer review only

[9] School of Business, Warwick University, Warwick, United Kingdom

[10] Central Clinical School, Monash University, Melbourne, Victoria, Australia

[11] Women's and Babies Division, Women's and Children's Hospital, Adelaide, South Australia, Australia

[12] Division of Paediatrics, Women's and Children's Hospital, Adelaide, South Australia, Australia

[13] South Australian Health and Medical Research Institute, South Australia, Australia

[14] School of Public Health, The University of Adelaide, Adelaide, South Australia, Australia

[15] Discipline of Public Health, Flinders University, South Australia, Australia

[16] Discipline of Paediatrics, Flinders University, South Australia, Australia

[17] SA Health, South Australian Government, Australia

[18] Discipline of Paediatrics, Lyell McEwin Hospital, South Australia, Australia

[19] Discipline of Women's Health, Lyell McEwin Hospital, South Australia, Australia

[20] Discipline of Paediatrics, University of Sydney, Sydney, New South Wales, Australia

[21] Children's Hospital Westmead, Sydney, New South Wales, Australia

[22] Discipline of Public Health, Australian National University, Canberra,

Key words: COVID-19, Influenza, vaccine, medically at risk children

Correspondence: Prof. Helen Marshall, Vaccinology and Immunology Research Trials Unit, Women's and Children's Hospital, Adelaide, South Australia, Australia; phone +61 (08) 8313 4086, fax +61 (08) 8313 4086, email helen.marshall@adelaide.edu.au

For peer review only - http://bmjopen.bmj.com/site/about/guidelines.xhtml

ABSTRACT

Introduction: Children with chronic medical diseases are at an unacceptable risk of hospitalisation and death from influenza and coronavirus 2 (SARS-CoV-2) infections. Over the past two decades, behavioural scientists have learnt how to design non-coercive "nudge" interventions to encourage positive health behaviours. Our study aims to evaluate the impact of multi-component nudge interventions on the uptake of coronavirus disease (COVID-19) and influenza vaccines in medically at risk children.

Methods and analyses: Two separate randomized controlled trials (RCTs), each with 1038 children, will enrol a total of approximately 2076 children with chronic medical conditions who are attending tertiary hospitals in South Australia, Western Australia, and Victoria. Participants will be randomly assigned (1:1) to the standard care or intervention group. The nudge intervention in each RCT will consist of three text message reminders with four behavioural nudges including (i) social norm messages, (ii) different messengers through links to short educational videos from a paediatrician, medically at risk child and parent, and nurse, (iii) a pledge to have their child or themselves vaccinated and (iv) information salience through links to the current guidelines and vaccine safety information. The primary outcome is the proportion of medically at risk children who receive at least one dose of vaccine within 3 months of randomisation. Logistic regression analysis will be performed to determine the effect of the intervention on the probability of vaccination uptake.

Ethics and dissemination: The protocol and study documents have been reviewed and approved by the Women's and Children's Health Network Human Research Ethics Committee (HREC/22/WCHN/2022/00082). Results will be published via peer-reviewed journals and presented at scientific meetings and public forums.

ClinicalTrials.gov Identifier: NCT05613751

For peer review only

For peer review only - http://bmjopen.bmj.com/site/about/guidelines.xhtml

Article Summary

Strengths and limitations of this study

- The Nudgeathon involved collaboration with various stakeholders who co-designed the nudge interventions.
- Nudge interventions are currently undergoing evaluation in randomized controlled trials, with an additional assessment of cost-effectiveness.
- The waiver of consent in our study helps mitigate selection bias.
- The general public has experienced multiple COVID-19 vaccine campaigns, which may lead to fatigue with any further COVID-19 promotion strategies.

For peer review only

For peer review only - http://bmjopen.bmj.com/site/about/guidelines.xhtml

INTRODUCTION

Children with chronic medical conditions represent a clinically vulnerable group in Australia with around 15% of children having either a respiratory, cardiac or neurological condition that increases their risk of hospitalisation and death from respiratory infections.[1] A systematic review and meta-analysis conducted by our group in 2017 demonstrates that compared with healthy peers, medically at risk children with influenza are at higher risks for intensive care unit (ICU) admission, mechanical ventilation and death.[2] They are also more likely to develop bacterial pneumonia or experience prolonged hospital length of stay.[2] Australian data on children ≤ 16 years admitted with acute respiratory illnesses and tested for influenza at sentinel hospitals between 2010 and 2019 demonstrate that comorbidities are an independent predictor of severe outcomes. Specifically, the odds of ICU admission are higher in those with any comorbidity (adjusted odds ratio (aOR): 1.36, 95% Confidence Interval [95% CI]: 1.05-1.77) compared with cases without any comorbidity. Children with diabetes (aOR: 3.22, 95% CI: 1.25; 8.23), cardiac (aOR: 1.93, 95% CI: 1.23-3.03), respiratory (aOR: 1.54, 95% CI: 1.08-2.21), or neurological comorbidities (aOR: 1.57, 95% CI: 1.25-1.98) are at greatest odds of ICU admission. Respiratory, neurological, cardiac, genetic, and hepatic comorbidities and diabetes were shown to be associated with prolonged hospitalisation, whereas respiratory, renal, cardiac, and other comorbidities increased the length of ICU stay.[3] The cost per episode of influenza-associated hospitalisation was estimated to be AUD $19,704 (95% CI: 11,715-27,693) for children with chronic lung conditions compared to AUD $4,557 (95% CI: 4,129-4,984) for children without.[4] Influenza vaccines are provided free to this group (≥ 6 months).[5] Our research has previously shown that parents of children with chronic medical conditions are less likely to have their children vaccinated unless recommended by their specialist.[6] The uptake of influenza vaccine at least once in the last two years among medically at risk children is 50% with annual vaccination only 32.8%.[7]

For peer review only - http://bmjopen.bmj.com/site/about/guidelines.xhtml

The coronavirus 2 (SARS-CoV-2) infection causing Coronavirus disease (COVID-19) may overall be milder in children compared to adults but a small proportion develop severe disease and are at risk of a debilitating multisystem-inflammatory syndrome (MIS-C) and/or long COVID (long lasting complications of COVID-19).[8] Children with chronic diseases are also at a higher risk of COVID-19 complications including pneumonia and respiratory failure and long-term health consequences.[4, 9] Long COVID is a multi-system illness characterised by ongoing persistent symptoms that can last for weeks or months following SARS-CoV-2 infection.[10] Chronic fatigue, short term memory problems, loss of taste and smell and long school absences were reported in long COVID cases.[11] COVID-19 vaccines are provided free to medically at risk children (≥ 6 months).[5] Of concern, two-dose COVID-19 vaccine uptake among children aged 5-11 years is at present approximately 40% nationally.[12] At a time when Australia is still experiencing a large number of SARS-CoV-2 infections, with greater risks to the individual and the health system of co-circulation of influenza and SARS-CoV-2 infections and emergence of new viral variants, it is critical that all efforts are made to improve vaccine uptake among these high-risk groups. Equally important is the opportunity to continue to evaluate the safety of COVID-19 vaccines in these groups including children with chronic conditions that are often excluded from clinical trials.

Lack of access and practical barriers to vaccines remain the leading cause of low vaccine uptake, which is especially relevant to patients whose care is primarily in a hospital setting. For children with chronic conditions the hospital often takes on the role of primary as well as tertiary care due to parental anxiety, life pressures, timeliness and access issues. Parents have identified barriers such as concern and lack of confidence in family physicians providing a vaccine recommendation for medically at risk children and access to vaccines in hospital

For peer review only - http://bmjopen.bmj.com/site/about/guidelines.xhtml

settings being sub-optimal. Mobility, transport, language, financial barriers, and remoteness are also reported barriers for children with chronic and complex conditions in accessing services.[13] For these groups, vaccination in the hospital setting, when patients are already onsite and engaged with a healthcare setting, would improve immunisation opportunities and protection against the threat of both influenza and SARS-CoV-2 infection.

Improving uptake of recommended vaccines in medically at risk children primarily attending hospitals requires an innovative rather than a population approach. Over the past two decades, behavioural scientists have learnt how to design non-coercive "nudge" strategies to promote positive behaviours in a range of contexts. In psychology and neuroscience, a dual process theory describes brain functioning as two types of cognitive processes - 'System 1' processes described as automatic, uncontrolled, effortless, associative, fast, unconscious and affective, and 'System 2' processes described as reflective, controlled, effortful, rule-based, slow, conscious and rational.[14] Although this 'dual process' model is considered as a theoretical basis for nudge theory, the nudge approach suggests that automatic decisions can be systematically triggered to improve health outcomes.[15] In other words, nudges are simple cues in our environment that influence people to behave in a certain way to achieve better personal or social goals without making a conscious decision to do so.[16] Using behavioural economics (a discipline that studies how individuals make choice) the environment in which a choice is being made can actively be designed to encourage better health-related choices.[17] A previous study showed nudges and reminders resulted in a decrease in energy-dense nutrient-poor foods in men and sugar-sweetened beverages in women, together with a reduction in body weight.[18] A "nudge" intervention needs to be simple and low cost and if proven successful can be simply incorporated into standard health care. For example, redesigning cardiac rehabilitation referral decision pathway from opt-in to opt-out referral, which automatically

For peer review only

For peer review only - http://bmjopen.bmj.com/site/about/guidelines.xhtml

identified eligible patients from the electronic health record and notified staff on the wards by using secure text messaging, increased cardiac rehabilitation referral rates from 15% to 85%.[19] Nudge-based interventions to address vaccine hesitancy include using reminders and recall, changing the way information is framed and delivered to an intended audience, changing the messenger delivering information, invoking social norms and emotional affect (e.g., through storytelling, dramatic narratives and graphical presentations), and offering incentives or changing defaults. Nudge-based interventions show potential to increase vaccine confidence and uptake. Interestingly, strategies with educational approaches are less effective (or not effective at all) in improving vaccine uptake.[20] The most promising evidence exists for nudges that offer incentives to parents and healthcare workers, that make information more salient, that frame vaccination as the default, or that use trusted messengers to deliver information.[21, 22] Normalizing vaccination and peer influence can activate social tendencies to join others.[23] Several studies have explored the impact of different interventions on vaccination rates and intentions. A large study with 57,893 participants was conducted in a Northern California health system, and found that personal reminder messages increased booster vaccination rates.[24] Among 964,870 participants in 691,820 households in Denmark, two strategies increased influenza vaccination rates.[25, 26] In the US, a study found that short video messages addressing specific COVID-19 vaccine concerns increased vaccination intentions.[27] In a March 2021 study with 1,595 participants in Japan, different messages were tested to encourage COVID-19 vaccination. The 'influence-gain' message was effective for older adults. 'Comparison' and 'influence-loss' messages reinforced existing intentions among older adults.[23] In our previous study conducted at a tertiary paediatric hospital in Adelaide (n=600), a significantly greater proportion receiving the SMS intervention were vaccinated with 38.6% in the SMS intervention group compared with 26.2% in the control group.[28] A recent study was conducted in older adult populations (n=48,125) and found behavioural

For peer review only

For peer review only - http://bmjopen.bmj.com/site/about/guidelines.xhtml

nudges, electronically delivered letters or centralized written reminders, significantly increased influenza vaccination uptake in Finland.[29] A randomized controlled trial (RCT) of a nudge intervention that included text message reminders demonstrated that the first reminder (n=93,354) increased appointment and COVID-19 vaccination rates by 6.07% and 3.57% and that the second reminder (n=67,092) increased those by 1.65% and 1.06% respectively in the early stages of the COVID-19 vaccine rollout.[30] Another RCT was conducted a few months later in a younger population (mean age 39 years; n = 142,428) and no SMS message did substantially better or worse than the control whether vaccination rates were measured one week after the messages were sent or at the end of the study period.[31] The difference between two studies may suggest that nudges help early in vaccination campaigns, but the efficacy decays. However, nudging-based interventions have shown potential to increase vaccine confidence and uptake in many studies, but further evidence is needed for the development and evaluation of clear recommendations.[22]

Our team recently conducted an RCT from April 15 to September 30, 2021 and found a 47% relative increase in uptake of influenza vaccine in medically at risk children using a simple SMS nudge co-designed with paediatricians.[28] If our text based nudges with links to short educational videos prove to be successful in improving influenza and COVID-19 vaccine uptake in medically at risk children, they can easily be implemented as a promising, valuable, low-cost and long-term tool at a national level.

However, a challenge is how behavioural economic concepts and principles can be effectively applied by local community stakeholders to identify barriers and create an innovative intervention to complement the current immunisation programs. To bridge this gap, a Nudgeathon,[32] a crowdsourcing interaction in which decision groups draw on insights and

For peer review only - http://bmjopen.bmj.com/site/about/guidelines.xhtml

methods from nudge theory, augmented by design thinking and drama theory to devise implementable solutions to major behavioural policy problems, was launched.[32] These events are typically conducted over 1-2 days, bringing together a diverse range of stakeholders (many who are new to behavioural science) to generate new approaches to complex problems in a time-pressured setting. Three dozen Nudgeathons [32] around the world have been conducted to address multiple issues (e.g. handwashing among health providers, increasing regular HIV/sexually transmitted infection testing in men [17, 33]) but to our knowledge, there has not been a Nudgeathon for the immunisation programs. The MINDSPACE framework, which provides a list of behaviour change techniques including Messenger, Incentives, Norms, Defaults, Salience, Priming, Affect, Commitments, Ego, that target the automatic decision processes,[34] was used to identify and generate potential nudges at the Nudgeathon. Our goal through the Nudgeathons is to gain valuable "Customer Insight" within the Australian context, fostering a profound understanding of parents' experiences, beliefs, needs, and desires, while also identifying the practical and structural challenges they encounter. It's important to recognise that attempts to promote behaviour change without considering these contextual factors often result in frustration. Behaviour change initiatives can be contentious, involving intricate trade-offs and often addressing areas where government decisions are controversial such as COVID-19 vaccination policies. Consequently, innovative approaches like Nudgeathons may be essential to engage the public effectively in exploring acceptable courses of action.

To our knowledge, this will be the first study assessing the effectiveness and cost-effectiveness of a co-designed multi-component nudge intervention on improving influenza and COVID-19 vaccine uptake in medically at-risk children. Vaccine hesitancy and access barriers are a major barrier that reduces the progress of management of vaccine preventable diseases in children.

For peer review only

For peer review only - http://bmjopen.bmj.com/site/about/guidelines.xhtml

235 Identifying solutions to improving COVID-19 and influenza vaccine uptake in medically at risk children is critical, as these children are more vulnerable to severe COVID-19 and influenza than healthy children.

239 Therefore, this study aims to design nudge interventions and evaluate the impact of multi-component nudge interventions on the uptake of influenza and COVID-19 vaccines among medically at risk children.

METHODS AND ANALYSIS

244 Recommendations for Intervention Trials (SPIRIT) checklist was used when developing this protocol.[35] The first participant was randomised on 29 November 2022 and the study is expected to be complete by 31 December 2024. The master protocol was devised to evaluate the impact of multi-component nudge interventions on the uptake of COVID-19 and influenza vaccines in two distinct sub-populations: medically at-risk children and pregnant women, employing a unified and overarching design. The information pertaining to medically at-risk children is presented here. The protocol information regarding the sub-study involving pregnant women was published elsewhere.[36]

Nudge intervention

254 In August 2022, a Nudgeathon was held to design appropriate nudges for inclusion in the trial. Fourteen participants including medically at risk children, their parents, paediatricians, nurses, hospital administrative personnel, behavioural scientists, psychologists and graphic designers with diverse skills from South Australia (SA), Western Australia and Victoria participated in the Nudgeathon. The participants learnt about behavioural science and successful application of nudges in different disciplines. They were assigned into small groups ensuring that each

For peer review only

For peer review only - http://bmjopen.bmj.com/site/about/guidelines.xhtml

group comprised participants with different skills. The individual groups were requested to create a nudge for improving either COVID-19 or influenza vaccine uptake in medically at risk children. At the end of the Nudgeathon each group presented their nudges and one nudge for each condition was chosen by the team to develop the nudge interventions in the following randomised control trials. Some of the most robust effects that have been found to have strong impacts on behaviour[34] such as Messenger, Norms, Salience and Commitments were used to generate nudge interventions in our study. The carefully chosen nudge for both COVID-19 and influenza vaccines consists of three SMS text messages that are sent to parents of medically at risk children from the participating hospitals four weeks apart. The first text message is sent one week prior to a scheduled paediatric clinic appointment and provides a link to current national guidelines on COVID-19 or influenza vaccination in children with chronic illness and a link to a video of a paediatrician explaining the benefits of COVID-19 or influenza vaccination in medically at risk children. The second text message provides a link to a video of a child with chronic illness and their parent giving their opinion on the protection of medically as risk children against SARS-CoV-2 or influenza infections. The third text message provides a link to a video of a nurse discussing the potential adverse outcomes of not being vaccinated against SARS-CoV-2 or influenza infections. The first and second text messages also provide the option for a parent to agree to vaccinate their children or opt out from receiving further reminders.

The study is aligned with the MINDSPACE Framework, a behavioural change model developed by the UK government's Behavioural Insights Team in 2010. The decision to incorporate a blend of behavioural nudges in the intervention stems from the notion that a multifaceted approach can potentially yield a more substantial impact on behaviour. Combining various nudges is often considered a strategy to enhance the likelihood of success,

For peer review only - http://bmjopen.bmj.com/site/about/guidelines.xhtml

as different individuals may respond diversely to different nudge effects.[37, 38] This approach seeks to cast a broader net to appeal to a more extensive spectrum of people and behaviours, leveraging the effects encapsulated in the acronym MINDSPACE, which includes Messenger, Incentives, Norms, Defaults, Salience, Priming, Affect, Commitment, and Ego. Significantly, there is a considerable overlap among these effects, and the most effective interventions will invariably integrate various elements.

Primary objectives

- To determine the proportion of medically as risk children in the intervention versus standard care (non-intervention) group receiving at least one dose of the seasonal influenza vaccine within 3 months after randomisation, as assessed using the Australian Immunisation Register (AIR)
- To determine the proportion of medically as risk children in the intervention versus standard care (non-intervention) group receiving at least one dose of a COVID-19 vaccine within 3 months after randomisation, as assessed using the AIR.

The AIR is a national register that records vaccines given to all people in Australia including vaccines given under the National Immunisation Program, including school programs, and privately, such as for influenza. A recognised vaccination provider including doctors, such as a family physician or community health centre, are required to update the AIR once an influenza, COVID-19 or any vaccine listed in the National Immunisation Program Schedule is administered.

Secondary objectives

For peer review only - http://bmjopen.bmj.com/site/about/guidelines.xhtml

- To assess the number of medically at risk children who received COVID-19 or influenza vaccines, change from baseline up to three months post-randomisation, based on socio-demographic characteristics
- To assess timeliness of influenza and COVID-19 vaccine uptake among medically at risk children during the study period by determining the proportion of medically at risk children who receive the COVID-19 or influenza vaccine by month throughout the study period.
- To estimate the cost-effectiveness of proven interventions compared to standard care in hospital settings

Study design and study population

These are parallel-group randomised controlled trials (RCTs, ClinicalTrials.gov Identifier: NCT05613751) designed to measure the impact of a nudge intervention on receipt of one dose of the COVID-19 or influenza vaccine in medically at risk children who attend a clinic at tertiary hospitals in South Australia, Western Australia and Victoria. Medically at risk children will be enrolled at seven hospitals across South Australia (Women's and Children's Hospital, Flinders Medical Centre and the Lyell McEwin Hospital), Victoria (The Royal Children's Hospital) and Western Australia (Perth Children's Hospital). Each of these hospitals covers different socio-demographic areas and patient sources ensuring our research is generalisable. COVID-19 or influenza vaccination status of medically at risk children attending the participating hospitals will be determined by checking the AIR. The inclusion and exclusion criteria are listed in Table 1.

For peer review only - http://bmjopen.bmj.com/site/about/guidelines.xhtml

331 Table 1 Inclusion and exclusion criteria

	Exclusion criteria	Inclusion criteria
COVID vaccine [39]	• Known contraindications to COVID-19 vaccine<br>• Up to date for COVID-19 vaccine (≥ two doses) at the time of enrolment,<br>• Sibling of a child already enrolled in the trial (only the sibling who is eligible and scheduled to attend a paediatric clinic first will be eligible)<br>• Previous participation in the influenza nudge RCT	Medically at risk children aged 5 years to 18 years with a cardiac, endocrine, respiratory, gastrointestinal, haematological, musculoskeletal, neurological condition
Influenza vaccine [5]	• Known contraindications to influenza vaccine<br>• Already received an influenza vaccine during the flu season in 2023<br>• Sibling of a child already participating in the trial (the sibling who is eligible and scheduled to attend a paediatric clinic first will be eligible)	Children aged ≥6 months and < 18 years with medical conditions specified in this list: immunocompromising conditions including malignancy, chronic steroid use, haematopoietic stem cell transplant; functional or anatomical asplenia including sickle cell disease or other haemoglobinopathies, congenital or acquired asplenia (for example, splenectomy) or hyposplenia; cardiac disease including cyanotic congenital heart disease, congestive heart failure, coronary

For peer review only - http://bmjopen.bmj.com/site/about/guidelines.xhtml

	Exclusion criteria	Inclusion criteria
	• Previous participation in the COVID-19 nudge RCT	artery disease; chronic respiratory conditions including suppurative lung disease, bronchiectasis, cystic fibrosis, chronic obstructive pulmonary disease, severe asthma (requiring frequent medical consultations or the use of multiple medicines); chronic neurological conditions including hereditary and degenerative CNS diseases, seizure disorders, spinal cord injuries, neuromuscular disorders; chronic metabolic disorders including Type 1 or 2 diabetes, amino acid disorders, carbohydrate disorders, cholesterol biosynthesis disorders, fatty acid oxidation defects, lactic acidosis, mitochondrial disorders, organic acid disorders, urea cycle disorders, vitamin/cofactor disorders, porphyria; chronic renal failure; children aged 5 to 10 years receiving long term aspiring therapy; Down syndrome; obesity (body mass index ≥30 kg/m2); children born less than 37 weeks gestation

332

For peer review only - http://bmjopen.bmj.com/site/about/guidelines.xhtml

Randomisation

Medically at risk children will be randomised to the intervention and standard care groups in a 1:1 ratio. The randomisation list will be generated using R version 4.02 by an independent statistician who will not be involved with the conduct or analysis of the RCT. Allocations will be performed using randomly permuted blocks, stratified by hospital (i.e., separate randomisation sequence used in each hospital). Randomisation will be done on a password protected REDCap database securely held on the University of Adelaide server.

Endpoint measurements will involve low level contact of study staff, however when contact is required (such as AIR/ confirmation of influenza/COVID-19 vaccine receipt etc), this will be carried out by trial staff shielded from information that might reveal trial group assignment. The study statistician undertaking the analysis and study investigators will remain blinded to trial intervention assignment.

Study processes

Medically at risk children attending clinics at the hospitals will be identified from Outpatients' Department's appointment lists. Research nurses will assess COVID-19 or influenza vaccination status of children on the AIR to screen for eligibility. Medically at risk children will be randomised to the intervention arm or the standard care arm on REDCap. Demographic data will be obtained from hospital medical records. Using the Norms, Messenger, Salience and Commitment nudge strategies, parents of medically at risk children randomised to the intervention arm will receive a maximum of three SMS reminders from the hospital using 'Message Media' software from the hospitals. The SMS messages will comprise a brief message that many children with chronic illness obtain the COVID-19 or influenza vaccine (Norms) and a reminder for the child to obtain the vaccine. Parents of medically at risk children

For peer review only - http://bmjopen.bmj.com/site/about/guidelines.xhtml

have the option of responding to the message by 1) agreeing to obtain the vaccine (Commitment), 2) stating that the participant has already received the vaccine or 3) requesting to opt out from receiving further reminders (first and second SMS messages). Each SMS message also provides a link to a video of (Messager): 1) a paediatrician discussing the potential serious health consequences of SARS-CoV-2 or influenza infections (first SMS), 2) a medically at risk child and their parent stating that the child received the vaccine and that the child is protected from serious adverse effects of SARS-CoV-2 or influenza infections (second SMS), 3) a nurse stating the benefits of COVID-19 or influenza vaccination for medically at risk children (third SMS). In addition, the first SMS also states children with special medical conditions are at increased risk of severe COVID-19 or Influenza disease and provides a link to the current guidelines and safety information about COVID-19 or influenza vaccination (Salience). The paediatrician and nurse assumed the roles of healthcare experts, while the child with medical risks and their parent acted as relatable peers with a similar background. They served as influential messengers in persuading parents to vaccinate their children. The message highlighting the fact that many children with chronic illnesses receive the COVID-19 or influenza vaccine utilised a social norms approach, conveying what other parents in similar situations typically do. Meanwhile, the message emphasising the increased risk of severe COVID-19 or influenza for children with special medical conditions was designed to capture parents' attention, as it is information more likely to be comprehensible and directly relevant to their own children. The response options were strategically crafted to foster reciprocity and serve as commitments. The first SMS is sent approximately one week prior to a scheduled clinic visit. The second SMS message is sent four weeks after the first message to those who have not received a dose of COVID-19 or influenza vaccine after the first SMS (confirmed on AIR) and not requested to opt out. The third SMS message is sent four weeks after the second message to those who have not received a dose of COVID-19 or influenza vaccine after the

For peer review only - http://bmjopen.bmj.com/site/about/guidelines.xhtml

second SMS (confirmed on AIR) and not requested to opt out. The AIR will be checked at 3 months after randomisation to assess whether medically at risk children have received a dose of COVID-19 or influenza vaccine. All data will be stored securely on a password protected REDCap database held by the University of Adelaide. All data will be de-identified prior to presentation and publication.

Study monitoring and surveillance

The nudge is a behavioural change intervention and hence there are no risks related to invasive procedures or investigational medications. Any risks of mental distress associated with receiving the text message is expected to be very low and the parents have the option of opting out from receiving the further text messages after the first one. However, a risk assessment and management plan has been developed for this study. Moreover, the study management committee comprising the chief investigator, site investigators, study coordinators and statistician will carefully monitor the progress of the study.

This trial has certain limitations. The nudge interventions incorporated a blend of various nudge techniques, and we did not assess the individual effects of these techniques in our study. We included links within the text messages, and it was the parents' responsibility to click on these links. If the links were not clicked, the nudge interventions may not have been as effectively implemented as originally intended.

Sample size and statistical analysis plan

To detect an increase in the vaccination rate from ~50% in the control group to 60% in the intervention group, which is considered a clinically relevant impact, with 90% power (two-sided test with alpha = 0.05), a sample size of n = 519 per group (1038 total) is required. Should

For peer review only - http://bmjopen.bmj.com/site/about/guidelines.xhtml

the vaccination rate in the control arm be higher than 50% or lower than 40%, this sample size will still provide at least 90% power to detect a 10% absolute increase in vaccination with the nudge intervention. We aim to enrol at least 1038 medically at risk children to each of the two RCTs across the participating hospitals.

Statistical analyses will be conducted on an Intention To Treat basis according to a pre-specified statistical analysis plan. Baseline characteristics including age, socio-economic status based on postcode, and ethnicity will be reported for each group using percentages, means with standard deviations or medians with ranges as appropriate. The vaccination rate will be compared between intervention and control groups using logistic regression, with adjustment only made for participating hospitals (treated as a categorical fixed effect). Intervention effects will be reported as odds ratios with 95% confidence intervals. Patient characteristics associated with vaccination will be investigated using multivariable logistic regression models. Cost-effectiveness analysis of the nudge intervention will be compared to standard care from the healthcare payer perspective. The primary cost-effectiveness analysis will estimate the incremental cost per additional person vaccinated, in each of the two RCTs. Secondary analyses for the trial focused on influenza vaccination will estimate the cost per quality-adjusted life year (QALY) gained. The study results will be used to estimate the impact of the nudge intervention on health-related quality of life by calculating the QALYs gained as a result of the increased vaccine uptake. Implementation costs will be obtained from the study budget and costs related to research activities will be excluded. Estimated cost offsets to the health system associated with influenza related disease (e.g. hospitalisations and emergency visits) will be derived from the literature and calculated using cost weights for Australian Refined Diagnosis Related Groups (AR-DRGs). The cost-effectiveness evaluation will follow standard reporting guidelines in the CHEERS statement.[40]

For peer review only - http://bmjopen.bmj.com/site/about/guidelines.xhtml

Patient and Public Involvement

In our previous survey [6], 539 parents were interviewed, revealing that parents of children with chronic medical conditions were less likely to vaccinate their children unless recommended by a specialist. Interventions based on nudging in our previous study [28] showed potential in increasing vaccine uptake. Consequently, the study aims, design, and outcome measures have been informed by the priorities, experiences, and preferences of parents. Medically at risk children and their parents actively contributed to the study's design during the Nudgeathon. Their insights played a crucial role in designing the nudge interventions, making it more relevant and patient-centred. The study results will be disseminated to the trial participants, which includes medically at risk children and their parents, through various accessible means, such as academic publication and lay summaries on our research unit's webpage, to ensure that the findings are easily understood and meaningful to this group.

ETHICS AND DISSEMINATION

The current protocol version 2.0 (05-01-2023) and all study material have been reviewed and approved by the Women's and Children's Health Network Human Research Ethics Committee (HREC/2022/00082) and research governance approval has been obtained from the Women's and Children's Hospital, Flinders Medical Centre, and Lyell McEwin Hospital in SA, The Royal Children's Hospital in VIC, and Perth Children's Hospital in Western Australia. The written consent was obtained from all stakeholders including health service leaders and clinicians and health consumers to participate in the Nudgeathons. A waiver of consent was approved for medically at risk children to participate in this trial and receive SMS messages in accordance with the National Statement on Ethical Conduct in Human Research. Three criteria

For peer review only - http://bmjopen.bmj.com/site/about/guidelines.xhtml

guide this. Firstly, there are no estimated risks or harms associated with study procedures. Any risk of psychosocial distress associated with receiving the nudge interventions is very unlikely. Secondly, it would not be possible to obtain informed consent without threatening the validity of the trials. It would be impossible to assess the effectiveness of the nudge by recruiting parents of medically at risk children and informing them about the study and then randomising them not to receive the nudge. Thirdly, as influenza and COVID-19 vaccines are recommended for all medically at risk children aged ≥ 6 months, all data for the influenza and COVID-19 RCTs will be collected as part of routine care, including patient demographics and specialties seen. In addition this methodology was approved and successfully applied in the Flutext-4U study.[41] We identified a significant difference in uptake of influenza vaccine in medically at risk children in the Flutext-4U study, likely due to this being a population inclusive study unaffected by healthy selection bias.[28] The study is being conducted in accordance with the Declaration of Helsinki and the International Conference on Harmonization Guidelines on Good Clinical Practice. The trial is being conducted in compliance with the current version of the protocol. Any change to the protocol that affects scientific content, study design, and participating will be considered an amendment and will be submitted to HREC for review and approval prior to implementation.

Participant confidentiality is strictly held in trust by the investigators. Therefore, study documentation, data, and all other information generated will be held in strict confidence. No information concerning the study, or the data will be released to any unauthorised third party. Study participant research data, which is for purposes of statistical analysis and scientific reporting, will be transmitted to and stored at the Women's and Children's Hospital. At the end of the study, all study databases will be de-identified and archived at the University of Adelaide. After the completion of the study, the de-identified and aggregated results will be

For peer review only - http://bmjopen.bmj.com/site/about/guidelines.xhtml

presented at scientific forums and submitted for publication in peer-reviewed journals. The results will be disseminated regardless of the direction of effect. The findings of the study will be communicated to key stakeholders.

The burden of the intervention is planned to be assessed with input from medically at risk children's parents using a short evaluation questionnaire. Special recognition and appreciation were extended to the medical at risk children and their parents who participated in the Nudgeathon to highlight their valuable contributions to the study's design and success.

For peer review only

For peer review only - http://bmjopen.bmj.com/site/about/guidelines.xhtml

AUTHORS CONTRIBUTIONS

HM, IV, JO, MD, CB, BW, PA, JD, JC, TS, JK, NS, MC, DM, DS, GD, SC, JT, NW and LW conceptualised the interventions, contributed to the design of the study protocol, and acquired funding. TS provided the statistical consult. BW drafted the manuscript. MD, CB, JD, JC, JK, NS, MC, DM, DS, GD, SC, NW and LW critically reviewed. HM, BW, IV, JO, TS, JT and PA critically reviewed and edited the manuscript. HM, BW, IV, JO, MD, CB, PA, JD, JC, TS, JK, NS, MC, DM, DS, GD, SC, JT, NW and LW approved its final version.

For peer review only

ACKNOWLEDGEMENTS

We acknowledge the following study coordinators for their contribution to study set up and recruitment: Kirsty Herewane (Women's and Children's Hospital), Joanne Koch (Lyell McEwin Hospital), Lauren Thompson (Flinders Medical Centre), Ashleigh Rak (Royal Children's Hospital), Deborah Pidd (Mercy Hospital for Women), Louisa Paparo, Rebecca Pavlos and Erin Van Der Helder (Perth Children's Hospital). We would also like to express our gratitude to all participants who were involved in this study.

FUNDING STATEMENT

This work is supported by the National Health and medical Research Council of Australia [NHMRC Partnership grant APP2014684]. Additional in-kind funding is provided by funding partners including the Department of Health, South Australia Government; Department of Health, Victorian Government; Department of Health, Western Australian Government; Women's and Children's Health Network; Southern Adelaide Local Health Network; Northern Adelaide Local Health Network; Women's and Children's Hospital Foundation; Department of Trade and Investment, South Australian Government, in support of the NHMRC Partnership grant.

For peer review only - http://bmjopen.bmj.com/site/about/guidelines.xhtml

516

517 **COMPETING INTERESTS STATEMENT**

518 HM acknowledges support from the National Health and Medical Research Council of
519 Australia: Practitioner Fellowship (APP1155066). CCB acknowledges support from the
520 National Health and Medical Research Council of Australia: Investigator Grant
521 (APP1173163). HM is an independent investigator on clinical trials of investigational vaccines
522 manufactured by pharmaceutical companies including Pfizer, ILiAD Biotechnologies, and
523 Merck. The institution at which HM, BW and PA are employed has received funding for
524 investigator-led research from GlaxoSmithKline, Sanofi-Pasteur, and Pfizer Vaccines. All
525 authors receive no personal payments from industry. There are no other conflicts of interest to
526 declare.

For peer review only

For peer review only - http://bmjopen.bmj.com/site/about/guidelines.xhtml

REFERENCES

[1] Australian Institute of Health and Welfare 2020. Australia’s children. Cat. no. CWS 69. Canberra: AIHW. https://www.aihw.gov.au/reports/children-youth/australias-children/contents/executive-summary. Australia's Children. DOI: 10.25816/5ebca4d0fa7dd

[2] Tuckerman J, Misan S, Crawford NW, Marshall HS. Influenza in Children With Special Risk Medical Conditions: A Systematic Review and Meta-analysis. The Pediatric infectious disease journal. 2019;38:912-9.

[3] Norman DA, Cheng AC, Macartney KK, Moore HC, Danchin M, Seale H, et al. Influenza hospitalizations in Australian children 2010-2019: The impact of medical comorbidities on outcomes, vaccine coverage, and effectiveness. Influenza and other respiratory viruses. 2022;16:316-27.

[4] Homaira N, Briggs N, Oei JL, Hilder L, Bajuk B, Snelling T, et al. Impact of influenza on hospitalization rates in children with a range of chronic lung diseases. Influenza and other respiratory viruses. 2019;13:233-9.

[5] Australian Technical Advisory Group on Immunisation (ATAGI). Australian Immunisation Handbook, Australian Government Department of Health and Aged Care, Canberra, 2022, immunisationhandbook.health.gov.au

[6] Tuckerman J, Crawford NW, Marshall HS. Disparities in parental awareness of children's seasonal influenza vaccination recommendations and influencers of vaccination. PloS one. 2020;15:e0230425.

[7] Tuckerman J, Misan S, Salih S, Joseph Xavier B, Crawford NW, Lynch J, et al. Influenza vaccination: Uptake and associations in a cross-sectional study of children with special risk medical conditions. Vaccine. 2018;36:8138-47.

[8] Sudre CH, Murray B, Varsavsky T, Graham MS, Penfold RS, Bowyer RC, et al. Attributes and predictors of long COVID. Nature medicine. 2021;27:626-31.

For peer review only

For peer review only - http://bmjopen.bmj.com/site/about/guidelines.xhtml

[9] Moeller A, Thanikkel L, Duijts L, Gaillard EA, Garcia-Marcos L, Kantar A, et al. COVID-19 in children with underlying chronic respiratory diseases: survey results from 174 centres. ERJ open research. 2020;6.

[10] Australian Institute of Health and Welfare (2022) Long COVID in Australia – a review of the literature, catalogue number PHE 318, AIHW, Australian Government.

[11] Ludvigsson JF. Case report and systematic review suggest that children may experience similar long-term effects to adults after clinical COVID-19. Acta paediatrica (Oslo, Norway : 1992). 2021;110:914-21.

[12] https://www.health.gov.au/sites/default/files/2023-03/covid-19-vaccine-rollout-update-3-march-2023.pdf. COVID-19 Vaccine Roll-out (02 March 2023).

[13] Pearce A, Marshall H, Bedford H, Lynch J. Barriers to childhood immunisation: Findings from the Longitudinal Study of Australian Children. Vaccine. 2015;33:3377-83.

[14] Vlaev I, King D, Dolan P, Darzi A. The Theory and Practice of "Nudging": Changing Health Behaviors. Public Administration Review. 2016;76:550-61.

[15] Marteau TM, Ogilvie D, Roland M, Suhrcke M, Kelly MP. Judging nudging: can nudging improve population health? BMJ (Clinical research ed). 2011;342:d228.

[16] Simon C, Tagliabue M. Feeding the behavioral revolution: Contributions of behavior analysis to nudging and vice versa. Journal of Behavioral Economics for Policy. 2018;2:91-7.

[17] Ong JJ, Chow EPF, Read D, Taj U, Lee D, Vlaev I. Nudgeathons to control HIV: designing strategies using behavioural economics. AIDS. 2020;34:2337-40.

[18] Kerr DA, Harray AJ, Pollard CM, Dhaliwal SS, Delp EJ, Howat PA, et al. The connecting health and technology study: a 6-month randomized controlled trial to improve nutrition behaviours using a mobile food record and text messaging support in young adults. Int J Behav Nutr Phys Act. 2016;13:52.

For peer review only

For peer review only - http://bmjopen.bmj.com/site/about/guidelines.xhtml

[19] Adusumalli S, Jolly E, Chokshi NP, Gitelman Y, Rareshide CAL, Kolansky DM, et al. Referral Rates for Cardiac Rehabilitation Among Eligible Inpatients After Implementation of a Default Opt-Out Decision Pathway in the Electronic Medical Record. JAMA network open. 2021;4:e2033472.

[20] Dubé E, Gagnon D, MacDonald NE. Strategies intended to address vaccine hesitancy: Review of published reviews. Vaccine. 2015;33:4191-203.

[21] Tentori K, Pighin S, Giovanazzi G, Grignolio A, Timberlake B, Ferro A. Nudging COVID-19 Vaccine Uptake by Changing the Default: A Randomized Controlled Trial. Med Decis Making. 2022;42:837-41.

[22] Renosa MDC, Landicho J, Wachinger J, Dalglish SL, Barnighausen K, Barnighausen T, et al. Nudging toward vaccination: a systematic review. BMJ Glob Health. 2021;6.

[23] Sasaki S, Saito T, Ohtake F. Nudges for COVID-19 voluntary vaccination: How to explain peer information? Soc Sci Med. 2022;292:114561.

[24] Chang TY, Jacobson M, Shah M, Kopetsky M, Pramanik R, Shah SB. Reminders, but not monetary incentives, increase COVID-19 booster uptake. Proc Natl Acad Sci U S A. 2023;120:e2302725120.

[25] Johansen ND, Vaduganathan M, Bhatt AS, Lee SG, Modin D, Claggett BL, et al. Electronic nudges to increase influenza vaccination uptake in Denmark: a nationwide, pragmatic, registry-based, randomised implementation trial. Lancet. 2023;401:1103-14.

[26] Johansen ND, Vaduganathan M, Bhatt AS, Lee SG, Modin D, Claggett BL, et al. Nationwide Utilization of Danish Government Electronic Letter System for Increasing InFLUenza Vaccine Uptake (NUDGE-FLU): Study Protocol for a Nationwide Randomized Implementation Trial. Am Heart J. 2023.

[27] Jensen UT, Ayers S, Koskan AM. Video-based messages to reduce COVID-19 vaccine hesitancy and nudge vaccination intentions. PloS one. 2022;17:e0265736.

For peer review only

For peer review only - http://bmjopen.bmj.com/site/about/guidelines.xhtml

601 [28] Tuckerman J, Harper K, Sullivan TR, Cuthbert AR, Fereday J, Couper J, et al. Short Message Service Reminder Nudge for Parents and Influenza Vaccination Uptake in Children and Adolescents With Special Risk Medical Conditions: The Flutext-4U Randomized Clinical Trial. JAMA pediatrics. 2023.

605 [29] Saaksvuori L, Betsch C, Nohynek H, Salo H, Sivela J, Bohm R. Information nudges for influenza vaccination: Evidence from a large-scale cluster-randomized controlled trial in Finland. PLoS Med. 2022;19:e1003919.

608 [30] Dai H, Saccardo S, Han MA, Roh L, Raja N, Vangala S, et al. Behavioural nudges increase COVID-19 vaccinations. Nature. 2021;597:404-9.

610 [31] Rabb N, Swindal M, Glick D, Bowers J, Tomasulo A, Oyelami Z, et al. Evidence from a statewide vaccination RCT shows the limits of nudges. Nature. 2022;604:E1-E7.

612 [32] Nudgeathon. [cited 27 March 2023]. Available from: https://www.nudgeathon.co.uk..

613 [33] Tran J, Vlaev I, Read D, Schmidtke KA, Chow EP, Lee D, et al. A qualitative evaluation of a Nudgeathon event for the co-design of sexual health campaign images targeting overseas-born men who have sex with men. Sexual Health. 2023.

616 [34] Dolan P, Hallsworth M, Halpern D, King D, Vlaev I. MINDSPACE: influencing behaviour for public policy. 2010.

618 [35] Chan AW, Tetzlaff JM, Gotzsche PC, Altman DG, Mann H, Berlin JA, et al. SPIRIT 2013 explanation and elaboration: guidance for protocols of clinical trials. BMJ (Clinical research ed). 2013;346:e7586.

621 [36] Andraweera PH, Wang B, Danchin M, Blyth C, Vlaev I, Ong J, et al. Randomised controlled trials of behavioural nudges delivered through text messages to increase influenza and COVID-19 vaccines among pregnant women (the EPIC study): study protocol. Trials. 2023;24:454.

For peer review only

For peer review only - http://bmjopen.bmj.com/site/about/guidelines.xhtml

625 [37] Howley P, Ocean N. Can nudging only get you so far? Testing for nudge combination effects. European Review of Agricultural Economics. 2022;49:1086-112.

627 [38] Cosic A, Cosic H, Ille S. Can nudges affect students' green behaviour? A field experiment. Journal of Behavioral Economics for Policy. 2018;2:107-11.

629 [39] Australian Technical Advisory Group on Immunisation (ATAGI). Australian Immunisation Handbook, Australian Government Department of Health and Aged Care, Canberra, 2022, https://www.health.gov.au/initiatives-and-programs/covid-19-vaccines/advice-for-providers/clinical-guidance/clinical-features.

633 [40] Husereau D, Drummond M, Augustovski F, de Bekker-Grob E, Briggs AH, Carswell C, et al. Consolidated Health Economic Evaluation Reporting Standards 2022 (CHEERS 2022) Statement: Updated Reporting Guidance for Health Economic Evaluations. Value Health. 2022;25:3-9.

637 [41] Tuckerman J, Harper K, Sullivan TR, Fereday J, Couper J, Smith N, et al. Using provider-parent strategies to improve influenza vaccination in children and adolescents with special risk medical conditions: a randomised controlled trial protocol. BMJ open. 2022;12:e053838.

640

641

For peer review only

For peer review only - http://bmjopen.bmj.com/site/about/guidelines.xhtml
